# Supplementary material for: Insights into stem Batomorphii: A new holomorphic ray (Chondrichthyes, Elasmobranchii) from the upper Jurassic of Germany
Source: PLoS One. 2025 Jan 23;20(1):e0310174. doi: 10.1371/journal.pone.0310174 (PMC11756912; doi:10.1371/journal.pone.0310174)
Supplement: S1 File — (PDF) [file pone.0310174.s001.pdf]

Supporting material for:

Insights into stem Batomorphii: A new holomorphic ray (Chondrichthyes, Elasmobranchii) from the Upper Jurassic of Germany

JULIA TÜRTSCHER, PATRICK L. JAMBURA, FREDERIK SPINDLER, and JÜRGEN KRIWET

Contents

Supporting tables

Table S1 – Specimens
Table S2 – Traditional morphometrics (% DW): PC axes
Table S3 – Traditional morphometrics (% DW): Loadings
Table S4 – Traditional morphometrics (% DW): Shapiro-Wilk normality test
Table S5 – Traditional morphometrics (% DW): Kruskal-Wallis rank sum test
Table S6 – Traditional morphometrics (% DW): Pairwise Wilcoxon test
Table S7 – Traditional morphometrics (% DW): ANOVA
Table S8 – Traditional morphometrics (% DW): Tukey's honest significance test
Table S9 – Geometric morphometrics (head outline): PC axes
Table S10 – Geometric morphometrics (head outline): Procrustes ANOVA (shape)
Table S11 – Geometric morphometrics (head outline): Pairwise comparison (shape)
Table S12 – Geometric morphometrics (head outline): Procrustes ANOVA (size)
Table S13 – Geometric morphometrics (head outline): Pairwise comparison (size)
Table S14 – Geometric morphometrics (complete body): PC axes
Table S15 – Geometric morphometrics (complete body): Procrustes ANOVA (shape)
Table S16 – Geometric morphometrics (complete body): Pairwise comparison (shape)
Table S17 – Geometric morphometrics (complete body): Procrustes ANOVA (size)
Table S18 – Geometric morphometrics (complete body): Pairwise comparison (size)

Supporting figures for the main text

Figure S1 – Overview of DMA-JP-2010/007 under ultraviolet light

Figure S2 – Traditional morphometrics (% DW): Morphospace occupation & loadings

Figure S3 – Geometric morphometrics (complete body): Morphospace occupation

Supporting material 1

Traditional morphometrics (% DL)

Results

Figure S4 – Morphospace occupation & loadings

Figure S5 – Boxplots of all variables

Table A – PC axes

Table B – Loadings

Table C – Shapiro-Wilk normality test

Table D – Kruskal-Wallis rank sum test

Table E – Pairwise Wilcoxon test

Table F – ANOVA

Table G – Tukey's honest significance test

Supporting material 2

Phylogenetic Analyses

Character list

Matrix

Script

Log file

Figure S6 – Maximum parsimony vs. Maximum likelihood

Supporting material 3

Traditional morphometrics (% DW) data

CSV file – apolithabatis_tm_DW.csv

Traditional morphometrics (% DL) data

CSV file – apolithabatis_tm_DL.csv

Geometric morphometrics (head outline) data

- TPS file – apolithabatis_gm_head.tps
- Sliders file – apolithabatis_gm_head_sliders.csv
- Classifiers file – apolithabatis_gm_head_classifiers.csv

Geometric morphometrics (complete body) data

- TPS file – apolithabatis_gm_body.tps
- Sliders file – apolithabatis_gm_body_sliders.csv
- Classifiers file – apolithabatis_gm_body_classifiers.csv
