# Supplementary material for: Insights into stem Batomorphii: A new holomorphic ray (Chondrichthyes, Elasmobranchii) from the upper Jurassic of Germany
Source: PLoS One. 2025 Jan 23;20(1):e0310174. doi: 10.1371/journal.pone.0310174 (PMC11756912; doi:10.1371/journal.pone.0310174)
Supplement: S3 File — (PDF) [file pone.0310174.s003.pdf]

Supporting figures for:

Insights into stem Batomorphii: A new holomorphic ray (Chondrichthyes, Elasmobranchii) from the Upper Jurassic of Germany

JULIA TÜRTSCHER, PATRICK L. JAMBURA, FREDERIK SPINDLER, and JÜRGEN KRIWET

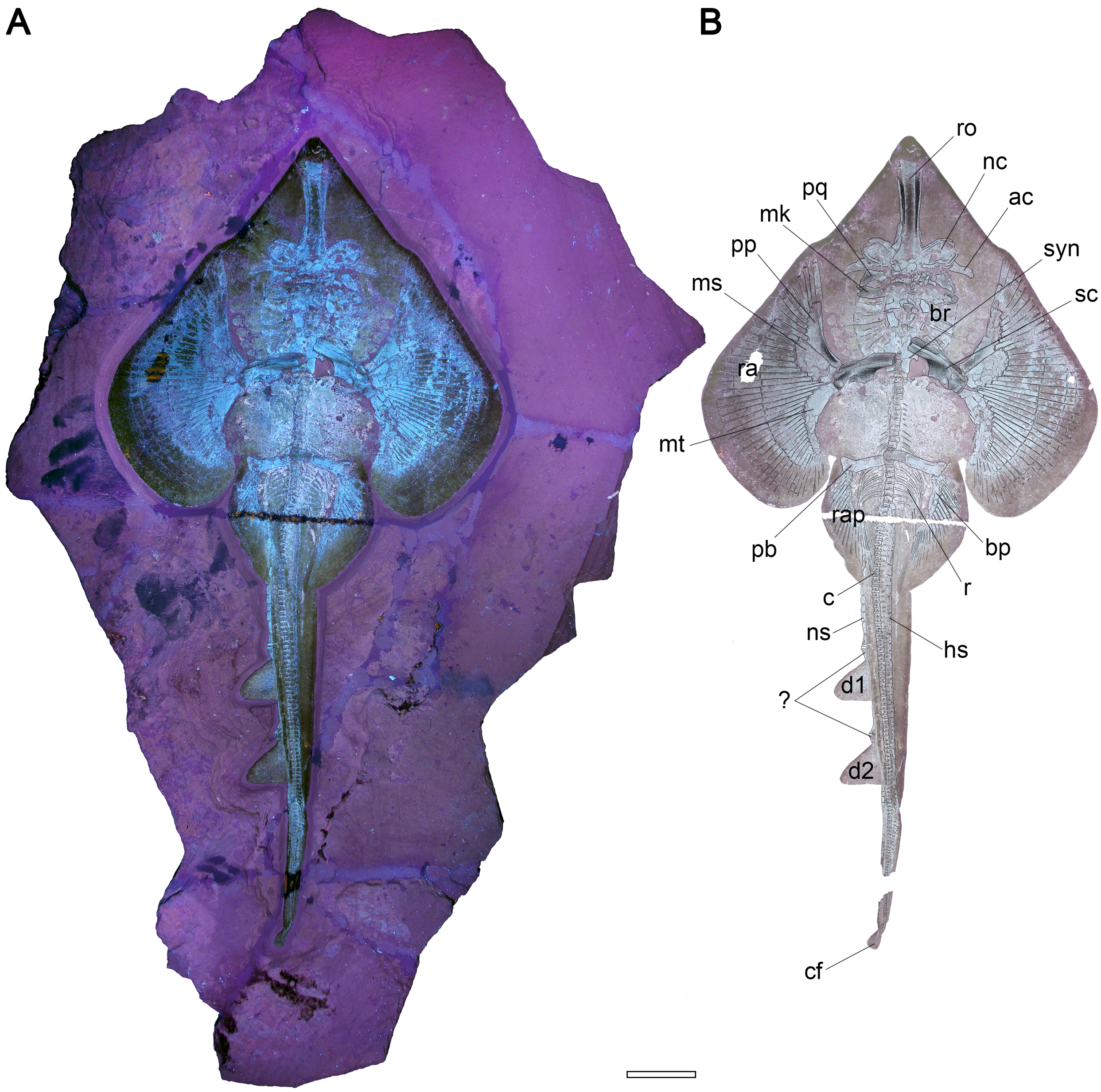

Figure S1. Overview of DMA-JP-2010/007 under ultraviolet light. A) photograph of the specimen. B) Illustration of the specimen showing the skeletal morphology. *Abbreviations*: **ac**, antorbital cartilage; **bp**, basipterygium; **br**, branchial arches; **c**, vertebral centra; **cf**, caudal fin; **d1**, first dorsal fin; **d2**, second dorsal fin; **hs**, hemal spine; **mk**, Meckel's cartilage; **ms**, mesopterygium; **mt**, metapterygium; **nc**, nasal capsule; **ns**, neural spine; **pb**, puboischiadic bar; **pp**, propterygium; **pq**, palatoquadarte; **r**, ribs; **ra**, pectoral fin radials; **rap**, pelvic fin radials; **ro**, rostrum; **sc**, scapulocoracoid; **syn**, synarcual. The scale bar equals 10 cm.

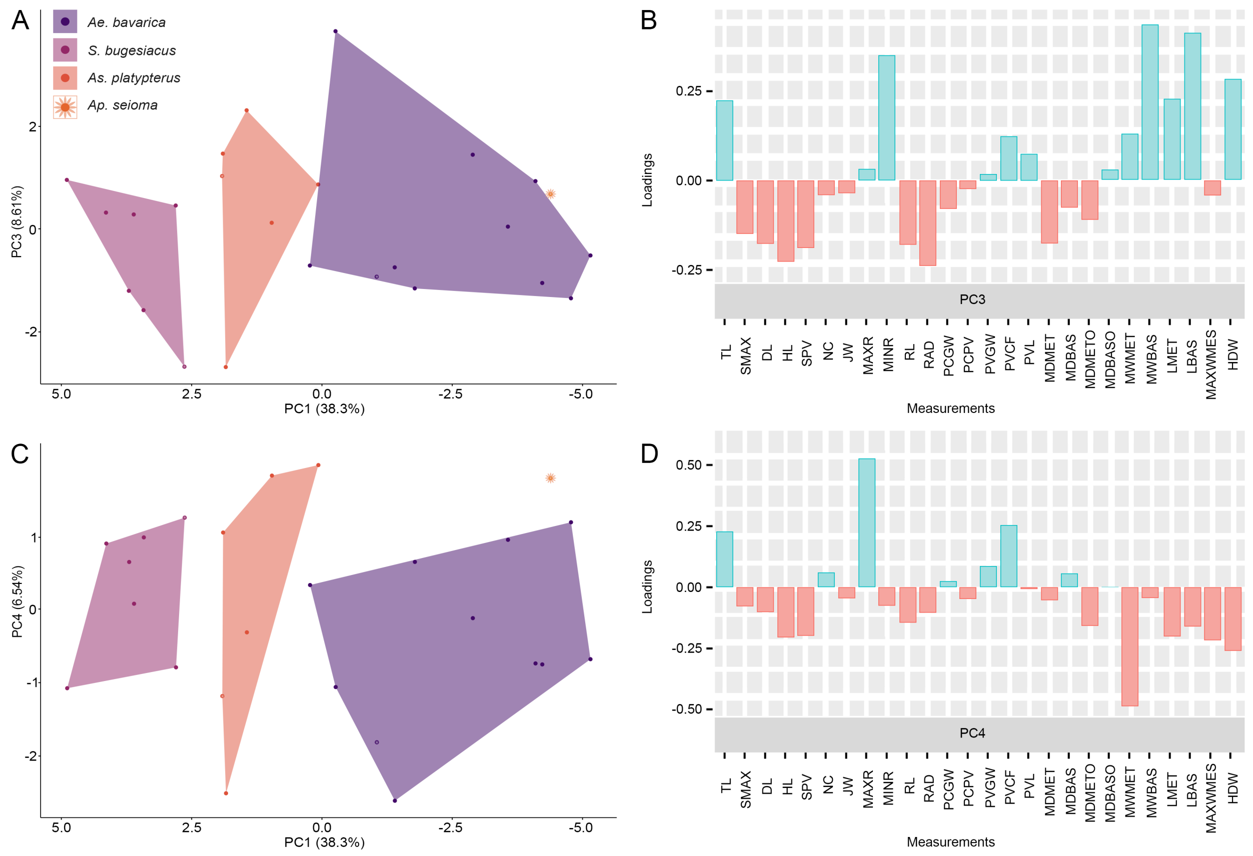

Figure S2. Traditional morphometrics: results of the principal component analysis (PCA), with each measurement adjusted to percentage of the disc width (DW) of each individual. A) morphospace plotted on PC1 (38.3% of the total variance) and PC3 (8.61%). Asterisks indicate the holotype of the respective species. B) loading values showing the variables associated with PC1 and PC3. C) morphospace plotted on PC1 (38.3% of the total variance) and PC4 (6.54%). Asterisks indicate the holotype of the respective species. D) loading values showing the variables associated with PC1 and PC4. *Abbreviations*: **DL**, disc length; **HDW**, half disc width; **HL**, head length; **JW**, jaw width; **LBAS**, length of basipterygia; **LMET**, length of metapterygia; **MAXR**, maximum rostrum width; **MAXWMES**, maximum width of mesopterygia; **MDBAS**, inner maximum distance between basipterygia; **MDBASO**, outer maximum distance between basipterygia; **MDMET**, inner maximum distance between metapterygia; **MDMETO**, outer maximum distance between metapterygia; **MINR**, minimum rostrum width; **MWBAS**, maximum width of basipterygia; **MWMET**, maximum width of metapterygia; **NC**, nasal capsules maximum width; **PCGW**, pectoral girdle width; **PCPV**, pectoral girdle to pelvic girdle; **PVCF**, pelvic girdle to caudal fin tip; **PVGW**, pelvic girdle width; **PVL**, pelvic fin length; **RAD**, span between anteriormost fin radials; **RL**, rostrum length; **SMAX**, distance from the tip of the snout to the point of maximum disc width; **SPV**, snout to pelvic girdle; **TL**, total length.

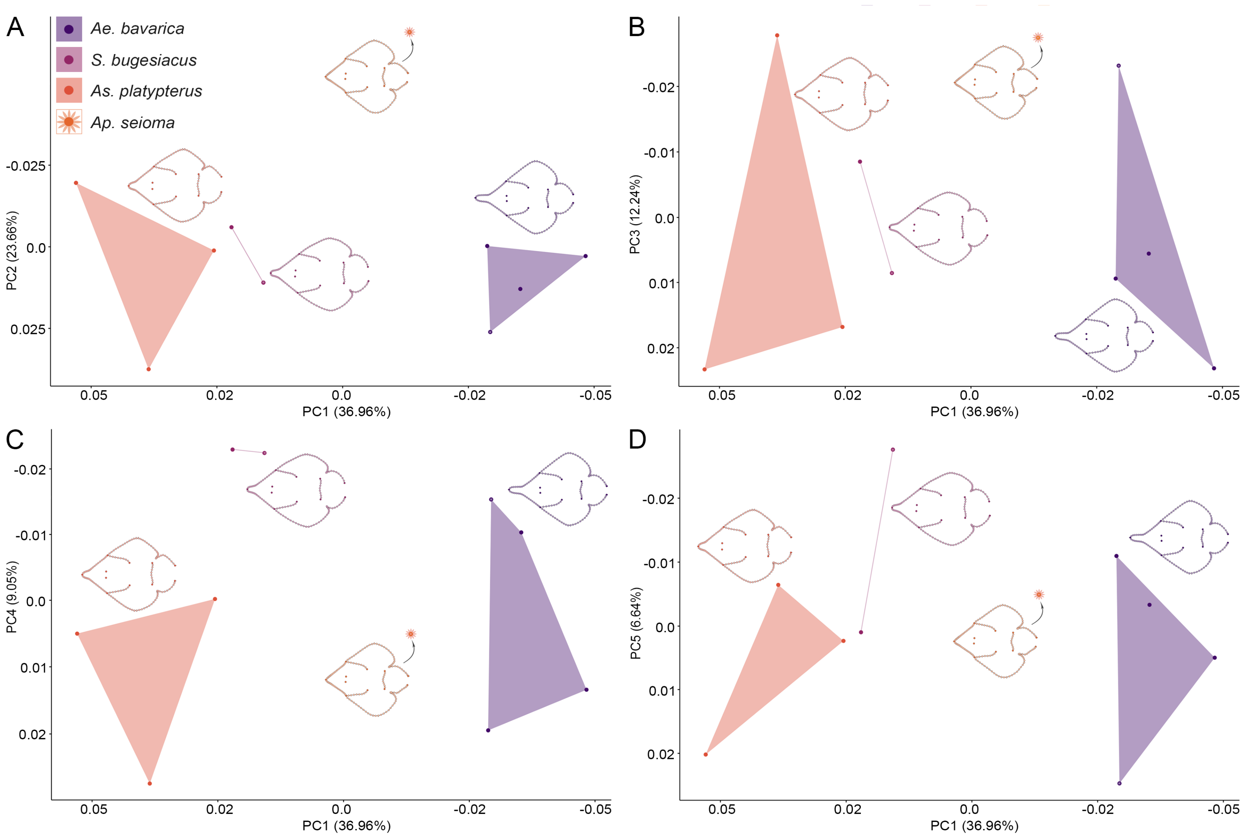

Figure S3. Geometric morphometrics – complete body: results of the principal component analysis (PCA). A) Morphospace plotted on PC1 (36.96%) and PC2 (23.66%). B) Morphospace plotted on PC1 (36.96%) and PC3 (12.24%). C) Morphospace plotted on PC1 (36.96%) and PC4 (9.05%). D) Morphospace plotted on PC1 (36.96%) and PC5 (6.64%). Asterisks indicate the holotype of the respective species. Mean shapes are shown next to each group. Dark-coloured dots of the mean shapes indicate true landmarks, light-coloured dots indicate semilandmarks.
