# Supplementary material for: Insights into stem Batomorphii: A new holomorphic ray (Chondrichthyes, Elasmobranchii) from the upper Jurassic of Germany
Source: PLoS One. 2025 Jan 23;20(1):e0310174. doi: 10.1371/journal.pone.0310174 (PMC11756912; doi:10.1371/journal.pone.0310174)
Supplement: S4 File — (PDF) [file pone.0310174.s004.pdf]

Supporting material 1 for:

Insights into stem Batomorphii: A new holomorphic ray (Chondrichthyes, Elasmobranchii) from the Upper Jurassic of Germany

JULIA TÜRTSCHER, PATRICK L. JAMBURA, FREDERIK SPINDLER, and JÜRGEN KRIWET

Traditional morphometrics (% DL)

Results

We performed a Principal Component Analysis considering all measurements that resulted in 24 axes (Table A), with the first four axes each explaining more than 5% of the variation and together accounting for 78.8% of the total variability (see Table B for loading values). The occupied morphospaces plotted on PC1 and PC2, PC1 and PC3, as well as PC1 and PC4 and the associated variables are shown in Figure S4. The comparison of the measured values between the groups in the form of boxplots is shown in Figure S5.

Along PC1 (45.96% of the variation), †*Ae. bavarica*, †*As. platypterus*, and †*Ap. seioma* gen. et sp. nov. are well separated, whereas †*S. bugesiacus* overlaps with †*Ap. seioma* gen. et sp. nov. and slightly with †*As. platypterus*. The occupied morphospaces of †*As. platypterus,* †*Ap. seioma* gen. et sp. nov. and †*S. bugesiacus* are in the positive range of PC1, whereas that of †*Ae. bavarica* is in the negative range. Along PC2 (13.51% of the variation), †*Ap. seioma* gen. et sp. nov. is clearly separated from all other taxa. On the other hand, †*Ae. bavarica*, †*As. platypterus*, and †*S. bugesiacus* overlap to a large extent. †*Apolithabatis seioma* gen. et sp. nov. lies in the positive region of PC2, †*Ae. bavarica* and †*As. platypterus* are located both in the positive and negative regions, and †*S. bugesiacus* is restricted to the negative area of PC2 (Figure S4A). Along PC3 (13.27% of the variation), †*Ap. seioma* gen. et sp. nov. overlaps with both †*Ae. bavarica* and †*S. bugesiacus,* but is separated from †*As. platypterus.* †*Apolithabatis seioma* gen. et sp. nov. is located in the positive area of PC3, while the morphospaces occupied by †*Ae. bavarica*, †*As. platypterus*, and †*S. bugesiacus* cover both the positive and the negative ranges of PC3 (Figure S4B). Along PC4 (6.07% of the variation), †*Ap. seioma* gen. et sp. nov. overlaps only with †*S. bugesiacus* but not with †*Ae. bavarica or* †*As. platypterus.* †*Apolithabatis seioma* gen. et sp. nov. is situated in the negative area of PC4, as is †*S.*

bugesiacus. †*Aellopobatis bavarica* and †*As. platypterus* are mainly located in the positive region of PC4, and to a lesser extent also in the negative region (Figure S4C). The associated variables for each of the first four PC axes are shown in Figure S4D (for loading values, see Table B).

The results of the Shapiro-Wilk normality test showed that *ca.* 76.92% of all measurements are normally distributed (i.e., 20 out of 26 measurements; Table C). The non-normally distributed measurements were further analyzed with the Kruskal–Wallis rank sum test, which indicates significant differences among the taxa for all but one measurement (i.e., maximum width of basipterygia; Table D), which is further supported by Wilcoxon pairwise comparisons between the taxa (Table E).

ANOVA tests on each normally distributed measurement showed significant differences in all but six measurements (i.e., total length, snout to maximum disc width, maximum rostrum width, minimum rostrum width, pelvic girdle to caudal fin tip, length of basipterygia; Table F), which was also largely confirmed by the Tukey test for pairwise comparisons (Table G).

Abbreviations

DW, disc width; **HDW**, half disc width; **HL**, head length; **JW**, jaw width; **LBAS**, length of basipterygia; **LMET**, length of metapterygia; **MAXR**, maximum rostrum width; **MAXWMES**, maximum width of mesopterygia; **MDBAS**, inner maximum distance between basipterygia; **MDBASO**, outer maximum distance between basipterygia; **MDMET**, inner maximum distance between metapterygia; **MDMETO**, outer maximum distance between metapterygia; **MINR**, minimum rostrum width; **MWBAS**, maximum width of basipterygia; **MWMET**, maximum width of metapterygia; **NC**, nasal capsules maximum width; **PCGW**, pectoral girdle width; **PCPV**, pectoral girdle to pelvic girdle; **PVCF**, pelvic girdle to caudal

fin tip; **PVGW**, pelvic girdle width; **PVL**, pelvic fin length; **RAD**, span between anteriormost fin radials; **RL**, rostrum length; **SMAX**, distance from the tip of the snout to the point of maximum disc width; **SPV**, snout to pelvic girdle; **TL**, total length.

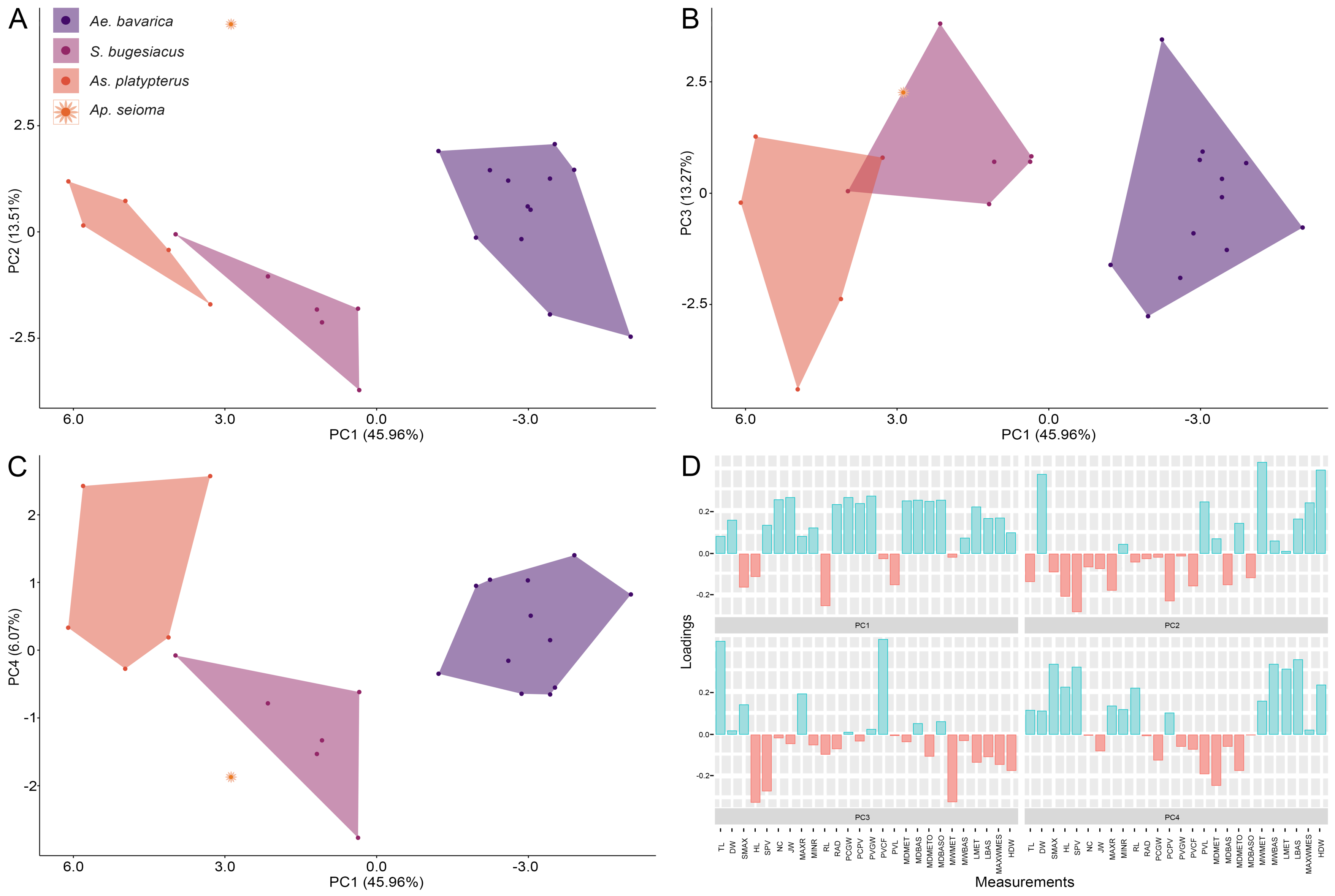

Figure S4. Traditional morphometrics: results of the principal component analysis (PCA), with each measurement adjusted to percentage of the disc length (DL) of each individual. A) morphospace plotted on PC1 (45.96% of the total variance) and PC2 (13.51%). B) morphospace plotted on PC1 and PC3 (13.27%). C) morphospace plotted on PC1 and PC4 (6.07%). D) loading values showing the variables associated with the first four PC axes.

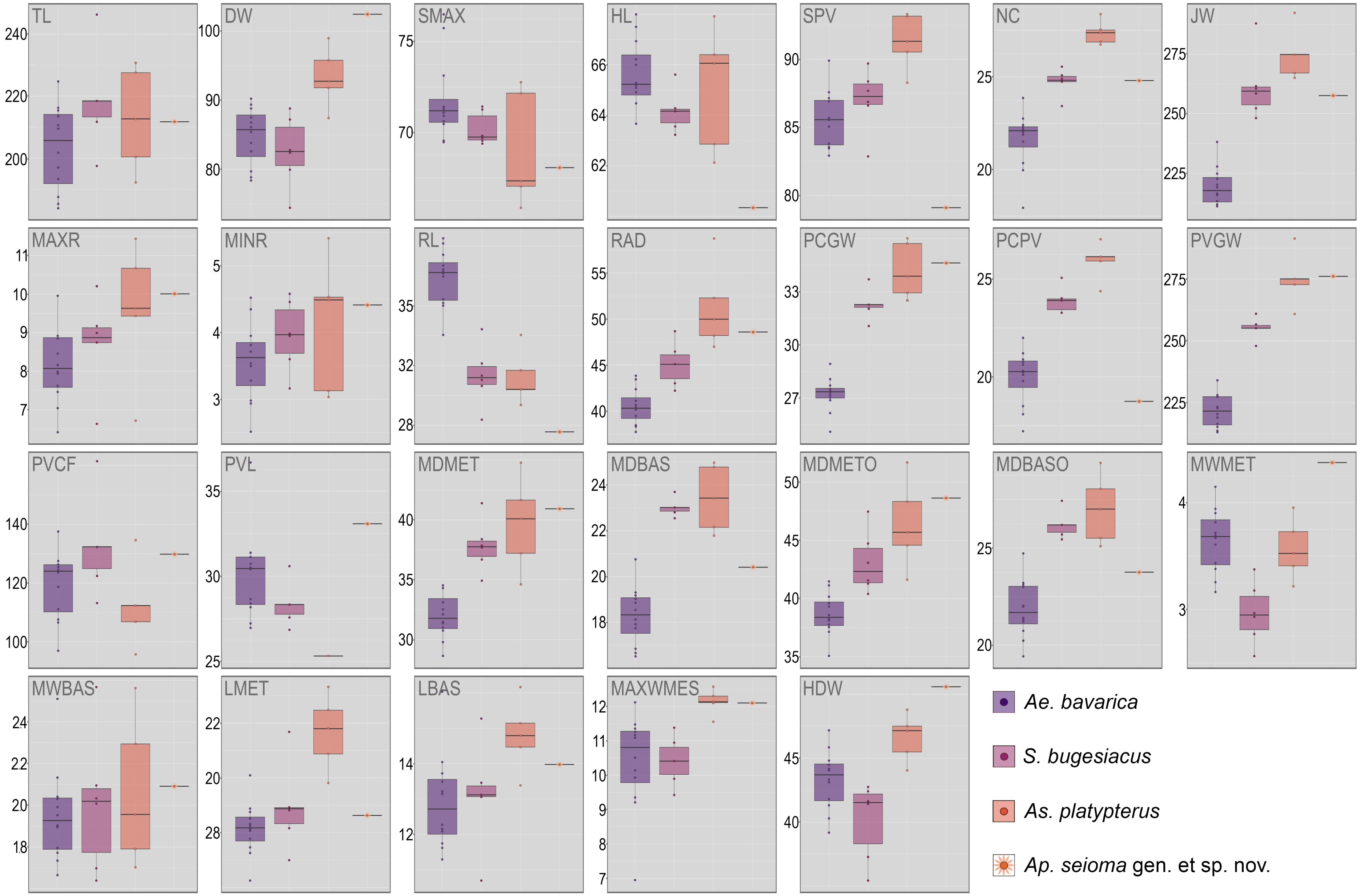

Figure S5. Traditional morphometrics: boxplots of all variables. Measurements are adjusted as percentage of the disc length (DL).

Table A. PC axes computed by all measurements; the first four axes each explain more than 5% of the total variation.

	PC1	PC2	PC3	PC4	PC5	PC6	PC7	PC8
Standard deviation	3.4567	1.8740	1.8576	1.25604	1.08984	0.99255	0.82740	0.78866
Proportion of Variance	0.4596	0.1351	0.1327	0.06068	0.04568	0.03789	0.02633	0.02392
Cumulative Proportion	0.4596	0.5946	0.7274	0.78804	0.83372	0.87161	0.89794	0.92186

	PC9	PC10	PC11	PC12	PC13	PC14	PC15	PC16
Standard deviation	0.7174	0.57657	0.53446	0.47653	0.42551	0.37795	0.3303	0.28628
Proportion of Variance	0.0198	0.01279	0.01099	0.00873	0.00696	0.00549	0.0042	0.00315
Cumulative Proportion	0.9417	0.95444	0.96543	0.97416	0.98113	0.98662	0.9908	0.99397

	PC17	PC18	PC19	PC20	PC21	PC22	PC23	PC24
Standard deviation	0.24902	0.18513	0.13970	0.13401	0.11761	0.08610	0.04230	7.009e-16
Proportion of Variance	0.00238	0.00132	0.00075	0.00069	0.00053	0.00029	0.00007	0.000e+00
Cumulative Proportion	0.99635	0.99767	0.99842	0.99911	0.99965	0.99993	1.00000	1.000e+00

Table B. Loadings of each measurement on the first four axes of the Principal Component Analysis (PCA) and the percentage (%var) explained by each one.

		PC1	PC2	PC3	PC4
Total length	**TL**	0.08398795	-0.13783901	0.45175585	0.117482944
Disc width	**DW**	0.16084508	0.38133797	0.01863041	0.114654208
Snout to maximum disc width	**SMAX**	-0.16697290	-0.09246794	0.14539770	0.341781855
Head length	**HL**	-0.11470848	-0.20997877	-0.32958952	0.229182090
Snout to pelvic girdle	**SPV**	0.13707502	-0.28398280	-0.27512740	0.327118638
Nasal capsules maximum width	**NC**	0.26098859	-0.06768543	-0.01974970	-0.004542563
Jaw width	**JW**	0.27131541	-0.07514088	-0.04600155	-0.082221672
Maximum rostrum width	**MAXR**	0.08475976	-0.18042771	0.19875386	0.138743778
Minimum rostrum width	**MINR**	0.12433235	0.04508997	0.08453069	0.121819082
Rostrum length	**RL**	-0.25420418	-0.04436846	-0.13285889	0.225862849
Span between last fin radials	**RAD**	0.23806412	-0.02686756	-0.15447252	-0.008051927
Pectoral girdle width	**PCGW**	0.27197013	-0.02170418	0.01347198	-0.124203254
Pectoral girdle to pelvic girdle	**PCPV**	0.24223323	-0.23216675	-0.06382291	0.106263159
Pelvic girdle width	**PVGW**	0.27774900	-0.01656618	0.02535440	-0.059335268
Pelvic girdle to caudal fin tip	**PVCF**	-0.02833856	-0.16001530	0.46104213	-0.071829090
Pelvic fin length	**PVL**	-0.15451636	0.24963242	0.25208563	-0.191860780
Inner maximum distance between metapterygia	**MDMET**	0.25384396	0.07181507	-0.02081050	-0.249728410
Inner maximum distance between basipterygia	**MDBAS**	0.25842798	-0.15243325	0.05411038	-0.059337216
Outer maximum distance between metapterygia	**MDMETO**	0.25307059	0.14517873	-0.02875885	-0.176612006
Outer maximum distance between basipterygia	**MDBASO**	0.25977770	-0.11983080	0.06378366	-0.005216477
Maximum width of metapterygia	**MWMET**	-0.02102575	0.44105306	-0.14941790	0.161877234
Maximum width of basipterygia	**MWBAS**	0.07700910	0.06259335	0.37471106	0.342966075
Length of metapterygia	**LMET**	0.22323440	0.01146952	-0.08164571	0.318673739
Length of basipterygia	**LBAS**	0.16935356	0.16628620	0.13652082	0.362909066
Maximum width of mesopterygium	**MAXWMES**	0.17098223	0.24425671	-0.14527749	0.022172613
Half disc width	**HDW**	0.10120789	0.40311477	0.03212499	0.240553503
	**%var**	45.96%	13.51%	13.27%	6.07%

Table C. Shapiro-Wilk normality test; bold indicates a *p*-value <0.05 hence suggesting non-normal distribution.

		**W**	***p***
Total length	TL	0.96753	0.6066
Disc width	DW	0.97586	0.8091
Snout to maximum disc width	SMAX	0.95216	0.3016
Head length	HL	0.9782	0.8605
Snout to pelvic girdle	SPV	0.97981	0.8923
Nasal capsules maximum width	NC	0.97425	0.7714
Jaw width	JW	0.90359	**0.02566**
Maximum rostrum width	MAXR	0.98101	0.9136
Minimum rostrum width	MINR	0.96999	0.6668
Rostrum length	RL	0.93698	0.1397
Span between last fin radials	RAD	0.92199	0.06465
Pectoral girdle width	PCGW	0.90737	**0.03093**
Pectoral girdle to pelvic girdle	PCPV	0.95656	0.3733
Pelvic girdle width	PVGW	0.90686	**0.03016**
Pelvic girdle to caudal fin tip	PVCF	0.9505	0.2777
Pelvic fin length	PVL	0.91712	0.05045
Inner maximum distance between metapterygia	MDMET	0.96404	0.5248
Inner maximum distance between basipterygia	MDBAS	0.93148	0.1052
Outer maximum distance between metapterygia	MDMETO	0.94401	0.2003
Outer maximum distance between basipterygia	MDBASO	0.96911	0.645
Maximum width of metapterygia	MWMET	0.99158	0.9987
Maximum width of basipterygia	MWBAS	0.90545	**0.02812**
Length of metapterygia	LMET	0.91401	**0.04313**
Length of basipterygia	LBAS	0.97626	0.8183
Maximum width of mesopterygium	MAXWMES	0.91559	**0.04671**
Half discwidth	HDW	0.98737	0.986

Table D. Kruskal-Wallis rank sum test on non-normally distributed measurements; bold indicates a *p*-value <0.05.

		**df**	**$\chi 2$**	***p***
Jaw width	JW	3	18.595	**0.0003314**
Pectoral girdle width	PCGW	3	19.036	**0.0002687**
Pelvic girdle width	PVGW	3	19.536	**0.0002117**
Maximum width of basipterygia	MWBAS	3	0.891	0.8276
Length of metapterygia	LMET	3	11.643	**0.008713**
Maximum width of mesopterygium	MAXWMES	3	11.786	**0.008153**

Table E. Post hoc pairwise Wilcoxon test with non-normally distributed measurements; bold indicates a *p*-value <0.05.

**JW**	*Aellopobatis*	*Apolithabatis*	*Asterodermus*
Apolithabatis	0.92308	-	-
Asterodermus	**0.01117**	1.00000	-
Spathobatis	**0.00065**	1.00000	0.32809

**PCGW**	*Aellopobatis*	*Apolithabatis*	*Asterodermus*
Apolithabatis	0.9231	-	-
Asterodermus	**0.0019**	1.00000	-
Spathobatis	**0.0052**	1.00000	0.1276

**PVGW**	*Aellopobatis*	*Apolithabatis*	*Asterodermus*
Apolithabatis	0.9231	-	-
Asterodermus	**0.0112**	1.00000	-
Spathobatis	**0.0052**	1.00000	**0.0444**

**MWBAS**	*Aellopobatis*	*Apolithabatis*	*Asterodermus*
Apolithabatis	1.00000	-	-
Asterodermus	1.00000	1.00000	-
Spathobatis	1.00000	1.00000	1.00000

**LMET**	*Aellopobatis*	*Apolithabatis*	*Asterodermus*
Apolithabatis	1.00000	-	-
Asterodermus	**0.0039**	1.00000	-
Spathobatis	1.00000	1.00000	0.1330

**MAXWMES**	*Aellopobatis*	*Apolithabatis*	*Asterodermus*
Apolithabatis	1.00000	-	-
Asterodermus	**0.0078**	1.00000	-
Spathobatis	1.00000	1.00000	**0.0478**

Table F. Results of the ANOVA to test for differences in the normally distributed measurements between the taxa; bold indicates a *p*-value <0.05.

**TL**	**Df**	**SS**	**MS**	**Rsq**	**F**	**Z**	**Pr(>F)**
Taxa	3	999.8	333.26	0.18437	1.507	0.69619	0.2396
Residuals	20	4422.9	221.15	0.81563			
Total	23	5422.7					

**DW**	**Df**	**SS**	**MS**	**Rsq**	**F**	**Z**	**Pr(>F)**
Taxa	3	614.61	204.869	0.60734	10.312	3.5163	**0.0002**
Residuals	20	397.35	19.868	0.39266			
Total	23	1011.96					

**SMAX**	**Df**	**SS**	**MS**	**Rsq**	**F**	**Z**	**Pr(>F)**
Taxa	3	36.688	12.2292	0.26796	2.4403	1.242	0.1167
Residuals	20	100.226	5.0113	0.73204			
Total	23	136.914					

**HL**	**Df**	**SS**	**MS**	**Rsq**	**F**	**Z**	**Pr(>F)**
Taxa	3	30.795	10.2650	0.39719	4.3926	2.0863	**0.0179**
Residuals	20	46.738	2.3369	0.60281			
Total	23	77.533					

**SPV**	**Df**	**SS**	**MS**	**Rsq**	**F**	**Z**	**Pr(>F)**
Taxa	3	179.191	59.730	0.66492	13.229	4.1043	**0.0001**
Residuals	20	90.303	4.515	0.33508			
Total	23	269.495					

**NC**	**Df**	**SS**	**MS**	**Rsq**	**F**	**Z**	**Pr(>F)**
Taxa	3	127.277	42.426	0.80812	28.077	5.6903	**0.0001**
Residuals	20	30.221	1.511	0.19188			
Total	23	157.497					

**MAXR**	**Df**	**SS**	**MS**	**Rsq**	**F**	**Z**	**Pr(>F)**
Taxa	3	9.268	3.0893	0.23721	2.0731	1.1222	0.1276
Residuals	20	29.803	1.4902	0.76279			
Total	23	39.071					

MINR	Df	SS	MS	Rsq	F	Z	Pr(>F)
Taxa	3	1.671	0.55700	0.15336	1.2076	0.42843	0.326
Residuals	20	9.225	0.46125	0.84664			
Total	23	10.896					

RL	Df	SS	MS	Rsq	F	Z	Pr(>F)
Taxa	3	299.54	99.845	0.8075	27.965	6.3903	**0.0001**
Residuals	20	71.41	3.570	0.1925			
Total	23	370.94					

RAD	Df	SS	MS	Rsq	F	Z	Pr(>F)
Taxa	3	436.18	145.392	0.73662	18.645	4.0079	**0.0002**
Residuals	20	155.96	7.798	0.26338			
Total	23	592.13					

PCPV	Df	SS	MS	Rsq	F	Z	Pr(>F)
Taxa	3	157.765	52.588	0.8543	39.091	6.7775	**0.0001**
Residuals	20	26.906	1.345	0.1457			
Total	23	184.671					

PVCF	Df	SS	MS	Rsq	F	Z	Pr(>F)
Taxa	3	1249.0	416.35	0.25958	2.3372	1.1972	0.1151
Residuals	20	3562.8	178.14	0.74042			
Total	23	4811.9					

PVL	Df	SS	MS	Rsq	F	Z	Pr(>F)
Taxa	3	100.849	33.616	0.55133	8.192	2.9262	**0.0015**
Residuals	20	82.071	4.104	0.44867			
Total	23	182.920					

MDMET	Df	SS	MS	Rsq	F	Z	Pr(>F)
Taxa	3	294.52	98.175	0.70409	15.862	4.6158	**0.0001**
Residuals	20	123.78	6.189	0.29591			
Total	23	418.31					

MDBAS	Df	SS	MS	Rsq	F	Z	Pr(>F)
Taxa	3	139.165	46.388	0.84052	35.137	6.5806	**0.0001**
Residuals	20	26.404	1.320	0.15948			
Total	23	165.570					

**MDMETO**	**Df**	**SS**	**MS**	**Rsq**	**F**	**Z**	**Pr(>F)**
Taxa	3	279.14	93.048	0.68553	14.533	4.3455	**0.0001**
Residuals	20	128.05	6.402	0.31447			
Total	23	407.19					

**MDBASO**	**Df**	**SS**	**MS**	**Rsq**	**F**	**Z**	**Pr(>F)**
Taxa	3	129.169	43.056	0.76955	22.262	5.3291	**0.0001**
Residuals	20	38.682	1.934	0.23045			
Total	23	167.851					

**MWMET**	**Df**	**SS**	**MS**	**Rsq**	**F**	**Z**	**Pr(>F)**
Taxa	3	2.7554	0.91847	0.62269	11.002	3.6759	**0.0003**
Residuals	20	1.6696	0.08348	0.37731			
Total	23	4.4250					

**LBAS**	**Df**	**SS**	**MS**	**Rsq**	**F**	**Z**	**Pr(>F)**
Taxa	3	13.449	4.4829	0.28264	2.6267	1.4372	0.0704
Residuals	20	34.133	1.7067	0.71736			
Total	23	47.582					

**HDW**	**Df**	**SS**	**MS**	**Rsq**	**F**	**Z**	**Pr(>F)**
Taxa	3	165.85	55.282	0.5824	9.2977	3.3403	**0.0002**
Residuals	20	118.92	5.946	0.4176			
Total	23	284.76					

Table G. Results of the Tukey's Honest Significant Difference test to test for differences in the normally distributed measurements between the taxa; bold indicates a *p*-value <0.05.

**TL**	**diff**	**lwr**	**upr**	**p adj**
Apolithabatis : Aellopobatis	8.4741133	-34.848315	51.79654	0.9461489
Asterodermus : Aellopobatis	9.4004437	-12.755006	31.55589	0.6413818
Spathobatis : Aellopobatis	15.1661458	-5.645276	35.97757	0.2071474
Asterodermus : Apolithabatis	0.9263304	-44.669210	46.52187	0.9999316
Spathobatis : Apolithabatis	6.6920325	-38.265777	51.64984	0.9749860
Spathobatis : Asterodermus	5.7657021	-19.438181	30.96959	0.9176734

**DW**	**diff**	**lwr**	**upr**	**p adj**
Apolithabatis : Aellopobatis	17.665027	4.679887	30.650168	**0.0055890**
Asterodermus : Aellopobatis	8.568994	1.928285	15.209703	**0.0086812**
Spathobatis : Aellopobatis	-2.192693	-8.430552	4.045167	0.7600965
Asterodermus : Apolithabatis	-9.096034	-22.762500	4.570432	0.2749549
Spathobatis : Apolithabatis	-19.857720	-33.333037	-6.382403	**0.0027230**
Spathobatis : Asterodermus	-10.761686	-18.316110	-3.207263	**0.0037218**

**SMAX**	**diff**	**lwr**	**upr**	**p adj**
Apolithabatis : Aellopobatis	-3.7306018	-10.252131	2.7909276	0.4004466
Asterodermus : Aellopobatis	-2.7595322	-6.094697	0.5756321	0.1278302
Spathobatis : Aellopobatis	-1.5999169	-4.732758	1.5329245	0.4965603
Asterodermus : Apolithabatis	0.9710695	-5.892642	7.8347811	0.9783694
Spathobatis : Apolithabatis	2.1306849	-4.637026	8.8983957	0.8145174
Spathobatis : Asterodermus	1.1596154	-2.634444	4.9536747	0.8273161

**HL**	**diff**	**lwr**	**upr**	**p adj**
Apolithabatis : Aellopobatis	-5.3022055	-9.75561449	-0.8487966	**0.0161087**
Asterodermus : Aellopobatis	-0.5682330	-2.84574338	1.7092773	0.8965093
Spathobatis : Aellopobatis	-1.4799559	-3.61930439	0.6593925	0.2451325
Asterodermus : Apolithabatis	4.7339725	0.04689488	9.4210501	**0.0472128**
Spathobatis : Apolithabatis	3.8222496	-0.79927127	8.4437705	0.1280598
Spathobatis : Asterodermus	-0.9117229	-3.50260255	1.6791567	0.7595028

SPV	diff	lwr	upr	p adj
Apolithabatis : Aellopobatis	-6.489840	-12.680129	-0.2995507	**0.0377939**
Asterodermus : Aellopobatis	5.730676	2.564911	8.8964419	**0.0003188**
Spathobatis : Aellopobatis	1.419954	-1.553765	4.3936725	0.5515639
Asterodermus : Apolithabatis	12.220516	5.705425	18.7356079	**0.0002108**
Spathobatis : Apolithabatis	7.909794	1.485827	14.3337604	**0.0125380**
Spathobatis : Asterodermus	-4.310723	-7.912075	-0.7093705	**0.0154907**

NC	diff	lwr	upr	p adj
Apolithabatis : Aellopobatis	3.18370743	-0.3973463	6.7647611	0.0925390
Asterodermus : Aellopobatis	5.75917135	3.9277907	7.5905520	**0.0000001**
Spathobatis : Aellopobatis	3.11965844	1.3993759	4.8399410	**0.0003123**
Asterodermus : Apolithabatis	2.57546392	-1.1934863	6.3444142	0.2544760
Spathobatis : Apolithabatis	-0.06404899	-3.7802840	3.6521861	0.9999582
Spathobatis : Asterodermus	-2.63951291	-4.7228785	-0.5561473	**0.0100484**

MAXR	diff	lwr	upr	p adj
Apolithabatis : Aellopobatis	1.8635298	-1.6926904	5.419750	0.4749638
Asterodermus : Aellopobatis	1.4340034	-0.3846771	3.252684	0.1554848
Spathobatis : Aellopobatis	0.6038761	-1.1044769	2.312229	0.7570403
Asterodermus : Apolithabatis	-0.4295264	-4.1723402	3.313287	0.9881904
Spathobatis : Apolithabatis	-1.2596538	-4.9501179	2.430810	0.7756959
Spathobatis : Asterodermus	-0.8301274	-2.8990455	1.238791	0.6799859

MINR	diff	lwr	upr	p adj
Apolithabatis : Aellopobatis	0.8453565	-1.1331673	2.8238803	0.6363724
Asterodermus : Aellopobatis	0.5505075	-0.4613260	1.5623411	0.4431677
Spathobatis : Aellopobatis	0.3885856	-0.5618665	1.3390377	0.6673166
Asterodermus : Apolithabatis	-0.2948489	-2.3771851	1.7874873	0.9783183
Spathobatis : Apolithabatis	-0.4567709	-2.5099821	1.5964403	0.9235941
Spathobatis : Asterodermus	-0.1619219	-1.3129766	0.9891327	0.9787211

RL	diff	lwr	upr	p adj
Apolithabatis : Aellopobatis	-10.3010389	-15.805660	-4.796418	**0.0002167**
Asterodermus : Aellopobatis	-6.6889732	-9.504082	-3.873864	**0.0000100**
Spathobatis : Aellopobatis	-6.5407175	-9.185052	-3.896383	**0.0000057**
Asterodermus : Apolithabatis	3.6120656	-2.181381	9.405512	0.3278699
Spathobatis : Apolithabatis	3.7603214	-1.952094	9.472737	0.2836930
Spathobatis : Asterodermus	0.1482557	-3.054192	3.350704	0.9991956

RAD	diff	lwr	upr	p adj
Apolithabatis : Aellopobatis	8.083316	-0.0517354	16.218367	0.0518491
Asterodermus : Aellopobatis	10.745191	6.5848581	14.905524	**0.0000030**
Spathobatis : Aellopobatis	4.567495	0.6595426	8.475447	**0.0184065**
Asterodermus : Apolithabatis	2.661875	-5.9000194	11.223769	0.8200394
Spathobatis : Apolithabatis	-3.515821	-11.9579628	4.926321	0.6545583
Spathobatis : Asterodermus	-6.177696	-10.9104613	-1.444931	**0.0079062**

PCPV	diff	lwr	upr	p adj
Apolithabatis : Aellopobatis	-1.229045	-4.607997	2.14990647	0.7409587
Asterodermus : Aellopobatis	5.942918	4.214894	7.67094146	**0.0000000**
Spathobatis : Aellopobatis	3.927387	2.304192	5.55058331	**0.0000077**
Asterodermus : Apolithabatis	7.171963	3.615719	10.72820664	**0.0000874**
Spathobatis : Apolithabatis	5.156433	1.649929	8.66293646	**0.0027774**
Spathobatis : Asterodermus	-2.015530	-3.981318	-0.04974224	**0.0432257**

PVCF	diff	lwr	upr	p adj
Apolithabatis : Aellopobatis	10.482517	-28.400216	49.36525	0.8736456
Asterodermus : Aellopobatis	-6.987303	-26.872256	12.89765	0.7602932
Spathobatis : Aellopobatis	13.004008	-5.674653	31.68267	0.2403430
Asterodermus : Apolithabatis	-17.469820	-58.392716	23.45308	0.6369969
Spathobatis : Apolithabatis	2.521491	-37.829029	42.87201	0.9980332
Spathobatis : Asterodermus	19.991311	-2.629671	42.61229	0.0951965

PVL	diff	lwr	upr	p adj
Apolithabatis : Aellopobatis	2.963374	-2.9380050	8.864752	0.5105685
Asterodermus : Aellopobatis	-4.794626	-7.8126404	-1.776612	**0.0013062**
Spathobatis : Aellopobatis	-1.772372	-4.6073027	1.062559	0.3255944
Asterodermus : Apolithabatis	-7.758000	-13.9690218	-1.546978	**0.0112301**
Spathobatis : Apolithabatis	-4.735746	-10.8598959	1.388404	0.1675281
Spathobatis : Asterodermus	3.022254	-0.4110173	6.455526	0.0969734

MDMET	diff	lwr	upr	p adj
Apolithabatis : Aellopobatis	8.914333	1.666822	16.161844	**0.0126399**
Asterodermus : Aellopobatis	7.661049	3.954612	11.367486	**0.0000641**
Spathobatis : Aellopobatis	5.802123	2.320531	9.283715	**0.0007945**
Asterodermus : Apolithabatis	-1.253283	-8.881069	6.374502	0.9668898
Spathobatis : Apolithabatis	-3.112210	-10.633307	4.408888	0.6590260
Spathobatis : Asterodermus	-1.858926	-6.075343	2.357491	0.6133719

MDBAS	diff	lwr	upr	p adj
Apolithabatis : Aellopobatis	2.1178248	-1.2294956	5.465145	0.3157640
Asterodermus : Aellopobatis	5.1397313	3.4278839	6.851579	**0.0000003**
Spathobatis : Aellopobatis	4.7280342	3.1200335	6.336035	**0.0000004**
Asterodermus : Apolithabatis	3.0219065	-0.5010465	6.544860	0.1092227
Spathobatis : Apolithabatis	2.6102094	-0.8634691	6.083888	0.1861532
Spathobatis : Asterodermus	-0.4116972	-2.3590827	1.535688	0.9334111

MDMETO	diff	lwr	upr	p adj
Apolithabatis : Aellopobatis	9.965729	2.5944169	17.3370401	**0.0058950**
Asterodermus : Aellopobatis	7.709738	3.9399876	11.4794876	**0.0000733**
Spathobatis : Aellopobatis	4.419917	0.8788536	7.9609806	**0.0112917**
Asterodermus : Apolithabatis	-2.255991	-10.0140724	5.5020906	0.8471775
Spathobatis : Apolithabatis	-5.545811	-13.1953830	2.1037601	0.2107940
Spathobatis : Asterodermus	-3.289821	-7.5782614	0.9986204	0.1726132

MDBASO	diff	lwr	upr	p adj
Apolithabatis : Aellopobatis	1.9027270	-2.148730	5.954184	0.5647740
Asterodermus : Aellopobatis	5.1528738	3.080925	7.224823	**0.0000052**
Spathobatis : Aellopobatis	4.3384321	2.392175	6.284689	**0.0000239**
Asterodermus : Apolithabatis	3.2501468	-1.013889	7.514182	0.1767611
Spathobatis : Apolithabatis	2.4357050	-1.768691	6.640101	0.3896815
Spathobatis : Asterodermus	-0.8144417	-3.171476	1.542593	0.7692904

MWMET	diff	lwr	upr	p adj
Apolithabatis : Aellopobatis	0.7302599	-0.1114614	1.57198130	0.1037332
Asterodermus : Aellopobatis	-0.0777850	-0.5082483	0.35267829	0.9567635
Spathobatis : Aellopobatis	-0.6799786	-1.0843285	-0.27562873	**0.0007214**
Asterodermus : Apolithabatis	-0.8080449	-1.6939311	0.07784121	0.0816864
Spathobatis : Apolithabatis	-1.4102385	-2.2837341	-0.53674300	**0.0011077**
Spathobatis : Asterodermus	-0.6021936	-1.0918856	-0.11250159	**0.0126591**

LBAS	diff	lwr	upr	p adj
Apolithabatis : Aellopobatis	1.0742345	-2.73159308	4.8800620	0.8581075
Asterodermus : Aellopobatis	1.8881193	-0.05821253	3.8344511	0.0592514
Spathobatis : Aellopobatis	0.2153963	-1.61286410	2.0436567	0.9872522
Asterodermus : Apolithabatis	0.8138848	-3.19163304	4.8194027	0.9402262
Spathobatis : Apolithabatis	-0.8588382	-4.80833205	3.0906557	0.9281357
Spathobatis : Asterodermus	-1.6727230	-3.88685631	0.5414103	0.1825856

HDW	diff	lwr	upr	p adj
Apolithabatis : Aellopobatis	7.293936	0.1903623	14.3975099	**0.0428534**
Asterodermus : Aellopobatis	3.299652	-0.3331744	6.9324790	0.0834115
Spathobatis : Aellopobatis	-3.141737	-6.5541837	0.2707093	0.0779668
Asterodermus : Apolithabatis	-3.994284	-11.4705795	3.4820119	0.4586128
Spathobatis : Apolithabatis	-10.435673	-17.8074003	-3.0639464	**0.0039388**
Spathobatis : Asterodermus	-6.441390	-10.5740675	-2.3087116	**0.0015825**
