# Supplementary material for: Insights into stem Batomorphii: A new holomorphic ray (Chondrichthyes, Elasmobranchii) from the upper Jurassic of Germany
Source: PLoS One. 2025 Jan 23;20(1):e0310174. doi: 10.1371/journal.pone.0310174 (PMC11756912; doi:10.1371/journal.pone.0310174)
Supplement: S5 File — (DOCX) [file pone.0310174.s005.docx]

**Character list:**

Characters have been assembled from previously published data matrices. Octothorpes (#) before numbers are used to abbreviate 'character(s)'.

**Neurocranium (Skeleton)**

1. Sup: Rostral cartilages: [0] arise from the medial area of the trabecula only, [1] medial area of the trabecula + lamina orbitonasalis.
   (#1 Jambura *et al.*, 2023; #3 Villalobos-Segura *et al.*, 2022).
2. Sub: Rostral cartilage: [0] well-developed rostral plate with various degrees of contribution from the lamina orbitonasalis, [1] reaches the tip of the snout (carried by the growth of the pectoral fin), [2] reaches the tip of the snout (growth of lamina orbitonasalis to support the cephalic fins).
   (#2 Jambura *et al.*, 2023; #4 Villalobos-Segura *et al.*, 2022).
3. Medial growth of rostral cartilage: [0] inconspicuous, [1] conspicuous (noticeable).
   (#5 in Villalobos-Segura *et al.*, 2022; #27 Villalobos-Segura *et al.*, 2019; #1 Claeson *et al.*, 2013; #26 Aschliman *et al.*, 2012, #1 Brito & Dutheil, 2004)
4. Different types of cartilage on rostrum: [0] absent, [1] present.
   (#6 Villalobos-Segura *et al.*, 2022)
5. Sup: Rostral processes: [0] absent, [1] present.
   (#5 Jambura *et al.*, 2023; #7 Villalobos-Segura *et al.*, 2022; #30 Marramà *et al.*, 2020; #32 Villalobos-Segura *et al.*, 2019; #29 Aschliman *et al.* 2012).
6. Sub: Rostral processes (proximal articulation): [0] articulated with nasal capsules, [1] continuous with chondrocranium, [2] articulated with ventral aspect of rostral cartilage.
   (#6 Jambura *et al.*, 2023; #8 Villalobos-Segura *et al.*, 2022).
7. Rostral appendix: [0] absent, [1] present.
   (#7 Jambura *et al.*, 2023; #9 Villalobos-Segura *et al.*, 2022; #28 Marramà *et al.*, 2020; #30 Villalobos-Segura *et al.*, 2019; #3 Claeson *et al.*, 2013; #28 Aschliman *et al.*, 2012; #25 McEachran & Aschliman, 2004; #21 McEachran *et al.*, 1996).
8. Downturned ethmoidal region: [0] absent, [1] present.
   (#18 Villalobos-Segura *et al.*, 2022; #3(3) Klug, 2010; #4 de Carvalho, 1996).
9. Caudal internasal keel: [0] absent, [1] present.
   (#9 Jambura *et al.*, 2023; #10 Villalobos-Segura *et al.*, 2022).
10. Rostral passage of superficial ophthalmic nerve: [0] covered, [1] open.
    (#10 Jambura *et al.*, 2023; #11 Villalobos-Segura *et al.*, 2022).
11. Precerebral fontanelle: [0] absent, [1] present.
    (#12 Jambura *et al.*, 2023; #142 Villalobos-Segura *et al.*, 2022, #23 Landemaine *et al.*, 2018; #23(17) Klug, 2010; #4 de Carvalho & Maisey, 1996; #13 Shirai, 1992; #62 Coates *et al.*, 2017).
12. Nasal capsules: [0] laterally expanded, [1] ventro-laterally expanded, [2] anteriorly expanded, [3] trumpet-like.
    (#15 Jambura *et al.*, 2023; #34 Villalobos-Segura *et al.*, 2022; #32 Marramà *et al.*, 2020; #34 Villalobos-Segura *et al.*, 2019; #44 Landemaine *et al.*, 2018; #10 Claeson *et al.*, 2013; #31 Aschliman *et al.*, 2012; #27 McEachran & Aschliman, 2004; #44(32) Klug, 2010; #23 McEachran *et al.*, 1996; #4 Shirai, 1996; #37 Nishida, 1990).
13. Nasal capsule margin: [0] straight, [1] horn-like process.
    (#16 Jambura *et al.*, 2023; #35 Villalobos-Segura *et al.*, 2022; #79 Marramà *et al.*, 2020; #83 Villalobos-Segura *et al.*, 2019; #9 Claeson *et al.*, 2013; #5 Brito & Dutheil, 2004).
14. Anterior preorbital foramen: [0] located dorsally, [1] located anteriorly.
    (#17 Jambura *et al.*, 2023; #12 Villalobos-Segura *et al.*, 2022; #35 Marramà *et al.*, 2020; #37 Villalobos-Segura *et al.*, 2019; #35 Aschliman *et al.*, 2012; #27 McEachran *et al.*, 1996; #85 Nishida, 1990).

According to Nishida (1990) and subsequent works (McEachran *et al.*, 1996; Aschliman *et al.*, 2012), the anterior preorbital foramen is situated dorsally in most batomorphs except for the pelagic Myliobatiformes, in which it is situated anteriorly. According to Villalobos-Segura *et al.* (2022), the anterior opening of the preorbital foramen is a synapomorphy for pelagic Myliobatiformes and independently evolved in Rhinopristiformes and †*Spathobatis*. Although an anterior opening of the preorbital foramen is indicated in one †*Spathobatis bugesiacus* specimen (MCZ 317) reexamined here, we cannot confidently confirm this observation. Therefore, we scored this character as unknown ('?').

1. Preorbital process (nasal capsule): [0] present, [1] absent.
   (#18 Jambura *et al.*, 2023; #13 Villalobos-Segura *et al.*, 2022; #33 Marramà et al., 2020; #35 Villalobos-Segura *et al.*, 2019; #33 Aschliman *et al.*, 2012; #16 de Carvalho & Maisey, 1996; #25 McEachran *et al.*, 1996; #33 & #34 & #35 Shirai, 1992; #17 Nishida, 1990).

The presence of preorbital processes has been regarded as a synapomorphy for Euselachii (Villalobos-Segura *et al.*, 2022). Revision of this character revealed that the preorbital process can originate either from the nasal capsule wall or the supraorbital shelf. This is also reflected in de Carvalho & Maisey (1996) and in Shirai (1992), who treated this character as multistate character or different characters.

1. Sup: Antorbital cartilage: [0] absent, [1] present.
   (#20 Jambura *et al.*, 2023; #23 Villalobos-Segura *et al.*, 2022; #6 Marramà *et al.*, 2020; #8 Villalobos-Segura *et al.*, 2019; #5 Claeson *et al.*, 2013; #8 Aschliman *et al.*, 2012; #2 Brito & Dutheil, 2004; #3 Nishida, 1990).

With the exception of †*Belemnobatis sismondae*, antorbital cartilages are present in all spathobatids (see Saint-Seine, 1949). We therefore modified the coding of Villalobos-Segura *et al.* (2022), who considered these skeletal structures also present in †*B. sismondae*, and coded '0' for †*B. sismondae* and '1' for †*Ae. bavarica*, †*As. platypterus*, †*Ap. seioma* gen. et sp. nov., †*K. etchesi*, and †*S bugesiacus*.

1. Sub: Antorbital cartilage (shape): [0] triangular shaped with regular outline, [1] variously shaped and with an irregular outline.
   (#21 Jambura *et al.*, 2023; #24 Villalobos-Segura *et al.*, 2022; #7 Marramà *et al.*, 2020; #9 Villalobos-Segura *et al.*, 2019).

With the exception of †*Belemnobatis sismondae*, all spathobatids have well-developed antorbital cartilages with a more or less triangular shape and a regular outline. This is also the case for †*Kimmerobatis etchesi*, as described and illustrated by Underwood & Claeson (2017). Therefore, we changed the coding of Villalobos-Segura *et al.* (2022) for this taxon from '?' to '0'. The coding for †*B. sismondae* has been changed from '0' to '-'.

1. Sub: Antorbital cartilages (with regular outline): [0] well-developed, [1] reduced.
   (#22 Jambura *et al.*, 2023; #25 Villalobos-Segura *et al.*, 2022; #9 Villalobos-Segura *et al.*, 2019).

With the exception of †*Belemnobatis sismondae*, all sptathobatids have well-developed antorbital cartilages with a more or less triangular shape and a regular outline. This is also the case for †*Kimmerobatis etchesi*, as described and illustrated by Underwood & Claeson (2017). Therefore, we changed the coding of Villalobos-Segura *et al.* (2022) for this taxon from '?' to '0'. The coding for †*B. sismondae* has been changed from '0' to '-'.

1. Sub: Anterior process of antorbital cartilage: [0] absent, [1] present.
   (#23 Jambura *et al.*, 2023; #26 Villalobos-Segura *et al.*, 2022; #9 Villalobos-Segura *et al.*, 2019; #3 Brito & Dutheil, 2004).

Anterior processes of the antorbital cartilages are not present in any of the Jurassic rays in which antorbital cartilages are present. We have therefore coded this character as '0' in these taxa, and as '-' in †*B. sismondae*.

1. Position of the articulation of the antorbital cartilage on nasal capsule: [0] lateral, [1] antero-lateral, [2] postero-lateral.
   (#24 Jambura *et al.*, 2023; #110 Villalobos-Segura *et al.*, 2022; #2 de Carvalho, 2004).

Because †*B. sismondae* lacks antorbital cartilages, we have changed the original coding by Villaobos-Segura *et al.* (2022) for this taxon from '0' to '-'.

1. Supraorbital crest: [0] present, [1] absent.
   (#28 Jambura *et al.*, 2023; #20 Villalobos-Segura *et al.*, 2022; #34 Marramà *et al.*, 2020; #36 Villalobos-Segura *et al.*, 2019; #11 Claeson *et al.*, 2013; #34 Aschliman *et al.*, 2012; #6 Brito & Dutheil, 2004; #22a & 11b Goto 2001; #26 McEachran *et al*., 1996; #32 Nishida 1990).
2. Suborbital shelf: [0] absent, [1] present.
   (#30 Jambura *et al.*, 2023; #40 Villalobos-Segura *et al.*, 2022; #3 de Carvalho & Maisey, 1996).
3. Basitrabecular process: [0] absent, [1] present.
   (#32 Jambura *et al.*, 2023; #41 Villalobos-Segura *et al.*, 2022; #3a Goto, 2001; #21 de Carvalho & Maisey, 1996; #44 Shirai, 1992).
4. Sup: Postorbital process: [0] well-developed, [1] reduced.
   (#33 Jambura *et al.*, 2023; #27 Villalobos-Segura *et al.*, 2022; #36 Marramà *et al.*, 2020; #38 Villalobos-Segura *et al.*, 2019; #12 Claeson *et al.*, 2013; #36 Aschliman *et al.*, 2012; #32 McEachran & Aschliman, 2004; #7 Brito & Dutheil, 2004; #35 Nishida, 1990).
5. Sub: Postorbital process: [0] narrow, [1] broad and shelf-like.
   (#34 Jambura *et al.*, 2023; #28 Villalobos-Segura *et al.*, 2022; #36 Marramà *et al.*, 2020; #38 Villalobos-Segura *et al.*, 2019; #36 Aschliman *et al.*, 2012; #28 McEachran *et al.*, 1996; #35 & #65 Nishida, 1990).
6. Sub: Postorbital process: [0] separated from triangular process, [1] fused with triangular process.
   (#35 Jambura *et al.*, 2023; #29 Villalobos-Segura *et al.*, 2022; #37 Aschliman *et al.*, 2012; #29 McEachran *et al.*, 1996).
7. Sub: Postorbital process: [0] projects laterally, [1] projects ventro-laterally.
   (#36 Jambura *et al.*, 2023; #30 Villalobos-Segura *et al.*, 2022; #38 Aschliman *et al.*, 2012; #30 McEachran *et al.*, 1996).
8. Sub: Jugal arch: [0] absent, [1] present.
   (#43 Jambura *et al.*, 2023; #22 Villalobos-Segura *et al.*, 2022; #39 Aschliman *et al.*, 2012; #31 McEachran *et al.*, 1996).

**Splanchnocranium**

1. Antimeres of upper and lower jaws: [0] separated, [1] fused.
   (#46 Jambura *et al.*, 2023; #78 Villalobos-Segura *et al.*, 2022; #39 Marramà *et al.*, 2020; #41 Villalobos-Segura *et al.*, 2019; #40 Aschliman *et al.*, 2012; #32 McEachran *et al.*, 1996).
2. Orbital process: [0] absent, [1] present.
   (#16 Villalobos-Segura *et al.*, 2022).
3. Ethmoidal articulation: [0] absent, [1] present.
   (#49 Jambura *et al.*, 2023; #15 Villalobos-Segura *et al.*, 2022; #47 Landemaine *et al.*, 2018; #47(35) Klug, 2010; #11 Shirai, 1996).
4. Postorbital articulation: [0] absent, [1] present (articular facet located on primary postorbital process), [2] present (on the ventrolateral part of the lateral commissure).
   (#50 Jambura *et al.*, 2023; #17 Villalobos-Segura *et al.*, 2022; #11(11) Klug, 2010; #12 de Carvalho, 1996; #15 de Carvalho & Maisey, 1996; #14 Shirai, 1996; #32 Shirai, 1992).
5. Otic process forming a quadrate flange: [0] absent, [1] present.
   (#53 Jambura *et al.*, 2023; #19 Villalobos-Segura *et al.*, 2022).
6. Meckel's cartilage: [0] not expanded laterally, [1] expanded medially.
   (#55 Jambura *et al.*, 2023; #79 Villalobos-Segura *et al.*, 2022; #40 Marramà *et al.*, 2020; #42 Villalobos-Segura *et al.*, 2019; #41 Aschliman *et al.*, 2012; #33 McEachran *et al.*, 1996).
7. Wing-like process on Meckel's cartilage: [0] absent, [1] present.
   (#56 Jambura *et al.*, 2023; #80 Villalobos-Segura *et al.*, 2022; #41 Marramà *et al.*, 2020; #43 Villalobos-Segura *et al.*, 2019; #42 Aschliman *et al.*, 2012; #34 McEachran *et al.*, 1996; #86 Nishida, 1990).
8. Ligamentous sling on Meckel’s cartilage: [0] absent, [1] present.
   (#64 Villalobos-Segura *et al.*, 2022; #72 Marramà *et al.*, 2020; #76 Villalobos-Segura *et al.*, 2019; #83 Aschliman *et al.*, 2012).
9. Hyomandibula-Meckelian ligament: [0] absent, [1] present.
   (#74 Villalobos-Segura *et al.*, 2022; #44 Marramà *et al.*, 2020; #46 Villalobos-Segura *et al.*, 2019; #45 Aschliman *et al.*, 2012).
10. Depressor mandibularis: [0] present, [1] absent.
    (#76 Villalobos-Segura *et al.*, 2022; #73 Marramà *et al.*, 2020; #77 Villalobos-Segura *et al.*, 2019; #84 Aschliman *et al.*, 2012).
11. Intermandibularis: [0] present, [1] absent, [2] modified as a narrow muscle band that originates on the hyomandibula and inserts on the posterior margin of the Meckel’s cartilage.
    (#77 Villalobos-Segura *et al.*, 2022; #82 Aschliman *et al.*, 2012).
12. Labial cartilages: [0] present, [1] absent.
    (#72 Villalobos-Segura *et al.*, 2022; #42 Marramà *et al.*, 2020; #44 Villalobos-Segura *et al.*, 2019; #43 Aschliman *et al.*, 2012).
13. Jaw support: [0] Holostyly, [1] Hyostyly, [2] Archaeostyly.
    (#14 Villalobos-Segura *et al.*, 2022).
14. Gill skeleton position: [0] partly beneath otico-occipital regions, [1] posterior to the occipital region.
    (#58 Jambura *et al.*, 2023; #140 Villalobos-Segura *et al.*, 2022; #29 Coates *et al.*, 2013).
15. Sup: Spiracularis: [0] undivided, [1] divided.
    (#59 Jambura *et al.*, 2023; #65 Villalobos-Segura *et al.*, 2022; #74 Marramà *et al.*, 2020; #78 Villalobos-Segura *et al.*, 2019; #85 Aschliman *et al.*, 2012; #61 McEachran *et al.*, 1996).
16. Sub: Spiracularis (if divided): [0] divided, one bundle enters the dorsal oral membrane underlying the neurocranium, [1] splits into lateral and medial bundles, with the medial bundles inserting onto the posterior surface of the Meckel’s cartilage and the lateral bundle onto the dorsal edge of the hyomandibula, [2] subdivided proximally and inserts separately into the palatoquadrate and the hyomandibula.
    (#60 Jambura *et al.*, 2023; #66 Villalobos-Segura *et al.*, 2022).
17. Sup: Coracohyomandibularis: [0] single origin, [1] separate origins.
    (#61 Jambura *et al.*, 2023; #68 Villalobos-Segura *et al.*, 2022; #76 Marramà *et al.*, 2020; #80 Villalobos-Segura *et al.*, 2019; #88 Aschliman *et al.*, 2012; #64 McEachran *et al.*, 1996).
18. Sub: Coracohyomandibularis (if separate origins): [0] origins in the facia supporting the insertion of the coracoarcualis and on the pericardial membrane, [1] origin on the anterior portion of the ventral gill arch region and on the pericardial membrane.
    (#69 Villalobos-Segura *et al.*, 2022)
19. Small cartilages associated with hyomandibular-Meckelian ligament: [0] absent, [1] present.
    (#64 Jambura *et al.*, 2023; #75 Villalobos-Segura *et al.*, 2022; #45 Marramà *et al.*, 2020; #47 Villalobos-Segura *et al.*, 2019; #47 Aschliman *et al.*, 2012; #38 McEachran *et al.*, 1996).
20. Medial section of hyomandibula: [0] narrow, [1] expanded.
    (#66 Jambura *et al.*, 2023; #73 Villalobos-Segura *et al.*, 2022; #43 Marramà *et al.*, 2020; #45 Villalobos-Segura *et al.*, 2019; #44 Aschliman *et al.*, 2012).
21. Pseudohyal: [0] absent, [1] present.
    (#68 Jambura *et al.*, 2023; #47 Villalobos-Segura *et al.*, 2022; #3 Marramà et al., 2020; #3 Villalobos-Segura *et al.*, 2019; #3 Aschliman *et al.*, 2012; #10 Brito & Dutheil, 2004).
22. Basihyal: [0] present, [1] absent.
    (#70 Jambura *et al.*, 2023; #44 Villalobos-Segura *et al.*, 2022; #46 Marramà *et al.*, 2020; #48 Villalobos-Segura *et al.*, 2019; #27 Claeson *et al*., 2013; #48 Aschliman *et al.*, 2012).
23. Basibranchial: [0] segmented, [1] unsegmented.
    (#37 Villalobos-Segura *et al.*, 2022).
24. Hypobranchials (second-last) direction: [0] not directed towards midline, [1] midline directed.
    (#73 Jambura *et al.*, 2023; #38 Villalobos-Segura *et al.*, 2022).

The first hypobranchial is always directed laterally, but the remaining hypobranchials can be directed towards the midline.

1. Hypobranchials-basibranchial: [0] articulated with the basibranchial, [1] fused.
   (#74 Jambura *et al.*, 2023; #42 Villalobos-Segura *et al.*, 2022).
2. First hypobranchial-basihyal: [0] separated, [1] fused [2] segmented.
   (#75 Jambura *et al.*, 2023; #45 Villalobos-Segura *et al.*, 2022).
3. Fourth hypobranchial: [0] well-developed, [1] reduced.
   (#76 Jambura *et al.*, 2023; #39 Villalobos-Segura *et al.*, 2022).
4. Ceratohyal: [0] fully developed, [1] reduced.
   (#46 Villalobos-Segura *et al.*, 2022; #48 Marramà *et al.*, 2020; #50 Villalobos-Segura *et al.*, 2019; #28 Claeson *et al*., 2013; #49 Aschliman *et al.*, 2012).
5. Last ceratobranchial: [0] free of scapulocoracoid, [1] articulates with scapulocoracoid.
   (#81 Jambura *et al.*, 2023; #43 Villalobos-Segura *et al.*, 2022; #4 Marramà *et al.*, 2020; #4 Villalobos-Segura *et al.*, 2019; #174 Landemaine *et al.*, 2018; #4 Aschliman *et al.*, 2012; #9 Brito & Dutheil, 2004; #29 Shirai, 1996; #86 Shirai, 1992; #5 Nishida, 1990).
6. Branchial electric organs: [0] absent, [1] present.
   (#83 Jambura *et al.*, 2023; #21 Villalobos-Segura *et al.*, 2022; #89 Marramà *et al.*, 2020; #94 Villalobos-Segura *et al.*, 2019; #86 Aschliman *et al.*, 2012; #18 Brito & Dutheil, 2004).
7. Sup: Coracohyoideus: [0] present, [1] absent.
   (#84 Jambura *et al.*, 2023; #70 Villalobos-Segura *et al.*, 2022; #102 Marramà *et al.*, 2020; #89 Aschliman *et al.*, 2012; #65 McEachran *et al.*, 1996).
8. Sub: Coracohyoideus (if present): [0] parallel to body axis, [1] short, [2] diagonal, [3] fused.
   (#85 Jambura *et al.*, 2023; #71 Villalobos-Segura *et al.*, 2022; #103 Marramà *et al.*, 2020; #89 Aschliman *et al.*, 2012; #65 McEachran *et al.*, 1996).

**Girdles and paired fins**

1. Ventral antimeres of scapulocoracoid: [0] fused, [1] separate.
   (#90 Jambura *et al.*, 2023; #105 Villalobos-Segura *et al.*, 2022; #81 Marramà *et al.*, 2020; #85 Villalobos-Segura *et al.*, 2019; #1 de Carvalho & Maisey, 1996; #3 Shirai, 1992).
2. Sup: Suprascapulae: [0] absent, [1] fused medially, [2] unfused medially.
   (#91+92 Jambura *et al.*, 2023; #93 Villalobos-Segura *et al.*, 2022; #6 Aschliman *et al.*, 2012; #30 Goto, 2001; #30a & #52b Goto, 2001).
3. Sub: Suprascapula interaction with axial skeleton (if fused medially): [0] interacts with axials skeleton (articulated or fused), [1] free from axial skeleton.
   (#93 Jambura *et al.*, 2023; #94 Villalobos-Segura *et al.*, 2022; #24 Brito & Dutheil, 2004).
4. Sub: Suprascapula (if interacts with axial skeleton): [0] articulates with vertebral column, [1] fused medially to synarcual, [2] fused medially and laterally to synarcual.
   (#94 Jambura *et al.*, 2023; #95 Villalobos-Segura *et al.*, 2022; #49 Marramà *et al.*, 2020; #51 Villalobos-Segura *et al.*, 2019; #50 Aschliman *et al.*, 2012; #25 Brito & Dutheil, 2004).
5. Sup: Suprascapula-scapula articulation: [0] curved, [1] crenate, [2] ball socket, [3] straight.
   (#95 Jambura *et al.*, 2023; #96 Villalobos-Segura *et al.*, 2022; #82 Marramà *et al.*, 2020; #86 Villalobos-Segura *et al.*, 2019; #53 Aschliman *et al.*, 2012).
6. Sub: Crenated suprascapula (variations): [0] with lateral projections, [1] thin upper and lower lobes, [2] upper lobe wider than lower, [3] of similar size and width.
   (#96 Jambura *et al.*, 2023; #97 Villalobos-Segura *et al.*, 2022).
7. Scapular process-scapula: [0] fused, [1] articulated.
   (#97 Jambura *et al.*, 2023; #98 Villalobos-Segura *et al.*, 2022).
8. Scapular process: [0] short and dorsally directed, [1] long, U-curved, posteriorly directed, [2] short and postero-dorsally directed.
   (#98 Jambura *et al.*, 2023; #99 Villalobos-Segura *et al.*, 2022; #53 Marramà *et al.*, 2020; #56 Villalobos-Segura *et al.*, 2019; #56 Aschliman *et al.*, 2012).
9. Scapular process: [0] without fossa, [1] with fossa.
   (#99 Jambura *et al.*, 2023; #100 Villalobos-Segura *et al.*, 2022; #54 Marramà *et al.*, 2020; #57 Villalobos-Segura *et al.*, 2019; #57 Aschliman *et al.*, 2012).
10. Mesopterygium: [0] present, [1] absent.
    (#113 Villalobos-Segura *et al.*, 2022; #61 Marramà *et al.*, 2020; #64 Villalobos-Segura *et al.*, 2019; #45 Claeson *et al*, 2013).
11. Sup: Pectoral articulation: [0] facets, [1] facets/condyles, [2] condyles.
    (#100 Jambura *et al.*, 2023; #101 Villalobos-Segura *et al.*, 2022).
12. Sub: Pectoral articulation (if condyles): [0] single (all fused), [1] single (propterygium and mesopterygium), [2] single (mesopterygium and metapterygium), [3] separated.
    (#102 Villalobos-Segura *et al.*, 2022).
13. Scapulocoracoid condyles arrangement: [0] not horizontal, [1] horizontal.
    (#101 Jambura *et al.*, 2023; #104 Villalobos-Segura *et al.*, 2022; #55 Marramà *et al.*, 2020; #58 Villalobos-Segura *et al.*, 2019; #58 Aschliman *et al.*, 2012).
14. Sub: Mesocondyle: [0] single, [1] segmented and small, [2] forming an elongated ridge.
    (#102 Jambura *et al.*, 2023; #103 Villalobos-Segura *et al.*, 2022; #56 Marramà *et al.*, 2020; #59 Villalobos-Segura *et al.*, 2019; #43 Cleason *et al.*, 2013; #59 Aschliman *et al.*, 2012).
15. Tribasal pectoral fin: [0] absent, [1] present.
    (#104 Jambura *et al.*, 2023; #141 Villalobos-Segura *et al.*, 2022; #31 Goto, 2001; #60 & #61 Shirai, 1996).
16. Proximal pectoral elements expanded distally and paddle-like: [0] absent, [1] present.
    (#116 Villalobos-Segura *et al.*, 2022; #87 Marramà *et al.*, 2020; #92 Villalobos-Segura *et al.*, 2019).
17. Paired fin rays: [0] aplesodic, [1] plesodic.
    (#115 Villalobos-Segura *et al.*, 2022; #63 Marramà *et al.*, 2020; #66 Villalobos-Segura *et al.*, 2019; #68 Aschliman *et al.*, 2012).
18. Sup: Anterior extension of propterygium: [0] absent, [1] present.
    (#106 Jambura *et al.*, 2023; #107 Villalobos-Segura *et al.*, 2022; #57 Marramà *et al.*, 2020; #60 Villalobos-Segura *et al.*, 2019; #62 Aschliman *et al.*, 2012).
19. Sub: First segmentation of propterygium (if propterygium extends anterior): [0] not reaching nasal capsules, [1] reaching nasal capsules, [2] beyond nasal capsules.
    (#107 Jambura *et al.*, 2023; #108 Villalobos-Segura *et al.*, 2022; #15 Brito & Dutheil, 2004).
20. Proximal section of propterygium: [0] does not surpass the procondyle, [1] extend behind procondyle.
    (#108 Jambura *et al.*, 2023; #112 Villalobos-Segura *et al.*, 2022; #59 Marramà *et al.*, 2020; #62 Villalobos-Segura *et al.*, 2019; #64 Aschliman *et al.*, 2012).
21. Distal portion of Mesopterygium anteriorly projected: [0] differently shaped, [1] similarly shaped.
    (#110 Jambura *et al.*, 2023; #106 Villalobos-Segura *et al.*, 2022; #88 Marramà *et al.*, 2020; #93 Villalobos-Segura *et al.*, 2019).
22. Interaction between mesopterygium and propterygium: [0] fused, [1] separated.
    (#109 Villalobos-Segura *et al.*, 2022).
23. Pectoral fin radials: [0] all articulate to pterygia, [1] some articulate directly with scapulocoracoid.
    (#112 Jambura *et al.*, 2023; #114 Villalobos-Segura *et al.*, 2022; #60 Marramà *et al.*, 2020; #63 Villalobos-Segura *et al.*, 2019; #47 Claeson *et al.*, 2013; #65 Aschliman *et al.*, 2012; #28 Brito & Dutheil, 2004; #58 McEachran & Aschliman, 2004; #48 McEachran *et al*., 1996).

As the coding of Villalobos-Segura *et al.* (2022) for †*S. bugesiacus* is mainly based on specimens that were later described as the new species †*Ae. bavarica* (see Türtscher *et al.*, 2024), we had to change the coding for this character; while in †*Ae. bavarica* (as well as in †*Ap. seioma* gen. et sp. nov., †*As. platypterus*, †*B. sismondae*, and †*K. etchesi*) all radials articulate with the three basal cartilages ('0'), †*S. bugesiacus* is the only known Late Jurassic ray in which a radial articulates directly with the scapulocoracoid. We therefore coded this character for †*S. bugesiacus* as '1' instead of '0' (see Figure 1).


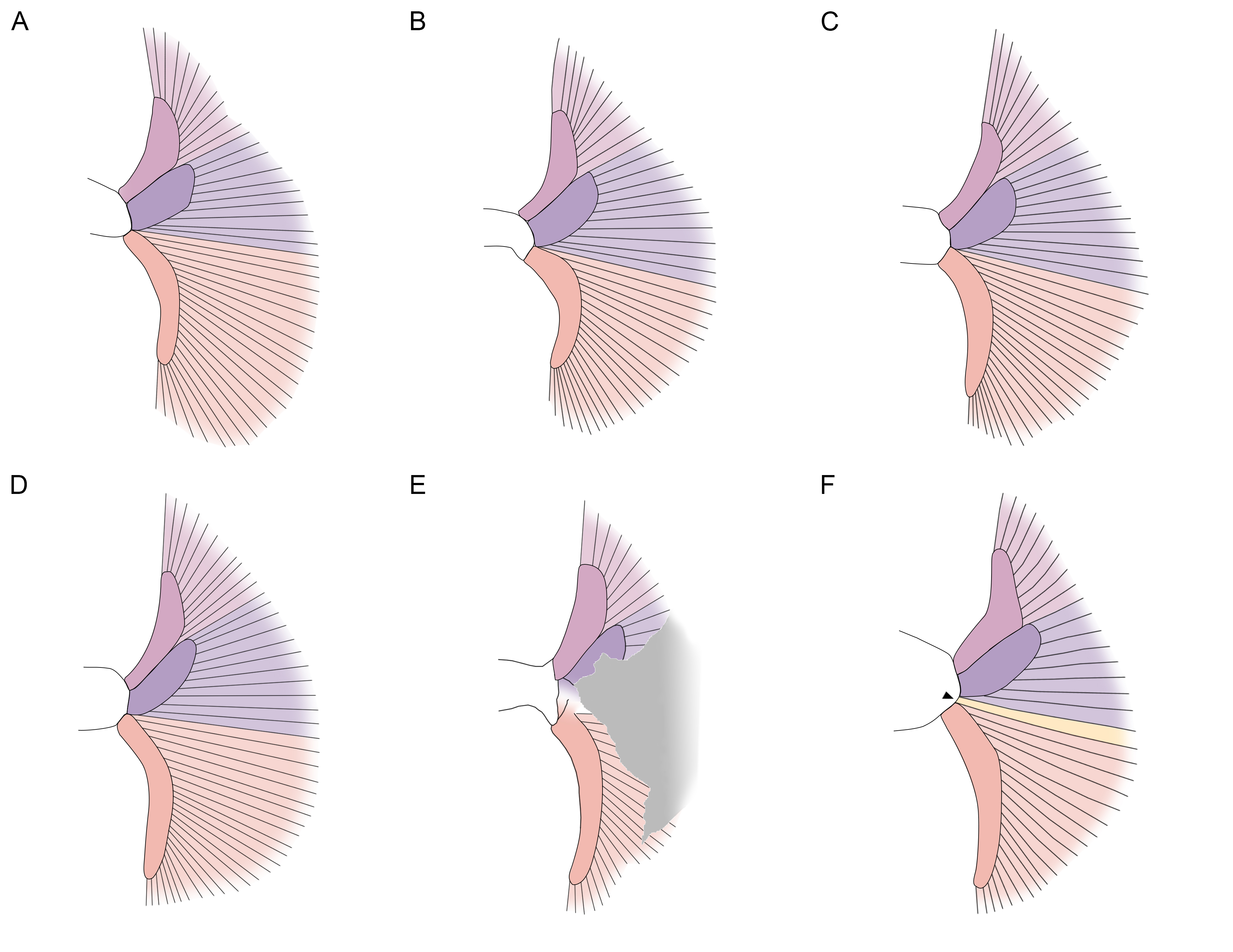
Figure 1. Schematic illustration of the scapulocoracoid, the three basal cartilages, and the pectoral radials of Late Jurassic batomorph taxa. (A) †*Aellopobatis bavarica*, LF 2323; (B) †*Apolithabatis seioma*, gen. et sp. nov., DMA-JP-2010/007; (C) †*Asterodermus platypterus,* JME SOS-3647; (D) †*Belemnobatis sismondae*, MGL 38-774; (E) †*Kimmerobatis etchesi*, MJML K874 (note that the area marked in grey represents the incomplete preservation of the specimen. The exact contours of meso- and metapterygium could not be determined here); (F) †*Spathobatis bugesiacus*, NHMUK P 2099. Arrowhead indicates radial that articulates directly with the scapulocoracoid. Not to scale.

1. Sup: Radial calcification: [0] crustal, [1] catenated.
   (#113 Jambura *et al.*, 2023; #91 Villalobos-Segura *et al.*, 2022; #104 Marramà *et al.*, 2020).
2. Sub: Radial calcification (if catenated): [0] two chains, [1] four chains.
   (#92 Villalobos-Segura *et al.*, 2022).
3. Pectoral fin with interradial connections ('cross-braces'): [0] absent, [1] present.
   (#114 Jambura *et al.*, 2023; #111 Villalobos-Segura *et al.*, 2022; #62 Marramà *et al.*, 2020; #65 Villalobos-Segura *et al.*, 2019; #67 Aschliman *et al.*, 2012; #67 Shirai, 1996).
4. Coracobrachialis: [0] consists of three to five components, [1] single component.
   (#67 Villalobos-Segura *et al.*, 2022; #75 Marramà *et al.*, 2020; #79 Villalobos-Segura *et al.*, 2019; #87 Aschliman *et al.*, 2012).
5. Pelvic girdle: [0] separated, [1] fused.
   (#115 Jambura *et al.*, 2023; #124 Villalobos-Segura *et al.*, 2022).
6. Pelvic basipterygium: [0] fused to first radial, [1] separated from first radial.
   (#116 Jambura *et al.*, 2023; #60 Villalobos-Segura *et al.*, 2022).
7. Lateral prepelvic process: [0] reduced and triangular, [1] elongated.
   (#12 da Silva *et al.*, 2023; #117 Jambura *et al.*, 2023; #117 Villalobos-Segura *et al.*, 2022; #90 Marramà *et al.*, 2020; #95 Villalobos-Segura *et al.*, 2019; #36 McEachran & Dunn, 1998).

McEachran and Dunn (1998) originally distinguished between three states ([0] short to moderately long, [1] extremely long with acute tips, [2] extremely long with biramous tips), which were transformed to binary states ([0] absent, [1] present) by Villalobos-Segura *et al.* (2022), because the different states of presence are often not preserved in fossil taxa. According to the latter authors, the presence of lateral prepelvic processes is regarded as independent gains and respective synapomorphies for Torpediniformes and Rajiformes (clades 7 and 18 in Villalobos-Segura *et al.*, 2022). Although we acknowledge that breaking down the character to a binary state is reasonable, stating that lateral prepelvic processes are not present in taxa outside these two clades is oversimplified, because all rays have lateral prepelvic processes to some extent (da Silva *et al.*, 2020). In order to avoid any misconceptions and confusion about this character, we follow the binary state proposed by Villalobos-Segura *et al.* (2022), but rename the states ([0] reduced and triangular, [1] elongated).

1. Postpelvic processes: [0] absent, [1] present.
   (#118 Jambura *et al.*, 2023; #118 Villalobos-Segura *et al.*, 2022; #69 da Silva *et al*., 2020; #91 Marramà *et al.*, 2020; #37 Cleason *et al.*, 2013; #20 Brito & Dutheil, 2004; #50 Nishida, 1990).

Postpelvic processes are small and shallow rounded projections that are present on the posteromedian margin of the puboischiadic bar (da Silva *et al.*, 2020). Originally, this character has been regarded as a synapomorphy for the members of the family Platyrhinidae (Nishida 1990; McEachran *et al.*, 1996; McEachran & Aschliman 2004; Aschliman *et al.*, 2012) but was later shown to be also present in some Torpediniformes and Rhinopristiformes (da Silva *et al.*, 2020). According to the study of Villalobos-Segura *et al.* (2022), postpelvic processes are present in Jurassic batomorphs, Torpediniformes (except *Narke* and *Temera*), Platyrhinidae, Rhinopristiformes (except *Pristis*, *Rhina*, and *Rhynchobatus*), and *Hemiscyllium*. According to da Silva *et al.* (2020), postpelvic processes are absent in *Aptychotrema*, however, we could clearly identify them as present in the examined material, agreeing with the interpretation of Villalobos-Segura *et al.* (2022). Villalobos-Segura *et al.* (2022) coded this character to be present in the Late Jurassic batomorphs †*Asterodermus*, †*Belemnobatis*, †*Kimmerobatis*, and †*Spathobatis* and recovered the presence of postpelvic processes as a synapomorphy for a Late Jurassic batomorph clade. Our revision of the Late Jurassic batomorphs revealed the presence of these processes only in †*Spathobatis*, whilst the other Late Jurassic batomorphs, including †*Aellopobatis* and †*Apolithabatis* gen. nov., did not show this feature (see Figure 2).


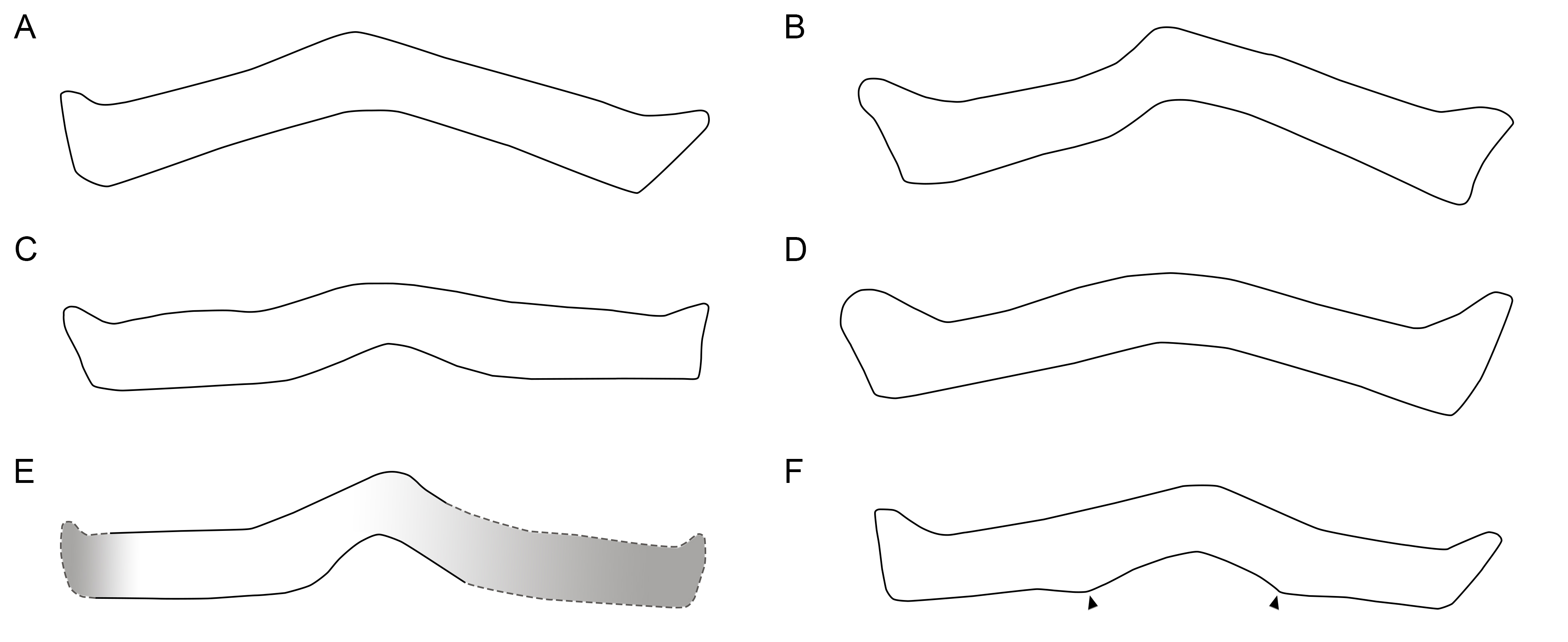


Figure 2. Pelvic girdle of Late Jurassic batomorph taxa. (A) †*Aellopobatis bavarica*, SNSB-BSPG AS I 1377; (B) †*Apolithabatis seioma*, gen. et sp. nov., DMA-JP-2010/007; (C) †*Asterodermus platypterus,* JME SOS-3647; (D) †*Belemnobatis sismondae*, NRM P 1569; (E) †*Kimmerobatis etchesi*, MJML K874 (note that the area indicated by the dotted line and the grey scale is an estimate due to the incomplete preservation of the specimen); (F) †*Spathobatis bugesiacus*, FSL 20-015-307. Arrowheads indicate postpelvic processes. Not to scale.

1. Sup: Posterior margin of puboischiadic bar: [0] straight or anteriorly directed, [1] posteriorly directed.
   (#119 Jambura *et al.*, 2023; #119 Villalobos-Segura *et al.*, 2022).
2. Sub: Anterior margin of puboischiadic bar (if posterior face straight or anteriorly directed): [0] straight, [1] anteriorly arched.
   (#120 Jambura *et al.*, 2023; #120 Villalobos-Segura *et al.*, 2022).
3. Medial process of puboischiadic bar: [0] absent, [1] present.
   (#121 Jambura *et al.*, 2023; #121 Villalobos-Segura *et al.*, 2022; #70 Aschliman *et al.*, 2012).
4. Sup: Overdevelopment of first pelvic radial: [0] absent, [1] present.
   (#123 Jambura *et al.*, 2023; #122 Villalobos-Segura *et al.*, 2022).
5. Sub: First pelvic radial (if not overdeveloped): [0] band-like, [1] slightly expanded distally, articulating with several segments in a parallel fashion, [2] rod-like and articulates with a single radial segment in a series.
   (#124 Jambura *et al.*, 2023; #123 Villalobos-Segura *et al.*, 2022; #65 Marramà *et al.*, 2020; #68 Villalobos-Segura *et al.*, 2019; #71 Aschliman *et al.*, 2012).
6. Reduced number of cartilages between pelvic basipterygium and clasper: [0] absent, [1] present.
   (#125 Jambura *et al.*, 2023; #125 Villalobos-Segura *et al.*, 2022).
7. Dorsal margin of clasper cartilage: [0] lacks medial flange, [1] possesses medial flange.
   (#126 Jambura *et al.*, 2023; #127 Villalobos-Segura *et al.*, 2022; #68 Marramà *et al.*, 2020; #71 Villalobos-Segura *et al.*, 2019; #75 Aschliman *et al.*, 2012).
8. Clasper length: [0] short, [1] long.
   (#126 Villalobos-Segura *et al.*, 2022; #67 Marramà *et al.*, 2020; #70 Villalobos-Segura *et al.*, 2019; #73 Aschliman *et al.*, 2012).
9. Ventral terminal cartilages: [0] simple, [1] free distally, forming sentinel elements or fused with ventral marginal cartilages, [2] folded ventrally along their long axes to form a convex flange.
   (#128 Jambura *et al.*, 2023; #128 Villalobos-Segura *et al.*, 2022; #69 Marramà *et al.*, 2020; #73 Villalobos-Segura *et al.*, 2019; #78 Aschliman *et al.*, 2012).
10. Ventral terminal cartilages: [0] attached over length to axial cartilages, [1] free of axial cartilages.
    (#129 Jambura *et al.*, 2023; #129 Villalobos-Segura *et al.*, 2022; #70 Marramà *et al.*, 2020; #74 Villalobos-Segura *et al.*, 2019; #79 Aschliman *et al.*, 2012).

Axial skeleton

1. Cervical vertebra: [0] unmodified, [1] synarcual, product of expansion of vertebral centra, [2] fusion of neural/basidorsal and haemal/basiventral elements.
   (#134 Jambura *et al.*, 2023; #48 Villalobos-Segura *et al.*, 2022; #5 Aschliman *et al.*, 2012; #23 Brito & Dutheil, 2004).
2. Lateral stays: [0] fused with medial crest, [1] free of medial crest.
   (#135 Jambura *et al.*, 2023; #51 Villalobos-Segura *et al.*, 2022).
3. Orientation of lateral stays: [0] dorsally directed, [1] laterally directed.
   (#136 Jambura *et al.*, 2023; #52 Villalobos-Segura *et al.*, 2022; #50 Marramà *et al.*, 2020; #53 Villalobos-Segura *et al.*, 2019; #51 Aschliman *et al.*, 2012).
4. Ventral occipital-synarcual articulation: [0] synarcual lip firmly fitted into notch in basicranium, [1] synarcual lip rests in foramen magnum, [2] synarcual lip reduced, with a paired connection into notch in basicranium.
   (#137 Jambura *et al.*, 2023; #53 Villalobos-Segura *et al.*, 2022; #51 Marramà *et al.*, 2020; #54 Villalobos-Segura *et al.*, 2019; #52 Aschliman *et al.*, 2012).
5. Position of vertebral centra in the synarcual relative to position of suprascapula in the synarcual: [0] present the entire length, [1] reaching rostral to suprascapula, [2] reaching caudal to suprascapula.
   (#138 Jambura *et al.*, 2023; #55 Villalobos-Segura *et al.*, 2022; #78 Marramà *et al.*, 2020; #82 Villalobos-Segura *et al.*, 2019).
6. Occipital hemicentrum: [0] absent, [1] present.
   (#139 Jambura *et al.*, 2023; #50 Villalobos-Segura *et al.*, 2022; #16 Landemaine *et al.*, 2018; #16(16) Klug, 2010; #17 de Carvalho, 1996; #29 de Carvalho & Maisey, 1996; #21 Shirai, 1996; #53 Shirai, 1992).
7. Expanded basiventral process of cervical vertebrae: [0] absent, [1] present.
   (#140 Jambura *et al.*, 2023; #49 Villalobos-Segura *et al.*, 2022; #16-18 Maisey *et al.*, 2020).
8. Vertebral ribs: [0] absent, [1] present.
   (#146 Jambura *et al.*, 2023; #90 Villalobos-Segura *et al.*, 2022; #92 Marramà *et al.*, 2020; #44 Aschliman *et al.*, 2012; #105 Klug, 2010; #49 McEachran & Aschliman, 2004; #34a Goto, 2001; #44 McEachran *et al*., 1996; #74 Shirai, 1996; #158 Shirai, 1992; #64 Nishida, 1990).
9. Arcualia dorsalis: [0] absent, [1] present.
   (#148 Jambura *et al*., 2023; #54 Villalobos-Segura *et al*., 2022; #77 Marramà *et al.*, 2020; #81 Villalobos-Segura *et al.*, 2019; #30 Brito *et al.*, 2013).
10. Second synarcual: [0] absent, [1] present.
    (#149 Jambura *et al.*, 2023; #88 Villalobos-Segura *et al.*, 2022; #52 Marramà *et al.*, 2020; #55 Villalobos-Segura *et al.*, 2019; #54 Aschliman *et al.*, 2012; #43 McEachran *et al.*, 1996; #66 Nishida, 1990).
11. Caudal vertebrae: [0] diplospondylus, [1] fused.
    (#150 Jambura *et al.*, 2023; #89 Villalobos-Segura *et al.*, 2022; #71 Marramà *et al.*, 2020; #75 Villalobos-Segura *et al.*, 2019; #80 Aschliman *et al.*, 2012).

**External features**

1. Sup: Cephalic lobes: [0] absent, [1] present.
   (#160 Jambura *et al.*, 2023; #61 Villalobos-Segura *et al.*, 2022; #8 Marramà *et al.*, 2020; #10 Villalobos-Segura *et al.*, 2019; #10 Aschliman *et al.*, 2012; #9 McEachran *et al.*, 1996; #96 Nishida, 1990).
2. Sub: Cephalic lobes (number): [0] single, [1] two lobes.
   (#62 Villalobos-Segura *et al.*, 2022; #9 Marramà *et al.*, 2020; #11 Villalobos-Segura *et al.*, 2019; #10 Aschliman *et al.*, 2012).
3. Spiracular tentacle: [0] absent, [1] present.
   (#63 Villalobos-Segura *et al.*, 2022; #10 Marramà *et al.*, 2020; #12 Villalobos-Segura *et al.*, 2019; #12 Aschliman *et al.*, 2012).
4. Upper eyelid: [0] present, [1] absent.
   (#161 Jambura *et al.*, 2023; #1 Villalobos-Segura *et al.*, 2022; #1 Marramà *et al.*, 2020; #1 Villalobos-Segura *et al.*, 2019; #1 of Aschliman *et al.*, 2012, #1 McEachran *et al.*, 1996).

In batomorphs, the cornea is fused with the skin of the skull dorsally, therefore no upper eyelid is present in this group.

1. Nostrils: [0] separated, [1] close together.
   (#163 Jambura *et al.*, 2023; #36 Villalobos-Segura *et al.*, 2022).
2. Anterior nasal lobe: [0] fails to reach mouth, [1] reaches the mouth.
   (#164 Jambura *et al.*, 2023; #31 Villalobos-Segura *et al.*, 2022; #11 Aschliman *et al.*, 2012).
3. Anterior nasal lobe: [0] fails to cover most of the medial half of the naris, [1] well-developed.
   (#165 Jambura *et al.*, 2023; #32 Villalobos-Segura *et al.*, 2022; #11 Aschliman *et al.*, 2012).
4. Nasal curtain fringes: [0] absent, [1] present.
   (#166 Jambura *et al.*, 2023; #33 Villalobos-Segura *et al.*, 2022).
5. Infraorbital loop of suborbital and infraorbital canals: [0] absent, [1] present.
   (#167 Jambura *et al.*, 2023; #81 Villalobos-Segura *et al.*, 2022; #17 Marramà *et al.*, 2020; #19 Villalobos-Segura *et al.*, 2019; #21 Aschliman *et al.*, 2012; #15 McEachran *et al.*, 1996).
6. Subpleural loop of the hyomandibular canal: [0] broad rounded, [1] loop forms a lateral hook, [2] lateral aspects of subpleural loop are nearly parallel.
   (#168 Jambura *et al.*, 2023; #82 Villalobos-Segura *et al.*, 2022; #18 Marramà *et al.*, 2020; #20 Villalobos-Segura *et al.*, 2019; #22 Aschliman *et al.*, 2012; #16 McEachran *et al.*, 1996).
7. Sup: Abdominal canal on coracoid bar: [0] absent, [1] present.
   (#169 Jambura *et al.*, 2023; #83 Villalobos-Segura *et al.*, 2022; #20 Marramà *et al.*, 2020; #22 Villalobos-Segura *et al.*, 2019; #24 Aschliman *et al.*, 2012; #18 McEachran *et al.*, 1996).
8. Sub: Abdominal canal on coracoid bar (if present): [0] groove-cephalic lateral line forms abdominal canal on coracoid bar, [1] pores.
   (#170 Jambura *et al.*, 2023; #84 Villalobos-Segura *et al.*, 2022; #21 Marramà *et al.*, 2020; #23 Villalobos-Segura *et al.*, 2019; #24 Aschliman *et al.*, 2012).
9. Lateral tubes of subpleural loop: [0] unbranched, [1] branched.
   (#85 Villalobos-Segura *et al.*, 2022; #19 Marramà *et al.*, 2020; #21 Villalobos-Segura *et al.*, 2019; #23 Aschliman *et al.*, 2012).
10. Scapular loops of scapular canals: [0] absent, [1] present.
    (#171 Jambura *et al.*, 2023; #86 Villalobos-Segura *et al.*, 2022; #22 Marramà *et al.*, 2020; #24 Villalobos-Segura *et al.*, 2019; #25 Aschliman *et al.*, 2012; #19 McEachran *et al.*, 1996).
11. Cephalic lateral line canals on ventral surface: [0] present, [1] absent.
    (#172 Jambura *et al.*, 2023; #87 Villalobos-Segura *et al.*, 2022; #20 Aschliman *et al.*, 2012; #14 McEachran *et al.*, 1996).
12. Cephalic spines: [0] absent, [1] present.
    (#173 Jambura *et al.*, 2023; #2 Villalobos-Segura *et al.*, 2022).
13. Placoid scales: [0] scarce or absent, [1] present.
    (#174 Jambura *et al.*, 2023; #133 Villalobos-Segura *et al.*, 2022; #13 Marramà *et al.*, 2020; #15 Villalobos-Segura *et al.*, 2019; #15 Aschliman *et al.*, 2012; #11 McEachran & Dunn, 1998).
14. Malar and alar thorns: [0] absent, [1] present.
    (#175 Jambura *et al.*, 2023; #134 Villalobos-Segura *et al.*, 2022; #14 Marramà  *et al.*, 2020; #16 Villalobos-Segura *et al.*, 2019; #17 Aschliman *et al.*, 2012; #21 Brito & Dutheil, 2004; #22 McEachran & Dunn, 1998).
15. Enlarged dermal scales (thorns): [0] absent, [1] present.
    (#135 Villalobos-Segura *et al.*, 2022; #16 Aschliman *et al.*, 2012).
16. Lateral rostral dermal denticles: [0] absent, [1] present.
    (#176 Jambura *et al.*, 2023; #136 Villalobos-Segura *et al.*, 2022; #86 Marramà *et al.*, 2020; #90 Villalobos-Segura *et al.*, 2019).
17. Ventral rostral series: [0] absent, [1] present.
    (#137 Villalobos-Segura *et al.*, 2022; #91 Villalobos-Segura *et al.*, 2019).
18. Sup: Two dorsal fin spines: [0] absent, [1] present.
    (#177 Jambura *et al.*, 2023; #130 Villalobos-Segura *et al.*, 2022; #12a Goto, 2001; #49 de Carvalho, 1996).

Villalobos-Segura *et al.* (2022) coded dorsal fin spines as absent in †*Spathobatis bugesiacus* and as unknown ('?') in †*Asterodermus platypterus*. The supposed †*S. bugesiacus* specimens they examined included three specimens that were previously included in †*S. bugesiacus*, but which we now know to be †*Aellopobatis bavarica* (see Türtscher *et al.*, 2024) and which do indeed lack dorsal fin spines, and two specimens of †*S. bugesiacus*, one of which lacks the trunk and one of which is a fossil print and does not show the minute fin spines. However, the re-examination of †*S. bugesiacus* by Türtscher *et al.* (2024) clearly showed that two small fin spines are present in front of each dorsal fin, so we changed the coding of this character to 'present' in †*S. bugesiacus*. Most †*As. platypterus* specimens lack the caudal fin, dorsal fins, and fin spines. In the holotype (NHMUK PV P 12067), however, the very small fin spines are preserved, and we therefore have changed the coding for the character 'two dorsal fin spines' to 'present' as well (see Figure 3).


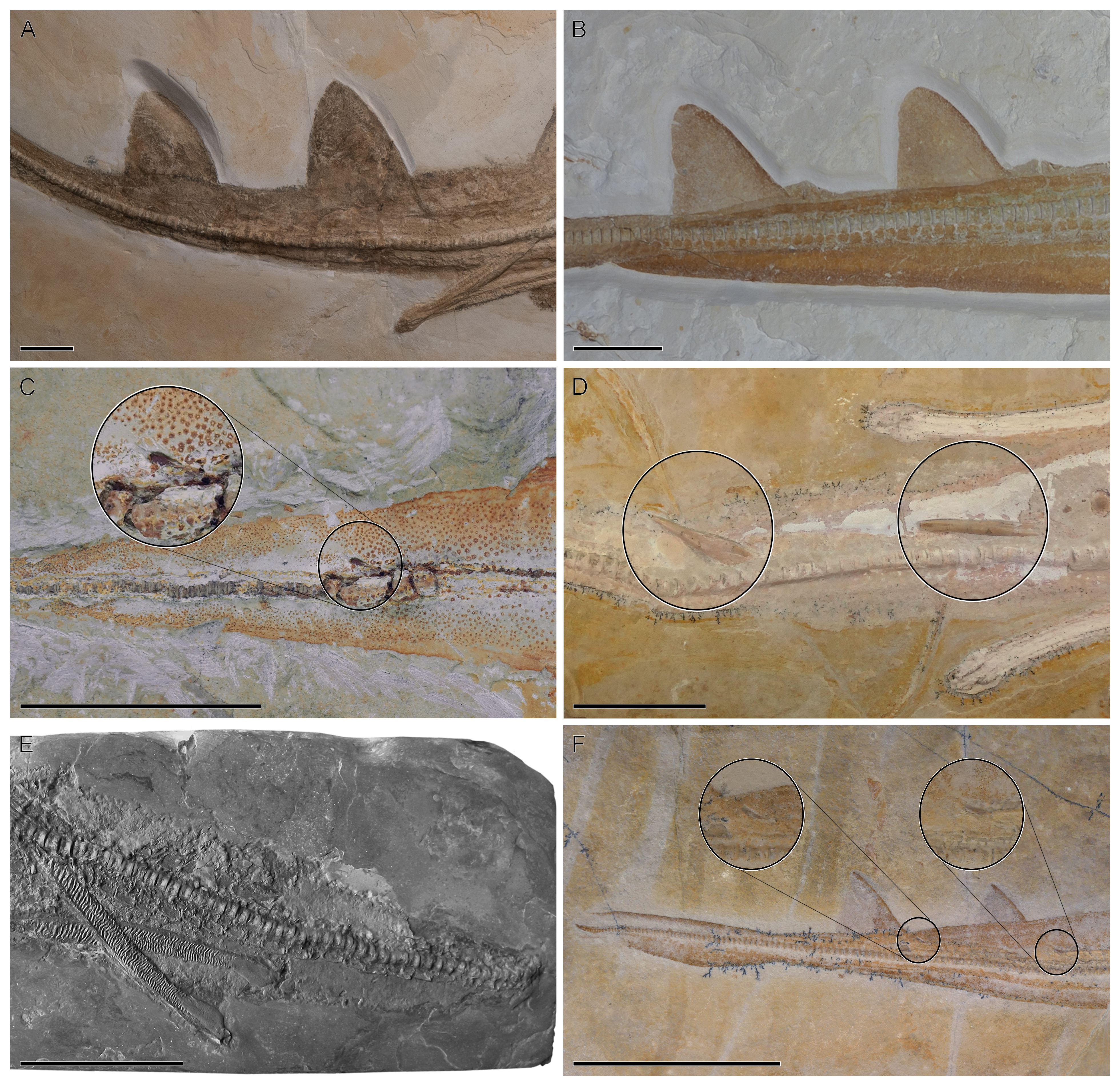
Figure 3. Region of the dorsal fins in Late Jurassic batomorph taxa. (A) †*Aellopobatis bavarica*, LF 2323 (no fin spines present); (B) †*Apolithabatis seioma*, gen. et sp. nov., DMA-JP-2010/007 (no fin spines present); (C) †*Asterodermus platypterus,* NHMUK P 12067 (minute fin spine highlighted and enlarged in circles); (D) †*Belemnobatis sismondae*, MDC 20015318 (large fin spines highlighted in circle); (E) †*Kimmerobatis etchesi*, MJML K1894 (no fin spines could be detected); (F) †*Spathobatis bugesiacus*, MDC 20015301 (minute fin spines highlighted and enlarged in circles). Scale bars: (A), (B), (D), (F) = 5cm; (C) = 2 cm; (E) = 10 cm.

1. Sub: Enameloid layer on fin spines: [0] absent, [1] present.
   (#131 Villalobos-Segura *et al.*, 2022).

In accordance with the coding change of character 134 compared to the original coding by Villalobos-Segura *et al.* (2022), we also changed the coding of the cohesive character 'enameloid layer on fin spines' to 'present' in †*S. bugesiacus* and †*As. platypterus*.

1. Serrated tail sting: [0] absent, [1] present.
   (#180 Jambura *et al.*, 2023; #132 Villalobos-Segura *et al.*, 2022; #12 Marramà *et al.*, 2020; #14 Villalobos-Segura *et al.*, 2019; #14 Aschliman *et al.*, 2012; #13 McEachran *et al.*, 1996).

**Dentition**

1. Differentiated lateral uvulae on teeth: [0] absent, [1] present.
   (#192 Jambura *et al.*, 2023; #56 Villalobos-Segura *et al.*, 2022; #83 Marramà *et al.*, 2020; #87 Villalobos-Segura *et al.*, 2019; #22 Claeson *et al.*, 2013).
2. Osteodentine: [0] absent, [1] present.
   (#195 Jambura *et al.*, 2023; #57 Villalobos-Segura *et al.*, 2022; #15 Marramà *et al.*, 2020; #19 Aschliman *et al.*, 2012).

To the best of our knowledge, histological studies of the teeth of Late Jurassic batomorphs are still pending. While we agree that it is indeed most parsimonious to hypothesize the absence of osteodentine in the root of these taxa, we coded this character as unknown ('?') for †*Ae. bavarica*, †*As. platypterus*, †*Ap. seioma* gen. et sp. nov., †*B. sismondae*, †*K. etchesi*, and †*S. bugesiacus* instead of '0', in contrast to Villalobos-Segura *et al.* (2022), preferring to await detailed histological studies on teeth of early batomorphs.

1. Three-layered enameloid structure: [0] absent, [1] present.
   (#181 Jambura *et al.*, 2023; #58 Villalobos-Segura *et al.*, 2022; #159 Landemaine *et al.*, 2018; #163(103) Klug, 2010).
2. Pulp cavity in tooth roots: [0] present, [1] absent.
   (#59 Villalobos-Segura *et al.*, 2022; #98 Marramà *et al.*, 2020; #20 Claeson *et al.*, 2013).

Note that #194 in Jambura *et al.* (2023) cannot be treated as the same character, as it refers to the presence or absence of a pulp cavity in general (either in the root or crown).

1. Second transverse keel on teeth: [0] absent, [1] present.
   (#138 Villalobos-Segura *et al.*, 2022).
2. File of enlarged caniniform teeth in the upper jaw: [0] absent, [1] present.
   (#139 Villalobos-Segura *et al.*, 2022).

**References**

Aschliman, N.C.; Claeson, K.M.; McEachran, J.D. (2012). Phylogeny of Batoidea. In *Biology of Sharks and Their Relatives*; CRC Press; pp. 57–95 ISBN 978-0-429-10654-5.

Brito, P. M., & Dutheil, D. B. (2004). A preliminary systematic analysis of Cretaceous guitarfishes from Lebanon. *Mesozoic fishes*, *3*, 101-109.

Brito, P. M., Leal, M. E. C., & Gallo, V. (2013). A new lower Cretaceous guitarfish (Chondrichthyes, Batoidea) from the Santana formation, Northeastern Brazil. *Boletim do Museu Nacional, Geologia*, *75*, 1-13.

de Carvalho, M. R. (2004). A Late Cretaceous thornback ray from southern Italy, with a phylogenetic reappraisal of the Platyrhinidae (Chondrichthyes: Batoidea). *Mesozoic fishes*, *3*, 75-100.

de Carvalho, M. R., Maisey, J. G., & Arratia, G. (1996). Phylogenetic relationships of the late jurassic shark *Protospinax* Woodward 1919 (Chondrichthyes: Elasmobranchii). *Mesozoic fishes—systematics and paleoecology*, 9-46.

Claeson, K. M., Underwood, C. J., & Ward, D. J. (2013). †*Tingitanius tenuimandibulus*, a new platyrhinid batoid from the Turonian (Cretaceous) of Morocco and the Cretaceous radiation of the Platyrhinidae. *Journal of Vertebrate Paleontology*, *33*(5), 1019-1036.

Coates, M. I., Gess, R. W., Finarelli, J. A., Criswell, K. E., & Tietjen, K. (2017). A symmoriiform chondrichthyan braincase and the origin of chimaeroid fishes. *Nature*, *541*(7636), 208-211.

Goto, T. (2001). Comparative anatomy, phylogeny and cladistic classification of the order Orectolobiformes (Chondrichthyes, Elasmobranchii). *Memoirs of the graduate school of fisheries sciences, Hokkaido University*, *48*(1), 1-100.

Jambura, P. L., Villalobos-Segura, E., Türtscher, J., Begat, A., Staggl, M. A., Stumpf, S., Kindlimann, R., Klug, S., Lacombat, F., Pohl, B., Maisey, J. G., Naylor, G. J. P., & Kriwet, J. (2023). Systematics and phylogenetic interrelationships of the enigmatic late Jurassic shark *Protospinax annectans* Woodward, 1918 with comments on the shark–ray sister group relationship. *Diversity*, *15*(3), 311.

Klug, S. (2010). Monophyly, phylogeny and systematic position of the† Synechodontiformes (Chondrichthyes, Neoselachii). *Zoologica scripta*, *39*(1), 37-49.

Landemaine, O., Thies, D., & Waschkewitz, J. (2018). The Late Jurassic shark *Palaeocarcharias* (Elasmobranchii, Selachimorpha)–functional morphology of teeth, dermal cephalic lobes and phylogenetic position. *Palaeontographica Abteilung A*, 103-165.

Marramà, G., Carnevale, G., Claeson, K. M., Naylor, G. J., & Kriwet, J. (2020). Revision of the Eocene ‘*Platyrhina*’ species from the Bolca Lagerstätte (Italy) reveals the first panray (Batomorphii: Zanobatidae) in the fossil record. *Journal of Systematic Palaeontology*, *18*(18), 1519-1542.

McEachran, J. D., Dunn, K. A., & Miyake, T. (1996). Interrelationships of the batoid fishes (Chondrichthyes: Batoidea). *Interrelationships of fishes*, 63-84.

McEachran, J. D., & Dunn, K. A. (1998). Phylogenetic analysis of skates, a morphologically conservative clade of elasmobranchs (Chondrichthyes: Rajidae). *Copeia*, 271-290.

McEachran, J. D., & Aschliman, N. (2004). Phylogeny of batoidea. *Biology of sharks and their relatives*, (Boca Raton (FL), 79-113.

Nishida, K. (1990). Phylogeny of the suborder Myliobatidoidei. *Memoirs of the faculty of fisheries Hokkaido University*, *37*(1-2), 1-108.

Saint-Seine, P. D. (1949). Les poissons des calcaires lithographiques de Cerin (Ain). *Publications du musée des Confluences*, *2*(1), 3-79.

Shirai, S. (1992). Squalean phylogeny: a new framework of 'squaloid' sharks and related taxa. *Ph.D. diss., Hokkaido Univ. Press*.

Shirai, S. (1996). Phylogenetic interrelationships of neoselachians (Chondrichthyes: Euselachii). *Interrelationships of fishes*, *2*, 9-34.

da Silva, J. P., Shimada, K., & Datovo, A. (2023). The importance of the appendicular skeleton for the phylogenetic reconstruction of lamniform sharks (Chondrichthyes: Elasmobranchii). *Journal of Morphology*, *284*(5), e21585.

Villalobos-Segura, E., Underwood, C. J., Ward, D. J., & Claeson, K. M. (2019). The first three-dimensional fossils of Cretaceous sclerorhynchid sawfish: *Asflapristis cristadentis* gen. et sp. nov., and implications for the phylogenetic relations of the Sclerorhynchoidei (Chondrichthyes). *Journal of Systematic Palaeontology*, *17*(21), 1847-1870.

Villalobos-Segura, E., Marramà, G., Carnevale, G., Claeson, K. M., Underwood, C. J., Naylor, G. J., & Kriwet, J. (2022). The phylogeny of rays and skates (Chondrichthyes: Elasmobranchii) based on morphological characters revisited. *Diversity*, *14*(6), 456.
