# Supplementary material for: Insights into stem Batomorphii: A new holomorphic ray (Chondrichthyes, Elasmobranchii) from the upper Jurassic of Germany
Source: PLoS One. 2025 Jan 23;20(1):e0310174. doi: 10.1371/journal.pone.0310174 (PMC11756912; doi:10.1371/journal.pone.0310174)
Supplement: S9 File — (PDF) [file pone.0310174.s009.pdf]

Supporting material 2 for:

Insights into stem Batomorphii: A new holomorphic ray (Chondrichthyes, Elasmobranchii) from the Upper Jurassic of Germany

JULIA TÜRTSCHER, PATRICK L. JAMBURA, FREDERIK SPINDLER, and JÜRGEN KRIWET

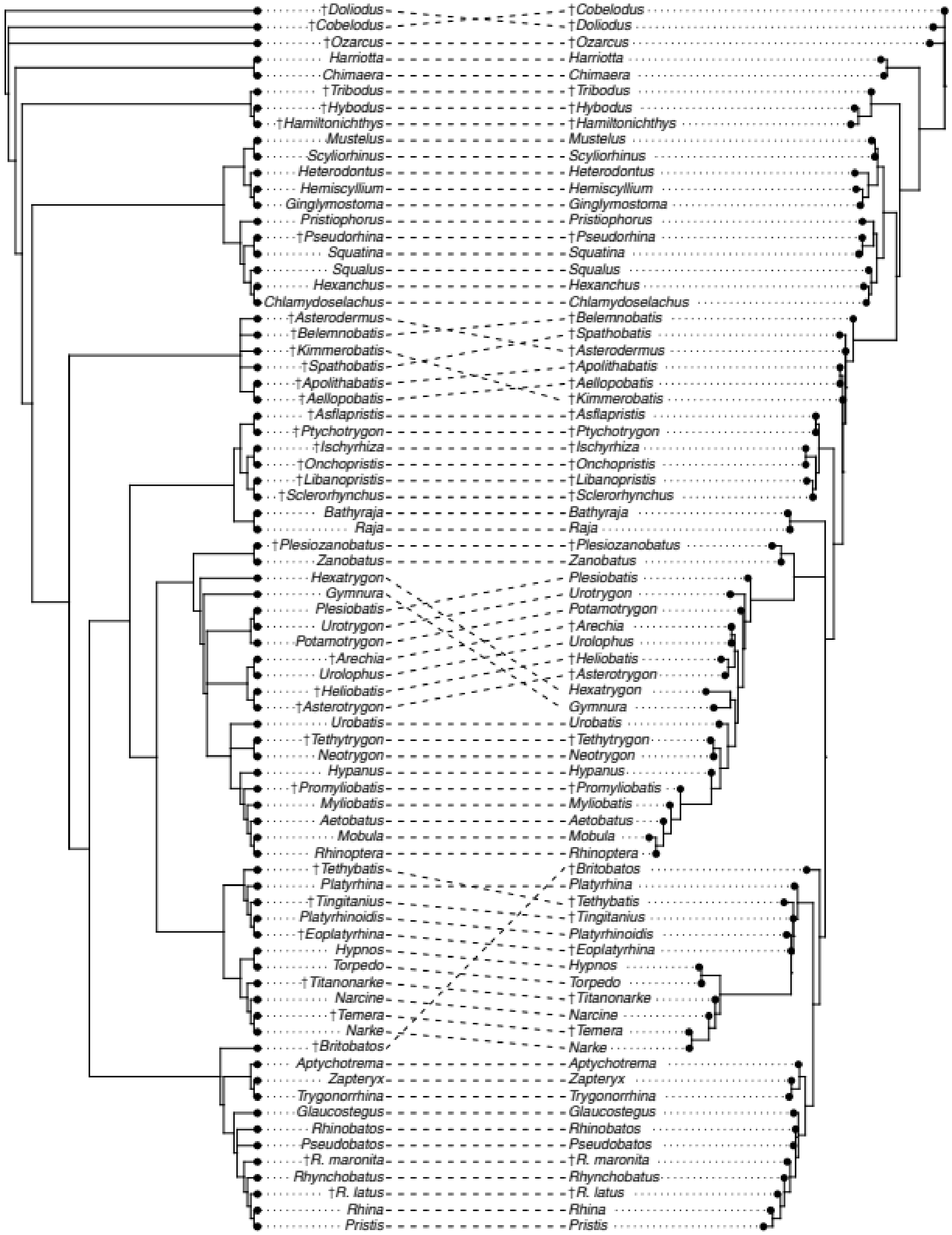

Figure S6. Phylogenetic trees obtained from two different optimality criteria; left, maximum parsimony (majority rule consensus tree); right, maximum-likelihood.
