# Supplementary material for: Insights into stem Batomorphii: A new holomorphic ray (Chondrichthyes, Elasmobranchii) from the upper Jurassic of Germany
Source: PLoS One. 2025 Jan 23;20(1):e0310174. doi: 10.1371/journal.pone.0310174 (PMC11756912; doi:10.1371/journal.pone.0310174)
Supplement: Attachment — Submitted filename: PONE-D-24-14855_annotated-JA.pdf [file pone.0310174.s011.pdf]

PLOS ONE

Insights into stem Batomorphii: A new holomorphic ray (Chondrichthyes, Elasmobranchii) from the Upper Jurassic of Germany

--Manuscript Draft--

**Manuscript Number:**	PONE-D-24-14855
**Article Type:**	Research Article
**Full Title:**	Insights into stem Batomorphii: A new holomorphic ray (Chondrichthyes, Elasmobranchii) from the Upper Jurassic of Germany
**Short Title:**	New Late Jurassic ray from Germany
**Corresponding Author:**	Julia Türtscher<br>University of Vienna Faculty of Earth Sciences Geography and Astronomy: Universitat Wien Fakultat fur Geowissenschaften Geographie und Astronomie<br>Vienna, AUSTRIA
**Keywords:**	Elasmobranchii; Batomorphii; Spathobatidae; stem group; rays; skates; Apolithabatis seioma; Apolithabatiformes; body shape; geometric morphometrics; phylogenetics; systematics; taxonomy; Mesozoic; Jurassic; Kimmeridgian; Solnhofen Archipelago; Konservat-Lagerstätte
**Abstract:**	The Late Jurassic fossil deposits of southern Germany, collectively known as the 'Solnhofen Archipelago', are one of the world's most important sources of Mesozoic vertebrates. Complete skeletons of cartilaginous fishes (Chondrichthyes), whose skeletal remains are rare in the fossil record and therefore all the more valuable, are represented, among others, by exceptionally well-preserved rays (superorder Batomorphii). Despite their potential for research in several areas, including taxonomy, morphology, ecology, and phylogeny, the number of studies on these chondrichthyans is still very limited. Here, we identify a previously unknown ray, †<i> Apolithabatis seioma</i> gen. et sp. nov., which represents the first record of a ray species from the upper Kimmeridgian of Painten, Germany, and thus the oldest Late Jurassic ray taxon from Germany based on skeletal remains. This new batomorph is characterised by a unique body shape and a combination of skeletal features that distinguish it readily from all other known Late Jurassic rays. Two different morphometric approaches confirm differences in body shape and proportions to all known Late Jurassic conspecifics. We thus extend the recent taxonomic revision of these rays and include all described holomorphic specimens in a phylogenetic framework using strict cladistic principles. The phylogenetic analysis reveals all Late Jurassic batomorphs to represent a monophyletic group, for which we introduce the new order Apolithabatiformes, which is sister to all other batomorphs representing a stem group. While the phylogenetic relationships within Apolithabatiformes ord. nov. remain largely unresolved, †<i>Apolithabatis</i> gen. nov. is placed as the sister to †<i>Aellopobatis </i>. This highlights that, despite considerable progress in our understanding of the diversity and phylogeny of early rays, difficulties remain in establishing robust relationships within batomorphs. We therefore emphasise the importance of comprehensive studies of completely preserved fossil cartilaginous fishes to obtain a better understanding of chondrichthyan evolution and their systematics in deep time.
**Order of Authors:**	Julia Türtscher
	Patrick L. Jambura
	Frederik Spindler
	Jürgen Kriwet
**Additional Information:**	
**Question**	**Response**
**Financial Disclosure**<br><br>Enter a financial disclosure statement that describes the sources of funding for the	This research was funded in whole by the Austrian Science Fund (FWF) P35357 and P33820 to JK. The funders had no role in study design, data collection and analysis, decision to publish, or preparation of the manuscript.

Powered by Editorial Manager® and ProduXion Manager® from Aries Systems Corporation

work included in this submission. Review the submission guidelines for detailed requirements. View published research articles from *PLOS ONE* for specific examples.<br><br>This statement is required for submission and **will appear in the published article** if the submission is accepted. Please make sure it is accurate.<br><br>**Unfunded studies**<br>Enter: *The author(s) received no specific funding for this work.*<br><br>**Funded studies**<br>Enter a statement with the following details:<br>• Initials of the authors who received each award<br>• Grant numbers awarded to each author<br>• The full name of each funder<br>• URL of each funder website<br>• Did the sponsors or funders play any role in the study design, data collection and analysis, decision to publish, or preparation of the manuscript?<br>• **NO** - Include this sentence at the end of your statement: *The funders had no role in study design, data collection and analysis, decision to publish, or preparation of the manuscript.*<br>• **YES** - Specify the role(s) played.<br><br>* typeset	https://www.fwf.ac.at/en/
**Competing Interests**<br><br>Use the instructions below to enter a competing interest statement for this submission. On behalf of all authors, disclose any competing interests that could be perceived to bias this work—acknowledging all financial support and any other relevant financial or non-financial competing interests.<br><br>This statement is required for submission and **will appear in the published article** if the submission is accepted. Please make sure it is accurate and that any funding sources listed in your Funding Information	The authors have declared that no competing interests exist.

later in the submission form are also declared in your Financial Disclosure statement.<br><br>View published research articles from *PLOS ONE* for specific examples.<br><br>**NO authors have competing interests**<br><br>Enter: *The authors have declared that no competing interests exist.*<br><br>**Authors with competing interests**<br><br>Enter competing interest details beginning with this statement:<br><br>*I have read the journal's policy and the authors of this manuscript have the following competing interests: [insert competing interests here]*<br><br>* typeset	
**Ethics Statement**<br><br>Enter an ethics statement for this submission. This statement is required if the study involved:<br><br>• Human participants<br>• Human specimens or tissue<br>• Vertebrate animals or cephalopods<br>• Vertebrate embryos or tissues<br>• Field research<br><br>Write "N/A" if the submission does not require an ethics statement.<br><br>General guidance is provided below. Consult the submission guidelines for detailed instructions. **Make sure that all information entered here is included in the Methods section of the manuscript.**	N/A

**Format for specific study types**<br><br>**Human Subject Research (involving human participants and/or tissue)**<br>• Give the name of the institutional review board or ethics committee that approved the study<br>• Include the approval number and/or a statement indicating approval of this research<br>• Indicate the form of consent obtained (written/oral) or the reason that consent was not obtained (e.g. the data were analyzed anonymously)<br><br>**Animal Research (involving vertebrate animals, embryos or tissues)**<br>• Provide the name of the Institutional Animal Care and Use Committee (IACUC) or other relevant ethics board that reviewed the study protocol, and indicate whether they approved this research or granted a formal waiver of ethical approval<br>• Include an approval number if one was obtained<br>• If the study involved *non-human primates*, add *additional details* about animal welfare and steps taken to ameliorate suffering<br>• If anesthesia, euthanasia, or any kind of animal sacrifice is part of the study, include briefly which substances and/or methods were applied<br><br>**Field Research**<br><br>Include the following details if this study involves the collection of plant, animal, or other materials from a natural setting:<br>• Field permit number<br>• Name of the institution or relevant body that granted permission	
**Data Availability**<br><br>Authors are required to make all data underlying the findings described fully available, without restriction, and from the time of publication. PLOS allows rare exceptions to address legal and ethical concerns. See the PLOS Data Policy and FAQ for detailed information.	Yes - all data are fully available without restriction

A Data Availability Statement describing where the data can be found is required at submission. Your answers to this question constitute the Data Availability Statement and **will be published in the article**, if accepted.<br><br>**Important:** Stating 'data available on request from the author' is not sufficient. If your data are only available upon request, select 'No' for the first question and explain your exceptional situation in the text box.<br><br>Do the authors confirm that all data underlying the findings described in their manuscript are fully available without restriction?	
**Describe where the data may be found in full sentences. If you are copying our sample text, replace any instances of XXX with the appropriate details.**<br><br>• If the data are **held or will be held in a public repository**, include URLs, accession numbers or DOIs. If this information will only be available after acceptance, indicate this by ticking the box below. For example: *All XXX files are available from the XXX database (accession number(s) XXX, XXX.)*.<br>• If the data are all contained **within the manuscript and/or Supporting Information files**, enter the following: *All relevant data are within the manuscript and its Supporting Information files.*<br>• If neither of these applies but you are able to provide **details of access elsewhere**, with or without limitations, please do so. For example:<br><br>*Data cannot be shared publicly because of [XXX]. Data are available from the XXX Institutional Data Access / Ethics Committee (contact via XXX) for researchers who meet the criteria for access to confidential data.*<br><br>*The data underlying the results presented in the study are available from (include the name of the third party*	All relevant data are within the manuscript and its Supporting Information files.

and contact information or URL).<br>• This text is appropriate if the data are owned by a third party and authors do not have permission to share the data.<br><br>* typeset	
Additional data availability information:	Tick here if the URLs/accession numbers/DOIs will be available only after acceptance of the manuscript for publication so that we can ensure their inclusion before publication.

Powered by Editorial Manager® and ProduXion Manager® from Aries Systems Corporation

Manuscript

Click here to access/download;Manuscript;Main_document.docx

Insights into stem Batomorphii: A new holomorphic ray (Chondrichthyes, Elasmobranchii) from the Upper Jurassic of Germany

Julia Türtscher[1,2*], Patrick L. Jambura[1,2], Frederik Spindler[3], Jürgen Kriwet[1,2]

[1]University of Vienna, Faculty of Earth Sciences, Geography and Astronomy, Department of Palaeontology, Evolutionary Research Group, Josef-Holaubek-Platz 2, 1090 Vienna, Austria

[2]University of Vienna, Vienna Doctoral School of Ecology and Evolution (VDSEE), Djerassiplatz 1, 1030 Vienna, Austria

[3]PALAEONAVIX, 85110 Kipfenberg, Germany

* Corresponding author

E-mail: tuertscher.julia@gmail.com

Short title: New Late Jurassic ray from Germany

Abstract

The Late Jurassic fossil deposits of southern Germany, collectively known as the 'Solnhofen Archipelago', are one of the world's most important sources of Mesozoic vertebrates. Complete skeletons of cartilaginous fishes (Chondrichthyes), whose skeletal remains are rare in the fossil record and therefore all the more valuable, are represented, among others, by exceptionally well-preserved rays (superorder Batomorphii). Despite their potential for research in several areas, including taxonomy, morphology, ecology, and phylogeny, the number of studies on these chondrichthyans is still very limited. Here, we identify a previously unknown ray, †*Apolithabatis seioma* gen. et sp. nov., which represents the first record of a ray species from the upper Kimmeridgian of Painten, Germany, and thus the oldest Late Jurassic ray taxon from Germany based on skeletal remains. This new batomorph is characterised by a unique body shape and a combination of skeletal features that distinguish it readily from all other known Late Jurassic rays. Two different morphometric approaches confirm differences in body shape and proportions to all known Late Jurassic conspecifics. We thus extend the recent taxonomic revision of these rays and include all described holomorphic specimens in a phylogenetic framework using strict cladistic principles. The phylogenetic analysis reveals all Late Jurassic batomorphs to represent a monophyletic group, for which we introduce the new order Apolithabatiformes, which is sister to all other batomorphs representing a stem group. While the phylogenetic relationships within Apolithabatiformes ord. nov. remain largely unresolved, †*Apolithabatis* gen. nov. is placed as the sister to †*Aellopobatis*. This highlights that, despite considerable progress in our understanding of the diversity and phylogeny of early rays, difficulties remain in

establishing robust relationships within batomorphs. We therefore emphasise the importance of comprehensive studies of completely preserved fossil cartilaginous fishes to obtain a better understanding of chondrichthyan evolution and their systematics in deep time.

Introduction

Chondrichthyans or cartilaginous fishes, are among the most abundant remains in the vertebrate fossil record but are usually represented only by isolated teeth (Hubbel, 1996 [1]; Maisey, 2012 [2]). Complete specimens, conversely, are very rare due to the poor preservation potential of their cartilaginous endoskeletons. Such rare 'holomorphic' fossils provide insights into body shapes, skeletal anatomy, and even soft tissue composition of long extinct taxa, allowing researchers to explore a greater variety of ecological and morphological traits in deep time. The oldest currently known holomorphic ray fossils are from the Jurassic period (Maisey et al., 2020 [3]; Türtscher et al., 2024 [4]), which evidently was an important time interval in shark and ray evolution (Guinot and Cavin, 2016 [5]). Yet, our knowledge about diversity and disparity of Jurassic rays, as well as about their systematic placement within the chondrichthyan phylogeny, is still very limited (Underwood, 2006 [6]).

The scarcity of well-preserved holomorphic chondrichthyans makes deposits that yield such extraordinary fossils (so-called 'Konservat-Lagerstätten') particularly valuable sources of information about early sharks and rays. Among the best known of these deposits is the Late Jurassic 'Solnhofen Archipelago' located in southern Germany, which consists of several individual sites of Kimmeridgian-Tithonian age (Villalobos-Segura et al., 2023 [7]). The diverse vertebrate fauna of these

extraordinary fossil sites contains several chondrichthyan taxa, including at least three holocephalian, three hybodontiform, fifteen selachimorph, and two batomorph genera (Villalobos-Segura et al., 2023 [7]). Until recently, it was assumed that two ray genera occurred in the 'Solnhofen Archipelago', i.e., †*Asterodermus* and †*Spathobatis* (Kriwet and Klug, 2015 [8]; Villalobos-Segura et al., 2023 [7]). However, a recent comprehensive study of the Late Jurassic batomorphs of Europe revealed that none of the specimens from the German deposits belong to †*Spathobatis*, which thus appears to be restricted to France. Instead, what had previously been described as a large morphotype of †*Spathobatis* was found to represent a different taxon, †*Aellopobatis* (Türtscher et al., 2024 [4]). Skeletal remains of both †*Asterodermus* and †*Aellopobatis* are known from several of the Tithonian sites in southern Germany (Türtscher et al., 2024 [4]), but to our knowledge none have been described from any of the Kimmeridgian deposits up to now. It should be noted, however, that precise provenance information is not always available for fossils from the 'Solnhofen Archipelago', especially for those collected decades ago (Villalobos-Segura et al., 2023 [7]).

Here, we describe the first holomorphic ray from the locality of Painten within the 'Solnhofen Archipelago', where upper Kimmeridgian to lower Tithonian strata are exposed and yield excellently preserved fossils. The specimen described here represents a hitherto unknown taxon from upper Kimmeridgian strata. We analysed the specimen employing morphometric approaches as proposed by Türtscher et al. (2024) [4] for taxonomic identification and included it into a phylogenetic analysis based on a revised character matrix that includes all described Late Jurassic ray species known from holomorphic specimens to resolve its phylogenetic

interrelationships.

A FIGURE OF THE GEOLOGICAL AND GEOGRAPHICAL/PALEOGEOGRAPHICAL SETTING WOULD BE APPRECIATED

Geological Setting

The specimen described here was recovered from a quarry of the Rygol Company near the village of Painten, which is located in the northern part of the so-called 'Paintener Wanne', a basin within the ‘Solnhofen Archipelago’ extending over an area of about 15 x 12 km (Albersdörfer & Häckel, 2015 [9]). This basin is located in the southeastern part of the Franconian Alb in central Bavaria, which forms, together with the Swabian Alb in the West, a low mountain range consisting predominantly of Early to Late Jurassic marine sedimentary deposits. Painten is, together with Daiting, Mörnsheim, Solnhofen, Eichstätt, and Schamhaupten in the west as well as Kelheim, Jachenhausen, Zandt, and Brunn in the south and northeast, respectively, one of the best-known fossiliferous sites in the Franconian Alb. All deposits are generally composed of fine-grained limestones representing the so-called ‘Plattenkalks’, but local sedimentological differences occur (Viohl, 2015 [10]). Moreover, the various ‘Plattenkalks‘ are not of the same age, but belong to at least four distinct formations: Torleite (Malm Epsilon), Geisental (Malm Zeta 1), Painten (Malm Zeta 1), Altmühltal (Malm Zeta 2) and Mörnsheim (Malm Zeta 3) formations, which range in age from the upper Kimmeridgian to lower Tithonian.

In the fossiliferous quarry of the Rygol Company near the village of Painten, a 5.9m thick layer of the renowned 'Kielseplattenkalk' is exposed. This layer consists of laminated, fine-grained, silicified limestone, intercalated with graded turbidite horizons composed of carbonate debris. Stratigraphically, these very dense and compact sediments are placed into the Arnstorf Member of the Torleite Formation,

which has been assigned to the *Hybnoticeras beckeri* ammonite Zone and *Lithacoceras ulmense* Subzone, indicating a latest Kimmeridgian age (Albersdörfer & Häckel, 2015 [9]).

The specimen that forms the focus of the present study was recovered in very hard layers close to the base of the exposed section of the ‘Kieselplattenkalk’. Traditional excavation using tools, therefore, was not possible, and a small quantity of explosives was detonated six metres below the surface, creating cracks in the rock without disorganising the layers. However, the top layer of rock was thrown into the air by the explosion and broke into several slabs. One of these slabs contained the specimen described here.

Material and methods

Material

A total of 55 specimens of articulated Late Jurassic batomorphs were used in the present study (Table S1). The age of the specimens ranges from the upper Kimmeridgian (Cerin, France; 'Solnhofen Archipelago' [Painten], Germany) to the lower Tithonian ('Solnhofen Archipelago' [Eichstätt, Kelheim, Solnhofen, Zandt], Germany; Kimmeridge, UK), and represent all hitherto known batomorph morphotypes of these fossil sites (see Türtscher et al., 2024 [4]). Not available for this study, however, were †*Spathobatis*? *morinicus* Sauvage, 1873 [11] from the lower Tithonian of Canjuers, France, a holomorphic batomorph based on a single specimen (Sauvage, 1873 [11]; Cavin et al., 1995 [12]), as well as an unnamed batomorph from the middle Tithonian of Argentina (Cione, 1999 [13]). Published

figures of these two specimens were not considered to be of high enough quality to be used for measurements.

All specimens were photographed with a digital camera positioned orthogonally to each specimen to avoid doubtful results due to a misaligned angle. Some specimens were examined under ultraviolet light following the technique described in Tischlinger & Arratia (2013) [14] for better identification of specific skeletal structures. Daggers before taxon names indicate extinct taxa.

Institutional abbreviations

AMNH, American Museum of Natural History, New York, USA; **BMMS**, Bürgermeister-Müller-Museum, Solnhofen, Germany; **CM**, Carnegie Museum of Natural History, Pittsburgh, USA; **DMA**, Dinosaurier Museum Altmühltal, Denkendorf, Germany; **HMNS**, Houston Museum of Natural Science, Houston, Texas, USA; **JME**, Jura-Museum Eichstätt, Eichstätt, Germany; **LF**, Lauer Foundation for Paleontology, Science and Education, Wheaton, Illinois, USA; **MB**, Museum Bergér, Eichstätt, Germany; **MCZ**, Museum of Comparative Zoology, Cambridge, Massachusetts, USA; **MGL**, Musée cantonal de Géologie, Lausanne, Switzerland; **MDC**, Musée des Confluences, Lyon, France; **MJML**, Museum of Jurassic Marine Life, Kimmeridge, UK; **MNB**, Museum für Naturkunde, Berlin, Germany; **MNHN**, Muséum National d'Histoire Naturelle, Paris, France; **NHMUK**, Natural History Museum, London, UK; **NRM**, Naturhistoriska Riksmuseet, Stockholm, Sweden; **RBINS**, Royal Belgian Institute of Natural Sciences, Brussels, Belgium; **SMNK**, Staatliches Museum für Naturkunde Karlsruhe, Karlsruhe, Germany; **SNSB-BSPG**, Bayerische

Staatssammlung für Paläontologie und Geologie, Munich, Germany; **TM**, Teylers Museum, Haarlem, Netherlands.

Anatomical abbreviations

ac, antorbital cartilage; **bp**, basipterygium; **br**, branchial arches; **c**, vertebral centra; **cf**, caudal fin; **cr**, compound radial; **d1**, first dorsal fin; **d2**, second dorsal fin; **hs**, hemal spine; **mk**, Meckel's cartilage; **ms**, mesopterygium; **mt**, metapterygium; **nc**, nasal capsule; **ns**, neural spine; **pb**, puboischiadic bar; **pp**, propterygium; **pq**, palatoquadarte; **r**, ribs; **ra**, pectoral fin radials; **rap**, pelvic fin radials; **ro**, rostrum; **sc**, scapulocoracoid; **syn**, synarcual.

Linear measurements

As basis of our traditional morphometrics analyses, we used the dataset of Türtscher et al. (2024) [4], who investigated 30 holomorphic specimens representing †*Aellopobatis bavarica*, †*Asterodermus platypterus*, †*Belemnobatis sismondae*, and †*Spathobatis bugesiacus*. As shown by Türtscher et al. (2024) [4], †*Belemnobatis sismondae* can be clearly distinguished from all other taxa both morphologically and morphometrically. The specimen examined here can be clearly distinguished from †*B. sismondae* by its qualitative characters, and we therefore excluded the latter species from our morphometric analyses, resulting in the removal of six specimens belonging to †*B. sismondae* from the original dataset, but added the holotype of the newly described species (DMA-JP-2010/007), resulting in a new dataset containing 25 specimens (Table S1).

We used ImageJ (version 1.53t) to take 27 different measurements (see Fig 1) on each specimen based on photographs to the nearest 0.001 mm. Each measurement was taken three times and the mean values were calculated to reduce measurement errors. Most images included a scale bar with 1 mm increments; for the images without a scale bar, pixels were measured because the subsequent analyses used relative, rather than metric, measurements. The measurements taken were adjusted as percentages of the disc width (% DW) of each individual for traditional morphometric analysis (see below) as well as for species comparison (see 'Systematic Paleontology' section below). In addition, a second traditional morphometric analysis was performed, with measurements adjusted as percentages of the disc length (% DL) of each individual (see Supplemental Material 1). Instead of using the percentage of the total length for species comparison, we decided to use the percentages of the disc width and disc length for two reasons: (1) several specimens are not completely preserved, so total length could not be measured, and (2) completely preserved specimens often do not lie straight but are bent, and measuring the fossil with the tail not straightened can lead to measurement errors. However, the measurements taken from specimen DMA-JP-2010/007 were additionally adjusted as percentages of the total length (% TL) for the species description (see Systematic Paleontology section below).

Fig 1. Illustrations of DMA-JP-2010/007 showing the measurements used for traditional morphometric analyses. Note that the illustrations are mirror images of the original fossil. Abbreviations: DL, disc length; DW, disc width; HDW, half disc width; HL, head length; JW, jaw width; LBAS, length of basipterygia; LMET, length of metapterygia; MAXR, maximum rostrum width; MAXWMES, maximum width of

mesopterygia; MDBAS, inner maximum distance between basipterygia; MDBASO, outer maximum distance between basipterygia; MDMET, inner maximum distance between metapterygia; MDMETO, outer maximum distance between metapterygia; MINR, minimum rostrum width; MWBAS, maximum width of basipterygia; MWMET, maximum width of metapterygia; NC, nasal capsules maximum width; PCGW, pectoral girdle width; PCPV, pectoral girdle to pelvic girdle; PVCF, pelvic girdle to caudal fin tip; PVGW, pelvic girdle width; PVL, pelvic fin length; RAD, span between anteriormost fin radials; RL, rostrum length; SMAX, distance from the tip of the snout to the point of maximum disc width; SPV, snout to pelvic girdle; TL, total length.

It was not possible to obtain all measurements from some of the specimens included, e.g., due to incomplete preservation, resulting in our dataset also containing missing data. We first divided the dataset into *a priori* sorted subsets based on the initial qualitative classification of specimens to reduce the noise caused by missing data in subsequent analyses and imputed the missing values of each subset using a regularized iterative PCA algorithm with the function *imputePCA* in the R package *missMDA* (Josse & Husson, 2016 [15]). The subsets with the implemented data were merged into a dataset containing all specimens and subjected to a Principal Component Analysis (PCA).

Several statistical tests were carried out to explore differences between the groups. First, a Shapiro-Wilk test for normal distribution was applied to each measurement. Subsequently, we created subsets including either normally distributed or non-normally distributed relative measurements, respectively. The dataset containing non-normally distributed measurements was further examined

with non-parametric tests. First, a Kruskal-Wallis test was applied to assess the differences of each measurement among groups. A pairwise Wilcoxon rank sum test with Bonferroni corrections for multiple comparisons for the differences between groups was conducted subsequently. The normally distributed measurements were examined for differences with an Analysis of Variance (ANOVA) and a subsequent pairwise comparison.

All analyses were performed using R 3.6.3 (R Core Team, 2013 [16]) and RStudio 1.2.5019 (R Studio Team, 2019 [17]). Plots were created using the R packages *ggplot2* (Wickham, 2016 [18]) and *viridisLite* (Garnier, 2018 [19]).

Geometric morphometrics

Head outline

As basis of our analyses, we used the dataset of Türtscher et al. (2024) [4], who investigated 21 holomorphic specimens containing the species †*Aellopobatis bavarica*, †*Asterodermus platypterus*, †*Belemnobatis sismondae*, †*Kimmerobatis etchesi*, and †*Spathobatis bugesiacus*. †*Belemnobatis sismondae*, which only occurs in the Kimmeridgian of Cerin (France) was excluded from the present study (see above), but we added two new specimens of †*Aellopobatis bavarica,* one new specimen of †*Asterodermus platypterus*, one new specimen of †*Spathobatis bugesiacus*, and the only specimen of the new taxon to the data matrix (see Table S1).

In total, we performed 2D landmark-based geometric morphometrics on 22 specimens (Table S1). Five homologous landmarks were digitized using the software

tpsDIG2 (v. 2.31; Rohlf, 2017 [20]). Additionally, 36 semilandmarks were digitized between the homologous landmarks to capture the overall shape of the cranial region (Fig 2A). A Generalized Procrustes Analysis (GPA) was performed on the landmark coordinates to minimize the variance caused by factors such as size, orientation, location, and rotation (Rohlf & Slice, 1990 [21]). For minimization of the bending energy, the semilandmarks were allowed to slide (Bookstein, 1997 [22]). Subsequently, the aligned coordinates were subjected to a Principal Component Analysis (PCA) to assess the shape variation of the snouts of the specimens. Shape and size differences between the groups were estimated with a Procrustes ANOVA with 10.000 permutations, followed by pairwise comparisons between the groups, with the functions *procD.lm* and *pairwise* considering the distances between means in the R packages *geomorph* (v. 4.0.4; Adams et al., 2016 [23]) and *RRPP* (Collyer & Adams, 2018 [24]).

Complete body

This analysis included 10 specimens that met the following criteria: very good preservation of the body outline from the tip of the snout to the posterior end of the pelvic fins, the pelvic girdle, the point of attachment of the rostral cartilage to the neurocranium, and the internal boundaries of the pectoral fins from the anterior end to the point of articulation of the propterygium with the scapulocoracoid (Table S1). Eleven homologous landmarks were digitized using the software tpsDIG2 (v. 2.31; Rohlf, 2017 [20]). Additionally, 136 semilandmarks were digitized between the homologous landmarks to capture the overall shape of the body outline, the pelvic girdle, and the anterior internal pectoral fin boundaries (Fig 2B).

A GPA was performed on the landmark coordinates, and the semilandmarks were allowed to slide. The aligned coordinates were then subjected to a PCA. Shape and size differences between the groups were estimated with a Procrustes ANOVA with 10.000 permutations, followed by pairwise comparisons between the groups, with the functions *procD.lm* and *pairwise* considering the distances between means in the R packages *geomorph* (v. 4.0.4; Adams et al., 2016 [23]) and *RRPP* (Collyer & Adams, 2018 [24]).

PLEASE CONSIDER DESCRIBING THE LANDMARKS IN A PARAGRAPH IN THE METHODS SECTION RATHER THAN ONLY IN THIS CAPTION

Fig 2. Location of the true landmarks (large dots in dark grey) and semilandmarks (small dots in light grey) for the two geometric morphometric analyses. A) Head-outline. The true landmarks (1) and (4) are located at the point where the extension of the first radial of the propterygium reaches the edge of the disc, on the right and left pectoral fin respectively; (2) and (3) are located at the notches that indicate the connection between the base of the rostrum and the nasal capsules; (5) is located at the tip of the rostrum. The semilandmarks are arranged in two curves of 18 points each, one between landmarks (1) and (5) and one between landmarks (5) and (4). These two curves describe the shape of the head from the tip of the snout to the first pectoral radial. B) Complete body. True landmark (1) is located at the tip of the snout; (2) and (3) are located at the point where the extension of the first radial of the propterygium reaches the edge of the disc, on the right and left pectoral fin respectively; (4) and (5) are are located at the point where the pelvic fins join the trunk; (6) and (7) are located at the anterior lateral-most points of the pelvic girdle; (8) and (9) are located at the notches that indicate the connection between the base of the rostrum and the nasal capsules; (10) and (11) are located at each connecting point from the propterygia to the scapulocoracoid. The

semilandmarks are arranged in seven curves; 18 semilandmarks each are located between landmarks (1) and (2) as well as between (1) and (3); 38 semilandmarks each are located between landmarks (2) and (4) as well as between (3) and (5); these curves descirbe the body outline from the tip of the snout to the posterior end of the pelvic fins; eight semilandmarks are located between landmarks (6) and (7), describing the anterior outline of the pelvic girdle; eight semilandmarks each are located between landmarks (2) and (10) as well as between (3) and (11).

Remarks

It should be emphasized that statistical analyses involving groups consisting of a single specimen only serve to support the results and interpretations and must be taken with caution due to the limited sample size. In order to obtain robust statistical results, ideally several specimens of a group should be analyzed, which is unfortunately not possible at this time for the two Late Jurassic batomorph taxa †*Apolithabatis seioma* gen. et sp. nov. and †*Kimmerobatis etchesi* due to the small number of known specimens.

Phylogenetic Analyses

For phylogenetic analyses, we adopted and modified the character matrix of Villalobos-Segura et al. (2022) [25]. To identify potential rogue taxa in the original dataset, an initial phylogenetic analysis was conducted on the unmodified data set of Villalobos-Segura et al. (2022) [25] in TNT 1.6 on MacOS (Goloboff et al., 2008 [26]; Goloboff & Morales 2023 [27]) employing the *chkmoves* algorithm. The following

taxa were identified as rogue taxa in the original dataset: *Aptychotrema*, *Bathyraja*, †*Cobelodus*, †*Cyclobatis*, †*Eorhinobatos*, *Glaucostegus*, *Hypanus*, †*Iansan*, †*Lessiniabatis*, †*Ozarcus*, †*Ostarriraja*, *Pseudobatos*, †*Pseudorhinobatos*, *Raja*, *Rhinobatos*, †'*Rhinobatos*' *hakelensis*, †'*R.*' *whitfieldi*, †*Rhombopterygia*, †*Stahlraja*, †*Tlalocbatus*, and *Urobatis*. For subsequent analyses, all fossil ingroup rogue taxa were removed, while extant and outgroup rogue taxa were retained. The rationale behind this was to establish a balance between comprehensive taxon and outgroup sampling and reducing the risk of compromising phylogenetic accuracy by excluding taxa that were not relevant to our analyses. In addition, the Jurassic shark †*Protospinax annectans* was excluded from subsequent analyses due to its ambiguous phylogenetic position (see Jambura et al., 2023 [28]). The character states for the Jurassic batomorphs †*Asterodermus platypterus*, †*Belemnobatis sismondae*, and †*Spathobatis bugesiacus* were revised based on the latest morphological description of these taxa by Türtscher et al. (2024) [4]. In addition, the Jurassic rays †*Aellopobatis bavarica* and the newly described species †*Apolithabatis seioma* gen. et sp. nov. were included in the data matrix. The final version of the modified character matrix contained 76 taxa (44 extant and 32 fossil taxa) and 142 morphological characters, and was compiled in Mesquite 3.81 (Maddison & Maddison, 2023 [29]; Supplemental Material 2).

A parsimony analysis was conducted on the modified dataset using the command-line version of TNT 1.6 on MacOS (Goloboff et al., 2008 [26]; Goloboff & Morales, 2023 [27]). A traditional (heuristic) search was performed with 10.000 replicates of random stepwise addition, tree bisection and reconnection (TBR) branch swapping, and 100 trees stored per replicate. All most parsimonious trees

(MPT) were used to compute consensus trees. Bootstrap and jackknife frequencies were calculated from 1.000 replicates under a traditional search with default settings.

A maximum likelihood (ML) analysis was conducted using the free online phylogenetic tool W-IQ-TREE (Trifinopoulos et al., 2016 [30]). W-IQ-TREE utilizes a dedicated computer cluster at the University of Vienna and is based on the latest version of IQ-TREE (Nguyen et al., 2015 [31]). Morphology was set as the sequence type, and the substitution model was automatically determined by the built-in ModelFinder (Kalyaanamoorthy et al., 2017 [32]). Branch support was calculated from 1.000 replicates using ultrafast bootstrapping (Hoang et al., 2018 [33]).

Log files for all analyses are available in Supplemental Material 2.

Systematic palaeontology

Class CHONDRICHTHYES Huxley, 1880 [34]

Subclass ELASMOBRANCHII Bonaparte, 1838 [35]

Cohort EUSELACHII Hay, 1902 [36]

Subcohort NEOSELACHII Compagno, 1977 [37]

Superorder BATOMORPHII Cappetta, 1980 [38]

†APOLITHABATIFORMES ord. nov.

Nomenclatural remarks

Late Jurassic holomorphic batomorphs show a surprising resemblance to modern Rhinopristiformes, especially to present-day guitarfishes (Rhinobatidae, Glaucostegidae) and wedgefishes (Rhinidae). Due to this superficial resemblance, they traditionally were assigned to the family Rhinobatidae (e.g., Zittel, 1887-1890 [39]; Woodward, 1889a [40]; 1889b [41]; Thies, 1995 [42]; Underwood & Ward, 2004 [43]; Kriwet et al., 2009 [44]; Thies & Leidner, 2011 [45]; Klug & Kriwet, 2013 [46]). After Dames (1888) [47] first introduced the family †Spathobatidae, it was essentially forgotten until Underwood (2006) [6] used the family to include all genera of Late Jurassic batomorphs known from holomorphic fossils, but without specifying to which order this family belongs, since there was little evidence at that time of its position relative to other clades. Recent phylogenetic studies based on morphological characters (Villalobos-Segura et al., 2019 [48]; 2022 [25]; this study [see 'Phylogenetic analysis' below]) and on a combination of molecular and morphological data (Jambura et al., 2023 [28]) posited that Late Jurassic batomorphs form a monophyletic group when maximum parsimony was used as optimality criterion. We follow this hypothesis here, although it should be noted that analyses of the same data sets with different optimality criteria (i.e., maximum likelihood and Bayesian inference) resulted in conflicting topologies and do not support a monophyletic group of Late Jurassic batomorphs (see Villalobos-Segura & Underwood, 2020 [49]: Fig 1; Villalobos-Segura et al., 2022 [25]: Figs 50 and 51; Jambura et al., 2023 [28]: Fig S2). According to the hypothesis accepted here, these batomorphs are sister to all remaining batomorphs and thus cannot be assigned to the family Rhinobatidae.

The monophyletic arrangement of these Late Jurassic batomorphs is only defined by few synapomorphies, but these characters support unambiguously their monophyly. The most important character uniting this group is the mesopterygium,

which is elongated, follows the outline of the propterygium anteriorly to some extent, and thus is relatively large and similar in shape to the propterygium (see Villalobos-Segura et al., 2019 [48] [character 93], Villalobos-Segura et al., 2022 [25] [character 106], Jambura et al., 2023 [28] [character 110], and this study [character 81]). A character that is only found in Rhinopristiformes is the posterolateral dorsal connection of the antorbital cartilage with the nasal capsule, which is positioned, conversely, lateral in Late Jurassic batomorphs (see Villalobos-Segura et al., 2022 [25] [character 110], de Carvalho, 2004 [50] [character 2], Jambura et al., 2023 [28] [character 24], and this study [character 20]). Additionally, Late Jurassic taxa are the only members of Batomorphii (other than the undescribed batomorph from the Toarcian of Holzmaden, Germany) in which true dorsal fin spines are present (although absent in some species), thus showing retention of a plesiomorphic chondrichthyan character absent in crown-group batomorphs (see Villalobos-Segura et al., 2022 [25] [character 130], Jambura et al., 2023 [28] [character 177], and this study [character 134]).

We consequently propose a new order, †Apolithabatiformes ord. nov., which includes the single family, †Spathobatidae with the genera †*Aellopobatis,* †*Apolithabatis* gen. nov., †*Asterodermus*, †*Belemnobatis*, †*Kimmerobatis*, and †*Spathobatis*. This order represents the most plesiomorphic clade within Batomorphii being placed on the stem of the total group Batomorphii. It is possible that the hitherto undescribed Late Jurassic batomorph from Argentina, previously considered merely as Batomorphii indet. by Cione (1999) [13], also belongs to this order, which, however, can only be clarified by a detailed examination of the specimen.

* PLEASE CONSIDER INSERTING A PARAGRAPH WITH A LIST OF THE DIAGNOSTIC CHARACTERS OF THE NEW ORDER, JUST AS A SUMMARY

Type Species

†*Apolithabatis seioma* gen. et sp. Nov.

Included taxa

†*Aellopobatis bavarica,* †*Apolithabatis seioma* gen. et sp. nov., †*Asterodermus platypterus*, †*Belemnobatis* spp., †*Kimmerobatis etchesi*, †*Spathobatis* spp.

Etymology

The name ‘Apolithabatiformes’ is composed of two Greek words, i.e., ‘απολίθωμα’ (apolíthoma) meaning ‘fossil’ and ‘βατίς‘ (batís) meaning ‘ray‘ or ‘skate‘.

*

Family †SPATHOBATIDAE Dames, 1888 [47]

†*APOLITHABATIS* gen. nov.

Type Species

†*Apolithabatis seioma* gen. et sp. nov.

Included taxa

Type species only.

Etymology

Identical to that of the order †Apolithabatiformes (see above): the genus name '*Apolithabatis*' is composed of two Greek words, i.e., ‘Απολίθωμα’ (apolíthoma) meaning ‘fossil’, and ‘βατίς‘ (batís) meaning ‘ray‘ or ‘skate‘.

Stratigraphic and geographic distribution

Only known from the upper Kimmeridgian (Upper Jurassic) of the ‘Solnhofen Archipelago’ (Painten), Bavaria, Germany.

Diagnosis

A ‘guitarfish’-like batomorph unique in having the following combination of characters: heart-shaped disc that is wider than long; pointed snout; antorbital cartilages present but reaching less than halfway between the nasal capsules and the propterygium; vertebral centra extending less than half of the synarcual length; large mesopterygium tangent to the propterygium; 40 pectoral radials (9 propterygial, 11 mesopterygial, 20 metapterygial); no pectoral radials articulate directly with the scapulocoracoid between the meso- and metapterygium; pectoral radials segmented in up to five segments; at least 16 pairs of ribs; 19 basipterygial radials (including one compound radial); puboischiadic bar curved anteriorly; no postpelvic processes present; broad and triangular lateral prepelvic processes; well-developed and plate-

like hemal and supraneural spines; conspicuous bulge-like structure formed by the supraneural spines in front of each dorsal fin; no fin spines present.

†*APOLITHABATIS SEIOMA* gen. et sp. nov.

(Figs 3, 4, 5, 6, S1)

https://zoobank.org/nomenclaturalacts/XXX

Fig 3. Overview of DMA-JP-2010/007, the holotype of †*Apolithabatis seioma* gen. et sp. nov. A) photograph of the specimen. B) Illustration of the specimen showing the skeletal morphology. Abbreviations: ac, antorbital cartilage; bp, basipterygium; br, branchial arches; c, vertebral centra; cf, caudal fin; d1, first dorsal fin; d2, second dorsal fin; hs, hemal spine; mk, Meckel's cartilage, ms, mesopterygium; mt, metapterygium; nc, nasal capsule; ns, neural spine; pb, puboischiadic bar; pp, propterygium; pq, palatoquadarte; r, ribs; ra, pectoral fin radials; rap, pelvic fin radials; ro, rostrum; sc, scapulocoracoid; syn, synarcual. The scale bar equals 10 cm.

Fig 4. Head region of DMA-JP-2010/007. A) Close-up of the head region with several skeletal structures outlined. Abbreviations: ac, antorbital cartilage; mk, Meckel's cartilage, nc, nasal capsule; pq, palatoquadarte; ro, rostrum. B) Close-up of the head region under ultraviolet light. C) Close-up of the nasal capsules and the jaw

cartilages under normal light and D) under ultraviolet light. E-F) Close-up of the teeth. Scale bars: A-B) 5 cm, C-D) 2 cm, E-F) 0.5 cm.

Fig 5. Left pectoral fin of DMA-JP-2010/007. A) Close-up of the left pecotral fin with the basal cartilages outlined. Abbreviations: ms, mesopterygium; mt, metapterygium; pp, propterygium. B) Close-up under ultraviolet light. Scale bars equal 5 cm.

Fig 6. Pelvic region of DMA-JP-2010/007. A) Close-up with several skeletal structures outlined. Abbreviations: bp, basipterygium; cr, compound radial; hs, hemal spine; ns, neural spine; r, ribs; rap, pelvic fin radials. B) Close-up under ultraviolet light. Scale bars equal 5 cm.

Etymology

The species name '*seioma*' is derived from the Greek 'σείω' (seíǫ), a verb stemming from the noun 'σεισμός' (seismós), meaning 'shake' or 'jiggle', in reference to the blasting of the fossil from the rock.

Holotype

DMA-JP-2010/007

Referred Material

Only known from the holotype (DMA-JP-2010/007).

Locality

Quarry of Rygol Company near the village of Painten, Lower Bavaria, South Germany.

Diagnosis

As for the genus.

Description

Body shape

Large-sized batomorph, reaching a total length of at least 120 cm. The body shape is 'guitarfish'-like. The disc is large and heart-shaped, reaching a width of 48.4% of the total length. With a length of 47.2% TL, the disc is slightly wider than long. The tail is narrow and long and accounts for 61.3% TL, measured from the pelvic girdle to the tip of the caudal fin. The two dorsal fins are located well posterior to the pectoral girdle; the first dorsal fin originates at 63.3% TL, the second dorsal fin at 73.9% TL. Both dorsal fins are nearly equal in size, the first dorsal fin has a height of 4.8% TL and a width of 5.3% TL, the second dorsal fin has a height of 5.2% TL and a width of 5.6% TL (Figs 3 and S1).

Neurocranium

The neurocranium of DMA-JP-2010/007 is partly crushed and details posterior to the nasal capsules are difficult to discern. The rostrum is quite robust and tapers slightly in mid-length. The anterior-most portion of the rostral cartilage is covered with skin, but apparently extends to the tip of the snout. As such, the rostrum is longer than the remaining part of the neurocranium. The nasal capsules are almost oval and inclined anteriorly. The width of the nasal capsules and the jaw cartilages is almost identical. The antorbital cartilages are elongated with an indentation in the mid-region of the anterior edge. The connection to the postero-lateral parts of the nasal capsules is rather narrow. The antorbital cartilages reach less than half the distance between the nasal capsules and the propterygia (Fig 4A-B).

Jaws and branchial skeleton

Large parts of the central part of the jaws are fractured, thus their complete shape is not recognizable. However, the distal parts, particularly the right distal part, are preserved. A small dorsal flange of the Meckel’s cartilage hooking around the posterior part of the palatoquadrate is discernible. Both jaw cartilages are almost straight and equal in antero-posterior depth. Due to taphonomic damage, structures such as the basihyals and basibranchials are not clearly discernable. Anteriorly, the branchial arches are approximately as wide as the jaws, but the width of the branchial apparatus tapers toward the pectoral girdle. The ceratobranchials are long and narrow (Fig 4A-D).

Pectoral girdle and fins

The scapulocoracoid is broken in the middle, with the right side still in its natural position and the left side displaced upwards. All three basal cartilages articulate directly with the scapulocoracoid. The propterygium is broad and unsegmented. The mesopterygium is large, oval shaped, and adjacent to the propterygium for almost its entire length. The metapterygium is strongly curved and very broad in the first half, tapering increasingly in the second half. Of the three basal cartilages, the propterygium has the smallest articulation with the scapulocoracoid and the mesopterygium has the widest. In total, 40 radials articulate with the basal cartilages (9 with the propterygium, 11 with the mesopterygium, and 20 with the metapterygium). No radial articulates directly with the scapulocoracoid. At the widest part of the disc, the radials are composed of five elements (Fig 5A-B).

Axial skeleton and unpaired fins

The vertebral column consists of cyclospondylic centra that gradually decrease in size along the body. The exact outline of the synarcual is obscure due to taphonomic damage to the head region, but it appears to be broad at the insertion to the neurocranium, tapers in the middle, then widens and forms distinct lateral stays, and then tapers again. The vertebral centra extend less than half of the length of the synarcual. Sixteen pairs of ribs are present. No fin spines are associated with the dorsal fins. The well-developed and plate-like haemal spines seem to appear just after the last pair of ribs and are present throughout the tail. While the specimen is preserved in ventral view, the tail is rotated around its longitudinal axis, best seen

NOT CONSISTENT SPELLING IN CAPTIONS AND ABBREVIATIONS

caudally of the second dorsal fin by a prominent fold. The bases of the dorsal fins (as well as that of the caudal fin) are thus somewhat obscured and not fully visible. The dorsal fins appear to have been flipped over first, followed by the rigid spine because of taphonomic processes, resulting in the supraneural spines piercing the already decomposing skin and the flipped dorsal fins becoming 'stuck' between supraneural spines and the folded skin on the right side of the tail. The supraneural spines, only visible in the tail, are well-developed and plate-like. Just anterior to each dorsal fin, the supraneural spines form a bulge-like structure. Since there are no traces of possible fin spines, we interpret this structure as a fixation point of connective tissue, acting as a reinforcement of the leading edge. In the living animal, these structures may well have been positioned directly at the base of each dorsal fin, but since the skin appears to have detached and shifted after the animal's death, it is possible that the dorsal fins are slightly displaced caudally (Fig 6A-B).

CONSIDER DIVIDING THE SENTENCES, IT IS QUITE COMPLEX

Pelvic girdle, fins, and claspers

Specimen DMA-JP-2010/007 is female, therefore no claspers can be observed. The pelvic fins reach 15.6% TL and are more than twice as long as wide. The basipterygium is slightly curved and narrow. One large and robust compound radial as well as 18 radials articulate with the basipterygium. The puboischiadic bar is not straight but curved anteriorly; in the middle it shows an additional incurvation. The lateral prepelvic processes are very broad and triangular (Fig 6A-B).

Teeth and denticles

The anterior teeth are wider mesiodistally than labio-lingually deep. A mesiodistal ridge separates the labial face from the lingual face of the crown. An apron is absent or highly reduced and barely detached from the crown. The uvula is narrow and long (Fig 4E-F).

Comparison

The snout has no knob-like or paddle-shaped projection, contrary to the condition seen in †*Ae. bavarica,* †*As. platypterus*, and †*S. bugesiacus*, but similar to what is seen in †*K. etchesi*. The antorbital cartilages reach less than halfway the distance between the nasal capsules and the propterygium, similar to the condition in †*Ae. bavarica*, †*S. bugesiacus*, and †*K. etchesi* (in †*As. platypterus*, the antorbital cartilages extend [usually less than halfway, but] up to halfway between the nasal capsules and the propterygium). The antorbital cartilages have straight posterior and anterior margins with an indentation in the middle, contrary to what is developed in †*Ae. bavarica* [both margins straight], †*As. platypterus* [no indentations but a posteriorly curved shape], and †*K. etchesi* [triangular in shape].; In the new taxon, no pectoral radials articulate directly with the scapulocoracoid between the meso- and metapterygium, which is different in †*S. bugesiacus*, but resembles the condition in †*Ae. bavarica*, †*As. platypterus*, and †*K. etchesi*. Conversely to †*As. platypterus* and †*S. bugesiacus*, †*Ap. seioma* gen. et sp. nov. has no fin spines preceeding the dorsal fins, which is also the case in †*Ae. bavarica*.

Results

Traditional morphometrics

General

Using the new data set (Fig 1, Table S1), we performed a Principal Component Analysis considering all measurements that resulted in 25 axes (Table S2), with the first four each explaining more than 5% of the variation and together accounting for 77.89% of the total variability (see Table S3 for loading values). The most important variables for PC1 and PC2 are described in detail later in this section (see below). The occupied morphospace plotted on the first two axes and the associated variables are shown in Fig 7.

Fig 7. Traditional morphometrics: results of the principal component analysis (PCA), with each measurement adjusted to percentage of the disc width (DW) of each individual. A) morphospace plotted on PC1 (38.3% of the total variance) and PC2 (24.44%). Asterisks indicate the holotype of the respective species. B) loading values showing the variables associated with the first two PC axes. Asterisks indicate the variables that explain the most variation for the respective PC axes. Abbreviations: DL, disc length; HDW, half disc width; HL, head length; JW, jaw width; LBAS, length of basipterygia; LMET, length of metapterygia; MAXR, maximum rostrum width; MAXWMES, maximum width of mesopterygia; MDBAS, inner maximum distance between basipterygia; MDBASO, outer maximum distance between basipterygia; MDMET, inner maximum distance between metapterygia; MDMETO, outer maximum distance between metapterygia; MINR, minimum rostrum

width; MWBAS, maximum width of basipterygia; MWMET, maximum width of metapterygia; NC, nasal capsules maximum width; PCGW, pectoral girdle width; PCPV, pectoral girdle to pelvic girdle; PVCF, pelvic girdle to caudal fin tip; PVGW, pelvic girdle width; PVL, pelvic fin length; RAD, span between anteriormost fin radials; RL, rostrum length; SMAX, distance from the tip of the snout to the point of maximum disc width; SPV, snout to pelvic girdle; TL, total length.

Along PC1 (38.3% of the variation), †*Ae. bavarica*, †*As. platypterus*, and †*S. bugesiacus* are well separated, while †*Ap. seioma* gen. et sp. nov. overlaps with †*Ae. bavarica*. The occupied morphospaces of †*As. platypterus* and †*S. bugesiacus* are both in the positive range of PC1, while those of †*Ae. bavarica* and †*Ap. seioma* gen. et sp. nov. are in the negative range. This is also reflected in the most important variables of PC1, which mostly overlap in †*Ae. bavarica* and †*Ap. seioma* gen. et sp. nov., as well as in †*As. platypterus* and †*S. bugesiacus* (see below; see also Fig 7B and Fig 8). Along PC2 (24.44% of the variation), †*Ae. bavarica* and †*Ap. seioma* gen. et sp. nov. can be clearly distinguished. †*Asterodermus platypterus* overlaps slightly with †*Ae. bavarica*, and †*S. bugesiacus* overlaps with both †*Ae. bavarica* and †*As. platypterus*. †*Apolithabatis seioma* gen. et sp. nov. does not overlap with any other occupied morphospace along PC2. Both †*Ae. bavarica* and †*S. bugesiacus* are predominantly in the positive range of PC2, †*As. platypterus* is almost entirely in the negative range, and †*Ap. seioma* gen. et sp. nov. is located in the negative morphospace of PC2. Within the main variables of PC2, †*Ap. seioma* gen. et sp. nov. overlaps only slightly with the other taxa, but separates most clearly from †*Ae. bavarica* (see below; see also Fig 7B and Fig 8). The morphospaces plotted on PC1

and PC3 (8.61% of the total variation) as well as on PC1 and PC4 (6.54% of the variation) along with the associated variables are shown in Fig S2.

Fig 8. Traditional morphometrics: boxplots of the variables explaining the most variation of PC1 and PC2, respectively. Measurements are adjusted as percentage of the disc width (DW). Abbreviations: DL, disc length; HL, head length; JW, jaw width; LMET, length of metapterygia; MAXWMES, maximum width of mesopterygia; MDBAS, inner maximum distance between basipterygia; MDBASO, outer maximum distance between basipterygia; MDMET, inner maximum distance between metapterygia; MDMETO, outer maximum distance between metapterygia; NC, nasal capsules maximum width; PCGW, pectoral girdle width; PCPV, pectoral girdle to pelvic girdle; PVCF, pelvic girdle to caudal fin tip; PVGW, pelvic girdle width; PVL, pelvic fin length; RAD, span between anteriormost fin radials; RL, rostrum length; SMAX, distance from the tip of the snout to the point of maximum disc width; SPV, snout to pelvic girdle; TL, total length.

The results of the Shapiro-Wilk normality test showed that *ca.* 84.62% of all measurements are normally distributed (i.e., 22 out of 26 measurements; Table S4). The non-normally distributed measurements were further analyzed with the Kruskal–Wallis rank sum test, which indicates significant differences among the taxa for all but one measurement (i.e., total length; Table S5), which is further supported by Wilcoxon pairwise comparisons between the taxa (Table S6).

ANOVA tests on each normally distributed measurement showed significant differences in all but three measurements (i.e., maximum rostrum width, minimum

rostrum width, pelvic girdle to caudal fin tip, maximum width of basipterygia, length of basipterygia, maximum width of mesopterygium, half disc width; Table S7), which was also largely confirmed by the Tukey test for pairwise comparisons (Table S8).

Strongest vectors for PC1

The following measurements were identified as having the strongest impact along PC1 and are compared interspecifically below: nasal capsules maximum width (NC), jaw width (JW), pectoral girdle width (PCGW), pelvic girdle width (PVGW), pectoral girdle to pelvic girdle (PCPV), length of metapterygia (LMET), maximum outer distance between metapterygia (MDMETO), maximum inner distance between metapterygia (MDMET), maximum outer distance between basipterygia (MDBASO), maximum inner distance between basipterygia (MDBAS), and span between last fin radials (RAD).

The nasal capsule maximum width (NC) amounts to 24.2% DW in †*Ap. seioma* gen. et sp. nov. and ranges from 20.7 to 28.9% DW in †*Ae. bavarica*, from 27.8 to 30.6% DW in †*As. platypterus*, and from 28.8 to 32.9% DW in †*S. bugesiacus*. Statistically significant differences were found between †*Ae. bavarica* and †*S. bugesiacus* (Table S6, Fig 8).

Jaw width (JW) is 25.1% DW in †*Ap. seioma* gen. et sp. nov. and ranges from 24.1 to 27.7% DW in †*Ae. bavarica*, from 27.0 to 29.1% DW in †*As. platypterus*, and from 30.1 to 33.9% DW in †*S. bugesiacus*. The pairwise comparison shows that †*Ae. bavarica,* †*As. platypterus*, and †*S. bugesiacus* are all significantly different to each other in this relative measurement (Table S6, Fig 8).

The pectoral girdle width (PCGW) accounts for 33.8% DW in †*Ap. seioma* gen. et sp. nov. and ranges from 28.7 to 33.6% DW in †*Ae. bavarica,* from 32.3 to 37.3% DW in †*As. platypterus*, and from 37.9 to 40.7% DW in †*S. bugesiacus*. Statistically relevant differences were found between all taxa except between †*Ap. seioma* gen. et sp. nov. and †*Ae. bavarica* as well as †*As. platypterus* (Table S8, Fig 8).

The pelvic girdle width (PVGW) is 26.9% DW in †*Ap. seioma* gen. et sp. nov. and spans from 23.9 to 28.4% DW in †*Ae. bavarica*, from 27.1 to 31.3% DW in †*As. platypterus*, and from 29.4 to 34.8% DW in †*S. bugesiacus*. The largest differences were found between †*Ae. bavarica* and †*S. bugesiacus*, †*Ae. bavarica* and †*As. platypterus*, and between †*Ap. seioma* gen. et sp. nov. and †*S. bugesiacus* (Table S8, Fig 8).

The distance from the pectoral girdle to the pelvic girdle (PCPV) is 18.3% DW in †*Ap. seioma* gen. et sp. nov. and spans from 19.8 to 26.7% DW in †*Ae. bavarica*, from 26.1 to 29.4% DW in †*As. platypterus*, and from 28.1 to 31.9% DW in †*S. bugesiacus*. With the exception of †*Ae. bavarica* to †*Ap. seioma* gen. et sp. nov. and †*As. platypterus* to †*S. bugesiacus*, all taxa are statistically significantly different from each other (Table S8, Fig 8).

The length of the metapterygia (LMET) is 18.2% DW in †*Ap. seioma* gen. et sp. nov. and ranges from 18.3 to 24.2% DW in †*Ae. bavarica*, from 21.6 to 26.5% DW in †*As. platypterus*, and from 20.6 to 25.3% DW in †*S. bugesiacus*. No statistically significant differences were detected between the groups (Table S8, Fig 8).

The maximum outer distance between the metapterygia (MDMETO) amounts to 47.5% DW in †*Ap. seioma* gen. et sp. nov. and ranges from 43.6 to 48.2% DW in

†*Ae. bavarica*, from 45.3 to 55.7% DW in †*As. platypterus*, and from 50.1 to 54.4% DW in †*S. bugesiacus*. Similarly, the maximum inner distance between the metapterygia (MDMET) amounts to 39.9% DW in †*Ap. seioma* gen. et sp. nov. and ranges from 34.9 to 40.1% DW in †*Ae. bavarica*, from 37.7 to 48.3% DW in †*As. platypterus*, and from 43.2 to 47.5% DW in †*S. bugesiacus*. Both pairwise comparisons reveal significant differences between †*Ae. bavarica* and †*As. platypterus* as well as between †*Ae. bavarica* and †*S. bugesiacus* (Table S8, Fig 8).

The maximal outer distance between basipterygia (MDBASO) accounts for 23.2% DW in †*Ap. seioma* gen. et sp. nov. and ranges from 22.4 to 29.2% DW in †*Ae. bavarica*, from 27.5 to 29.7% DW in †*As. platypterus*, and from 29.5 to 31.5% DW in †*S. bugesiacus*. With the exception of †*Ap. seioma* gen. et sp. nov., all groups are statistically significantly different from each other (Table S6, Fig 8).

The intraspecific range of the maximum inner distance between basipterygia (MDBAS) is similar to the maximal outer distance between basipterygia (MDBASO); it amounts to 19.9% DW in †*Ap. seioma* gen. et sp. nov. and ranges from 18.3 to 24.2% DW in †*Ae. bavarica*, from 23.9 to 25.9% DW in †*As. platypterus*, and from 24.4 to 27.6% DW in †*S. bugesiacus*. However, unlike the results for MDBASO, the pairwise comparison reveals significant differences between all taxa except between †*Ae. bavarica* and †*Ap. seioma* gen. et sp. nov., as well as between †*As. platypterus* and †*S. bugesiacus* (Table S8, Fig 8).

The span between last fin radials (RAD) is 47.4% DW in †*Ap. seioma* gen. et sp. nov. and ranges from 42.2 to 55.4% DW in †*Ae. bavarica*, from 47.5 to 63.4% DW in †*As. platypterus*, and from 53.8 to 56.7% DW in †*S. bugesiacus*. †*Aellopobatis bavarica* differs significantly from †*As. platypterus* as well as from †*S. bugesiacus* (Table S8, Fig 8).

Strongest vectors for PC2

The following measurements were identified as having the greatest impact along PC2 and are compared interspecifically below: maximum width of mesopterygium (MAXWMES), head length (HL), snout to maximum disc width (SMAX), disc length (DL), total length (TL), rostrum length (RL), pelvic-fin length (PVL), pelvic girdle to caudal fin tip (PVCF), and snout to pelvic girdle (SPV).

The maximum width of the mesopterygium (MAXWMES) measures 11.8% DW in †*Ap. seioma* gen. et sp. nov. and ranges from 8.9 to 13.7% DW in †*Ae. bavarica*, from 12.2 to 14.8% DW in †*As. platypterus*, and from 11.4 to 13.3% DW in †*S. bugesiacus*. The taxa are not statistically different (Table S8, Fig 8).

The head length (HL) is 58.9% DW in †*Ap. seioma* gen. et sp. nov. and ranges from 71.9 to 85.6% DW in †*Ae. bavarica*, from 63.5 to 73.2% DW in †*As. platypterus*, and from 71.2 to 77.7% DW in †*S. bugesiacus*. With the exception of †*Ae. bavarica* to †*S. bugesiacus* and †*As. platypterus* to †*Ap. seioma* gen. et sp. nov., all taxa are significantly different to each other (Table S8, Fig 8).

The distance from the tip of the snout to the point of maximum disc width (SMAX) accounts for 66.4% DW in †*Ap. seioma* gen. et sp. nov. and ranges from 77.8 to 96.6% DW in †*Ae. bavarica*, from 66.5 to 79.3% DW in †*As. platypterus*, and from 78.3 to 86.8% DW in †*S. bugesiacus.* With the exception of †*Ae. bavarica* to †*S. bugesiacus* and †*As. platypterus* to †*Ap. seioma* gen. et sp. nov., all taxa are significantly different to each other (Table S8, Fig 8).

The disc length (DL) is 97.6% DW in †*Ap. seioma* gen. et sp. nov. and spans from 110.9 to 127.6% DW in †*Ae. bavarica*, from 101.0 to 108.9% DW in †*As. platypterus*, and from 112.6 to 125.1% DW in †*S. bugesiacus*. With the exception of

†*Ae. bavarica* to †*S. bugesiacus* and †*As. platypterus* to †*Ap. seioma* gen. et sp. nov., all taxa are significantly different to each other (Table S8, Fig 8).

The total length (TL) measures 206.7% DW in †*Ap. seioma* gen. et sp. nov. and ranges from 207.2 to 274.4% DW in †*Ae. bavarica*, from 207.3 to 251.1% DW in †*As. platypterus*, and from 238.3 to 282.4% DW in †*S. bugesiacus*. The taxa are not statistically different in this relative measurement (Table S8, Fig 8).

The length of the rostrum (RL) is 26.9% in †*Ap. seioma* gen. et sp. nov. and ranges from 41.2 to 51.7% DW in †*Ae. bavarica*, from 29.7 to 37.1% DW in †*As. platypterus*, and from 31.9 to 40.2% DW in †*S. bugesiacus*. All taxa differ significantly from each other, except †*As. platypterus* from †*Ap. seioma* gen. et sp. nov. and †*As. platypterus* from †*S. bugesiacus* (Table S8, Fig 8).

The length of the pelvic fin (PVL) is 32.3% DW in †*Ap. seioma* gen. et sp. nov. and spans from 31.7 to 44.4% DW in †*Ae. bavarica*, from 28.9 to 32.9% DW in †*As. platypterus*, and from 30.2 to 41.2% DW in †*S. bugesiacus*. Statistically significant differences were found between †*Ae. bavarica* and †*As. platypterus* and between †*As. platypterus* and †*S. bugesiacus* (Table S8, Fig 8).

The distance from the pelvic girdle to the caudal fin tip (PVCF) is 126.7% DW in †*Ap. seioma* gen. et sp. nov. and ranges from 112.7 to 166.6% DW in †*Ae. bavarica*, from 103.2 to 146.9% DW in †*As. platypterus*, and from 137.9 to 141.5% DW in †*S. bugesiacus*. The taxa are not statistically different in this relative measurement (Table S8, Fig 8).

The distance from the tip of the snout to the pelvic girdle (SPV) is 77.2% DW in †*Ap. seioma* gen. et sp. nov. and spans from 91.9 to 111.1% DW in †*Ae. bavarica*, from 89.2 to 101.5% DW in †*As. platypterus*, and from 95.0 to 108.7% DW in †*S.*

bugesiacus. In this relative measurement, †*Ap. seioma* gen. et sp. nov. is statistically different from all other taxa (Table S8, Fig 8).

Geometric morphometrics

Head outline

A PCA was performed on the data matrix (Table S1), resulting in 21 axes, with the first two accounting for 86.98% of the total shape variation (Table S9). The remaining 19 axes each account for <5% of the total variation.

The morphospace occupation of the taxa plotted on PC1 (77.61% of the total morphological variation) and PC2 (9.37% of the variation) shows no overlap between taxa. †*Apolithabatis seioma* gen. et sp. nov. is positioned close to †*As. platypterus*, reflecting the similarly short rostra in comparison to †*Ae. bavarica* and †*S. bugesiacus,* which have elongated and paddle-shaped rostra. Likewise, †*K. etchesi* is located close between †*As. platypterus* and †*S. bugesiacus* on the morphospace (Fig 9).

Fig 9. Geometric morphometrics – head outline: results of the principal component analysis (PCA). Morphospace plotted on PC1 (77.61% of the total variation) and PC2 (9.37%). Asterisks indicate the holotype of the respective species. Mean shapes are shown next to each group. Dark-coloured dots of the mean shapes indicate true landmarks, light-coloured dots indicate semilandmarks.

PC1 describes the length and shape of the rostrum. †*Aellopobatis bavarica*, evidently the taxon with the most distinct rostrum, occupies the negative area of PC1, followed by †*S. bugesiacus*, which spans from negative to positive values of PC1. †*Asterodermus platypterus* is confined to the positive region of PC1. Both †*K. etchesi* and †*Ap. seioma* gen. et sp. nov. have a rather pointed head outline and lack a paddle-shaped rostrum which is reflected by their morphospace occupation in the positive area of PC1. The shape changes along PC2 are far more subtle and mainly comprise the width of the head (Fig 9).

A Procrustes ANOVA reveals significant differences in shape between the taxa (R^2 = 0.67184, F = 8.701, Z = 4.1144, *p* = 0.001; Table S10), which is further supported by a pairwise comparison (Table S11). The pairwise comparison shows that †*Ae. bavarica* is the most distinct taxon, with a significant difference to all others.

The second Procrustes ANOVA indicates significant differences in size between the taxa (R^2 = 0.75743, F = 13.271, Z = 3.0672, *p* = 0.001; Table S12). The pairwise comparison detects significant differences between †*K. etchesi* and †*Ae. bavarica*, †*As. platypterus*, as well as †*S. bugesiacus*. Furthermore, †*Ae. bavarica* and †*As. platypterus* also are significantly different in size (Table S13).

Complete body

Since only a few of the specimens are sufficiently preserved along the entire body to perform this landmark analysis, only 10 specimens were included here (Table S1). To capture the body outline but also the position of different skeletal structures, 11 landmarks and 136 semilandmarks were used on specimens of †*Ae.*

bavarica, †*As. platypterus*, †*Ap. seioma* gen. et sp. nov., and †*S. bugesiacus*. Note the small sample size when interpreting the statistical results of this analysis.

The PCA resulted in 9 axes, with the first five accounting for 88.56% of the total variation. The remaining 4 axes each account for <5% of the total variation (Table S14).

When plotted on PC1 (36.96% of the total variation) and PC2 (23.66%), †*As. platypterus* and †*S. bugesiacus* are relatively close to each other. †*Aellopobatis bavarica* and †*Ap. seioma* gen. et sp. nov., on the other hand, are clearly separated from each other as well as from the other two taxa.

The negative area of PC1 is occupied by †*Ae. bavarica* and †*Ap. seioma* gen. et sp. nov., while †*As. platypterus* and †*S. bugesiacus* are restricted to the positive region of PC1. The negative values are associated with an elongation of the rostrum, resulting in the typical spatula-shaped rostrum seen in †*Ae. bavarica* in the most negative range. Also, the pelvic girdle slightly shifts forward and the pelvic gins become narrower, resulting in a quite slender caudal region. In addition, the base of the nasal capsules shifts slightly backwards and the head region becomes somewhat narrower (Fig 10).

Fig 10. Geometric morphometrics – complete body: results of the principal component analysis (PCA). Morphospace plotted on PC1 (36.96% of the total variation) and PC2 (23.66%). Asterisks indicate the holotype of the respective species. Mean shapes are shown next to each group. Dark-coloured dots of the mean shapes indicate true landmarks, light-coloured dots indicate semilandmarks.

In the positive region of PC1, the changes are reversed with the rostrum becoming shorter, the base of the nasal capsules moving forward, the head region becoming slightly wider, the pelvic girdle moving posteriorly, and the pelvic fins and caudal region increasing in width. The negative region of PC2 is characterized by a tapering of the rostrum. The head has a pointed shape and no spatula-shaped extension of the rostrum is present, neither short as seen in †*As. platypterus* and †*S. bugesiacus* nor prolonged as in †*Ae. bavarica*. In addition, the disc becomes much wider and the pelvic girdle shifts forward. In the positive region of PC2, the rostrum becomes slightly longer and the disc narrower. Fig S3 depicts the morphospaces plotted on PC1 and PC2, PC3 (12.24%), PC4 (9.05%), and PC5 (6.64%), respectively. In all four morphospaces, the four taxa are clearly separated from each other and never overlap.

A Procrustes ANOVA reveals significant differences in shape between the taxa (R^2 = 0.61197, F = 3.1543, Z = 3.5893, *p* = 0.001; Table S15). In the pairwise comparison, statistically significant differences are shown between †*Ae. bavarica* and †*Ap. seioma* gen. et sp. nov., †*Ae. bavarica* and †*As. platypterus*, as well as †*Ap. seioma* gen. et sp. nov.. and †*As. platypterus* (Table S16).

The detected differences in size between the taxa (R^2 = 0.74207, F = 5.7541, Z = 1.889, *p* = 0.043; Table S17) is only supported by statistical significance for †*Ae. bavarica* and †*S. bugesiacus* in the pairwise comparison (Table S18).

Phylogenetic analysis

The parsimony analysis resulted in 1611 most parsimonious trees (MPTs) with a best score of 371 steps. Consistency index (CI) and retention index (RI) of the

MPTs were 0.461 and 0.857, respectively. Both maximum parsimony (MP) and maximum likelihood (ML) analyses indicated a basal phylogenetic placement of †*Ap. seioma* gen. et sp. nov. within the rays (superorder Batomorphii) and a sister relationship between †*Ap. seioma* gen. et sp. nov. and †*Ae. bavarica* (Figs 11 and S6). Both analyses yielded conflicting results for the phylogenetic relationships among Jurassic rays, with the ML analysis providing a better-resolved phylogenetic tree (see Discussion below).

Fig 11. Majority-rule consensus tree with bootstrap and jackknife frequencies (jackknife values in parentheses). Daggers before taxon names indicate extinct taxa. Silhouettes were either drawn by the authors (JT, FS, PLJ) or downloaded from www.phylopic.org (all downloaded images were available for re-use under the Public Domain Dedication 1.0 licence).

Discussion

In the study by Türtscher et al. (2024) [4], five holomorphic Late Jurassic ray taxa unambiguously have been confirmed, i.e. †*Aellopobatis bavarica*, †*Asterodermus platypterus*, †*Belemnobatis sismondae*, †*Kimmerobatis etchesi*, and †*Spathobatis bugesiacus*. †*Spathobatis*? *morinicus* from the lower Tithonian of Canjuers (southern France) may represent another taxon, but needs to be studied and redescribed to confirm its position within †*Spathobatis* and to determine whether it is indeed a separate species or a member of †*Spathobatis bugesiacus*. The sedimentary deposits of Cerin (south-eastern France), where the only skeletal

remains of †*S. bugesiacus* have been found, are of late Kimmeridgian age. Isolated teeth of †*Spathobatis bugesiacus*, however, have been reported from both Kimmeridigan and Tithonian aged outcrops in Europe (Underwood, 2002 [51]; Leuzinger et al., 2017 [52]). The undescribed Argentinean ray (Cione, 1999 [13]) is still awaiting a detailed anatomical and taxonomical description and therefore cannot be confirmed at this stage whether it represents a new genus and/or species, or whether it belongs to one of the known taxa. †*Apolithabatis seioma* gen. et sp. nov. thus represents the sixth undoubtedly valid Late Jurassic ray taxon known from skeletal material, and most probably the first batomorph from the upper Kimmeridgian of the 'Solnhofen Archipelago' (for a palaeoenvironmental reconstruction of †*Apolithabatis seioma* gen. et sp. nov., see Fig 12).

Fig 12. Environmental reconstruction showing †*Apolithabatis seioma* gen. et sp. nov. in association with a juvenile pleurosaurid.

The phylogenetic relationships within batomorphs have long been the subject of intense research, but despite a lot of progress in resolving various taxonomic and systematic issues, countless unanswered questions still persist. Therefore, including Jurassic ray taxa, which belong to the oldest known batomorph species to date, into phylogenetic analyses helps to generate better resolved hypotheses of batomorph evolution (Underwood & Claeson, 2019 [53]). So far, several phylogenetic studies (Underwood & Claeson, 2019 [53]; Villalobos-Segura et al., 2019 [48]; 2022 [25]; Jambura et al., 2023 [28]) indicated that the Late Jurassic rays that are known from holomorphic specimens formed a monophyletic group, here named

Apolithabatiformes ord. nov. In the phylogenetic study of Underwood and Claeson (2019) [53], these Late Jurassic batomorphs were placed within the crown-group batomorphs at the base of Rhinopristiformes, Myliobatiformes, Rajiformes, and platyrhinids. The order Torpediniformes was recovered as sister to all other batomorphs, including Apolithabatiformes ord. nov., within which †*K. etchesi and* †*B. sismondae* appear to be more closely related to each other than to †*S. bugesiacus*. However, in light of the recent results of Türtscher et al. (2024) [4], it should be noted that the alleged †*B. sismondae* specimen analyzed by Underwood and Claeson (2019) [53] actually belongs to †*As. platypterus* (CM 4408), and that the three supposed †*S. bugesiacus* specimens belong to †*As. platypterus* (NHMUK PV P 10934), †*Ae. bavarica* (CM 5396), and †*S. bugesiacus* (NHMUK PV P 2099), respectively. This, along with the meanwhile expanded data matrix, explains why the relationships within the Late Jurassic rays, as well as between these taxa and the remaining batomorphs as recovered by Underwood and Claeson (2019) [53], differ profoundly from those obtained by us. In the present study, five monophyletic groups are recovered within the superorder Batomorphii, i.e., Myliobatiformes, Torpediniformes, Rajiformes, Rhinopristiformes, and Apolithabatiformes ord. nov., which includes the genera †*Aellopobatis*, †*Apolithabatis* gen. nov., †*Asterodermus*, †*Belemnobatis*, †*Kimmerobatis*, and †*Spathobatis*. The new order Apolithabatiformes is recovered in a sister-group relationship with the remaining batomorphs (including Torpediniformes) and thus represents a member of stem group batomorphs. Similar results were obtained by Villalobos-Segura et al. (2022) [25], as well as by Villalobos-Segura et al. (2019) [48] and Jambura et al. (2023) [28]. The latter two studies will not be discussed in detail here as the data matrix of Villalobos-Segura et al. (2022) [25] is essentially an extension of that of Villalobos-

Segura et al. (2019) [48], while that of Jambura et al. (2023) [28] focuses on sharks rather than batomorphs, and adopted the coding for batomorphs from Villalobos-Segura et al. (2022) [25].

Despite the similar placement of the Late Jurassic batomorph clade, the topology recovered in the present study differs from that of Villalobos-Segura et al. (2022) [25], not least because we have added two newly described species. Furthermore, given the limited knowledge of Late Jurassic ray taxonomy at the time of the study by Villalobos-Segura et al. (2022) [25], several specimens were assigned to what we now know are the wrong species and genera. The present study is therefore the first to analyze specimens that undoubtedly belong to the species †*As. platypterus*, †*B. sismondae*, †*K. etchesi*, and †*S. bugesiacus*, as well as the recently described species †*Ae. bavarica* (Türtscher et al., 2024 [4]) and the here newly described †*Ap. seioma* gen. et sp. nov. Based on the observations of Türtscher et al. (2024) [4] and the present study, we changed the coding of the Late Jurassic batomorph clade of Villalobos-Segura et al. (2022) [25], on whose data matrix our phylogenetic analysis is based, which also contributed to a changed topology. Below, we explain in detail the most important changes made (see character list for all changes).

Pectoral fin radials (character 83): As the coding of Villalobos-Segura et al. (2022) [25] for †*S. bugesiacus* is mainly based on specimens that were later ascribed to the new species †*Ae. bavarica* (see Türtscher et al., 2024 [4]), we had to change the coding for the character 'pectoral fin radials' (character 114 in Villalobos-Segura et al., 2022 [25]); while in †*Ae. bavarica* (as well as in †*Ap. seioma* gen. et sp. nov., †*As. platypterus*, †*B. sismondae*, and †*K. etchesi*) all radials articulate with the three basal cartilages, †*Spathobatis bugesiacus* is the only known Late Jurassic

ray in which a radial articulates directly with the scapulocoracoid. We therefore coded this character for †*S. bugesiacus* as 'some articulate directly with scapulocoracoid' (see Supplemental Material 2).

Postpelvic processes (character 91): The strict consensus tree estimated by Villalobos-Segura et al. (2022) [25] recovered two synapomorphies of Late Jurassic batomorphs, i.e. the presence of an elongated mesopterygium (character 106 in Villalobos-Segura et al., 2022 [25]) and the presence of postpelvic processes (character 118 in Villalobos-Segura et al., 2022 [25]). In light of the new taxonomic diversity of Late Jurassic batomorphs recovered by Türtscher et al. (2024) [4], we re-examined all currently known taxa and agree with Villalobos-Segura et al. (2022) [25] that this group shares the presence of distally projecting mesopterygia that follow the contour of the propterygia. However, we disagree with Villalobos-Segura et al.'s (2022) [25] coding of the postpelvic processes, which are small and shallow rounded processes on the posteromedian margin of the puboischiadic bar (da Silva et al., 2021 [54]). This character was originally considered a synapomorphy for members of the family Platyrhinidae (Nishida, 1990 [55]; McEachran et al., 1996 [56]; McEachran & Aschliman, 2004 [57]; Aschliman et al., 2012 [58]), but was later shown to be present in some Torpediniformes and Rhinopristiformes (da Silva et al., 2021 [54]). According to the study by Villalobos-Segura et al. (2022) [25], postpelvic processes are present in Jurassic batomorphs, Torpediniformes (except *Narke* and *Temera*), Platyrhinidae, Rhinopristiformes (except *Pristis*, *Rhina*, and *Rhynchobatus*), and *Hemiscyllium*. According to da Silva et al. (2021) [54], postpelvic processes are absent in *Aptychotrema*, but we were able to clearly identify them as present in the material examined, in agreement with the interpretation of Villalobos-Segura et al. (2022) [25]. However, Villalobos-Segura et al. (2022) [25] coded this character as

present in the Late Jurassic batomorphs †*Asterodermus*, †*Belemnobatis*, †*Kimmerobatis*, and †*Spathobatis*, and recovered the presence of postpelvic processes as a synapomorphy for a Late Jurassic batomorph clade. Our revision of the Late Jurassic batomorphs revealed the presence of the processes only in †*Spathobatis*, while the other Late Jurassic batomorphs, including the recently described †*Aellopobatis* as well as †*Apolithabatis* gen. nov. did not show this character (see Supplemental Material 2).

Two dorsal fin spines (character 134): Villalobos-Segura et al. (2022) [25] coded dorsal fin spines as absent in †*S. bugesiacus* and as unknown ('?') in †*As. platypterus* (character 130 in Villalobos-Segura et al., 2022 [25]). The supposed †*S. bugesiacus* specimens they examined included three specimens that were previously included in †*S. bugesiacus*, but which we now know to be †*Ae. bavarica* (see Türtscher et al., 2024 [4]) and which do indeed lack dorsal fin spines, and two specimens of †*S. bugesiacus*, one of which lacks the trunk and one of which is a fossil imprint and does not show the minute fin spines. However, re-examination of †*S. bugesiacus* by Türtscher et al. (2024) [4] clearly confirmed that two small fin spines are present in front of each dorsal fin, so we changed the coding of this character to 'present' in †*S. bugesiacus*. Most †*As. platypterus* specimens lack the caudal fin, dorsal fins, and fin spines. In the holotype (NHMUK PV P12067), however, the very small fin spines are preserved, and we therefore have changed the coding for the character 'two dorsal fin spines' to 'present' as well (see Supplemental Material 2).

Enameloid layer on fin spines (character 135): In accordance with the coding change of character 134 (see above), we also changed the coding of the cohesive

character 'enameloid layer on fin spines' to 'present' (character 131 in Villalobos-Segura et al., 2022 [25]) in †*S. bugesiacus* and †*As. platypterus*.

The modified character scoring and the addition of two new taxa resulted in a less resolved tree under the parsimony criterion than in Villalobos-Segura et al. (2022) [25]. While our phylogenetic tree left the interrelationships of Apolithabatiformes ord. nov. largely unresolved, the MP analysis of Villalobos-Segura et al. (2022) [25] suggested a basal position for †*Belemnobatis* and a sister-group relationship between †*Asterodermus* and †*Kimmerobatis*. Note that the maximum likelihood (ML) and Bayesian inference (BI) analyses of Villalobos-Segura et al. (2022) [25] resulted in significantly different topologies, highlighting the uncertainty in the phylogenetic placement of Late Jurassic rays.

Similarly, our maximum likelihood analysis yielded a different result, suggesting that Late Jurassic rays form a paraphyletic group (Fig S6). Although the resulting phylogenetic tree was more resolved than in the MP analysis, these results must be treated with caution; the number of model parameters estimated by W-IQ Tree exceeded the number of characters, and thus phylogenetic estimates should be interpreted with caution. However, both ML and MP analyses agreed on the sister-group relationship between †*Apolithabatis* gen. nov. and †*Aellopobatis*.

These ongoing conflicts in batomorph phylogeny demonstrate that our understanding of the evolution of morphological characters and phylogenetic relationships within batomorphs is not yet fully resolved. There are also serious conflicts between morphological and molecular studies on the relationships between different batomorph orders (e.g., Stein et al., 2018 [59]). Furthermore, the ML and BI analyses of Villalobos-Segura et al. (2022) [25] based on morphological characters

show the uncertainty in morphological analyses, with none of the relationships between the different batomorph orders being resolved. A well-supported molecular tree based on whole genomes could provide a backbone for exploring morphological characters in batomorphs, as has been done for sharks (Jambura et al., 2023 [28]). This has the potential to identify unambiguous synapomorphies, confidently reconstruct the evolution of morphological characters, and thus improve our understanding of the evolutionary history of this group. Our knowledge of batomorph systematics in deep time is still far from complete, as many interrelationships, such as those within apolithabatiform or rhinopristiform taxa, remain largely unresolved. Within Rhinopristiformes, in particular the Late Cretaceous Lebanese batomorphs (i.e., †'*Rhinobatos*' *hakelensis*, †'*Rhinobatos*' *latus*, †'*Rhinobatos*' *maronita*, †'*Rhinobatos*' *whitfieldi*, †*Rhombopterygia*) have been identified as rogue taxa due to their highly uncertain position within the order, resulting in a polytomy. Previous studies have attempted to clarify the phylogenetic relationships of Lebanese batomorphs, but have failed to provide conclusive results (Brito & Dutheil, 2004 [60]; Kachacha et al., 2017 [61]). It is evident that the morphology and taxonomy of these taxa are in urgent need of revision in order to obtain a more resolved phylogenetic tree and thus improve our understanding of the evolution of batomorphs.

Conclusion

Apart from an as of yet undescribed batomorph skeleton from the Early Jurassic Posidonia Shale near Holzmaden, Germany (Maisey et al., 2020 [3]), which is currently under description, the Late Jurassic taxa †*Ae. bavarica*, †*As. platypterus*, †*B. sismondae*, †*K. etchesi*, and †*S. bugesiacus* represent the earliest known

batomorphs preserved as articulated skeletons. In this study we identified another Late Jurassic ray species, †*Apolithabatis seioma* gen. et sp. nov., which is the first batomorph described from Painten, Germany. In addition, this upper Kimmeridgian ray is the oldest Late Jurassic batomorph known from Germany based on skeletal remains. A phylogenetic analysis including †*Ap. seioma* gen. et sp. nov. as well as the recently described †*Ae. bavarica* was conducted. The results suggest that †*Ap. seioma* gen. et sp. nov. and †*Ae. bavarica* are more closely related to each other than to the other members of the newly established stem group batomorph order Apolithabatiformes ord. nov., but also that the interrelationships of Late Jurassic rays are still largely unresolved. Although the detailed study of these exquisitely preserved fossils has allowed us to make significant progress in understanding the diversity and phylogenetic relationships of early batomorphs, the results also clearly show that we still face crucial obstacles in establishing robust relationships within the batomorph clade based on morphology, and that it is of great importance to put more effort into character exploration in order to obtain a more complete picture of the evolutionary history of rays and skates.

THIS IS NOT STRESSED OR DISCUSSED EXTENSIVELY IN THE PREVIOUS PARAGRAPHS

Acknowledgements

We are grateful to Raimund Albersdörfer (Dinosaurier Museum Altmühltal, Germany) for allowing us to work on this extraordinary fossil and to the staff of the Dinosaurier Museum Altmühltal for their help in documenting the fossil. We would also like to thank Sebastian Stumpf (University of Vienna, Austria). This research was funded in whole by the Austrian Science Fund (FWF) P33820 and P35357. For

the purpose of open access, the authors have applied a CC BY public copyright licence to any Author Accepted Manuscript version arising from this submission.

References

1. Hubbell G. Using tooth structure to determine the evolutionary history of the white shark. In: Klimley AP, Ainley DG, editors. Great white sharks: the biology of *Carcharodon carcharias*. Academic Press; 1996. pp. 9-18.
2. Maisey JG. What is an 'elasmobranch'? The impact of palaeontology in understanding elasmobranch phylogeny and evolution. J Fish Biol. 2012;80: 918-951.
3. Maisey JG, Ehret DJ, Denton JS. A new genus of Late Cretaceous angel shark (Elasmobranchii; Squatinidae), with comments on squatinid phylogeny. Am Mus Novit. 2020;2020: 1-29.
4. Türtscher J, Jambura PL, Villalobos-Segura E, López-Romero FA, Underwood CJ, Thies D, et al. Rostral and Body shape analyses reveal cryptic diversity of Late Jurassic batomorphs (Chondrichthyes, Elasmobranchii) from Europe. Pap Palaeontol. 2024;e1552. https://doi.org/10.1002/spp2.1552
5. Guinot G, Cavin L. 'Fish' (Actinopterygii and Elasmobranchii) diversification patterns through deep time. Biol Rev. 2016;91: 950-981.
6. Underwood CJ. Diversification of the Neoselachii (Chondrichthyes) during the Jurassic and Cretaceous. Paleobiology. 2006;32: 215-235.
7. Villalobos-Segura E, Stumpf S, Türtscher J, Jambura PL, Begat A, López-Romero FA, et al. A synoptic review of the cartilaginous fishes

(Chondrichthyes: Holocephali, Elasmobranchii) from the Upper Jurassic Konservat-Lagerstätten of Southern Germany: taxonomy, diversity, and faunal relationships. Diversity. 2023;15: 386.

8. Kriwet J, Klug S. Knorpelfische (Chondrichthyes). In: Arratia G, Schultze H-P, Tischlinger H, Viohl G, editors. Solnhofen—Ein Fenster in die Jurazeit, 2. München: Verlag Dr. Friedrich Pfeil; 2015. pp. 334-359.

9. Albersdörfer R, Häckel W. Die Kieselplattenkalke von Painten. In: Arratia G, Schultze H-P, Tischlinger H, Viohl G, editors. Solnhofen—Ein Fenster in die Jurazeit, 2. München: Verlag Dr. Friedrich Pfeil; 2015. pp. 126–133.

10. Viohl G. Die Plattenkalk-Typen der Südlichen Frankenalb. In: Arratia G, Schultze H-P, Tischlinger H, Viohl G, editors. Solnhofen—Ein Fenster in die Jurazeit, 2. München: Verlag Dr. Friedrich Pfeil; 2015. pp. 72–77.

11. Sauvage HE. Notice sur un spathobate du terrain portlandien de Boulogne-sur-Mer. Bull Soc Acad Boulogne-Sur-Mer. 1873;2: 94-103.

12. Cavin L, Cappetta H, Séret B. Révision de *Blemnobatis morinicus* (Sauvage, 1873) du Portlandien du Boulonnais (Pas-de-Calais, France). Comparison avec quelques Rhinobatidae Jurassiques. Geol Palaeontol. 1995;29: 245-267.

13. Cione AL. First report of a Jurassic ray outside of Europe. In: Arratia G, Schultze H-P, editors. Mesozoic fishes 2—Systematics and Fossil Record. München: Verlag Dr. Friedrich Pfeil; 1999. pp. 21-28.

14. Tischlinger H, Arratia G. Ultraviolet light as a tool for investigating Mesozoic fishes, with a focus on the ichthyofauna of the Solnhofen archipelago. In: Arratia G, Schultze H-P, Wilson MVH, editors. Mesozoic fishes 5—Global

Diversity and Evolution. München: Verlag Dr. Friedrich Pfeil; 2013. pp. 549-562.

15. Josse J, Husson F. missMDA: a package for handling missing values in multivariate data analysis. J Stat Softw. 2016;70: 1-31.
16. R Core Team. R: a language and environment for statistical computing. R Foundation for Statistical Computing. 2013. http://www.R-project.org/
17. R Studio Team. RStudio: Integrated Development for R. RStudio, Inc., Boston, MA. 2019. http://www.rstudio.com/
18. Wickham H. ggplot2: Elegant graphics for data analysis. Springer. 2016.
19. Garnier S. viridisLite: colorblind-friendly colour maps (lite version). R package version 0.4.1. 2018. https://cran.r-project.org/web/packages/viridis/index.html
20. Rohlf FJ. tpsDig2, digitize landmarks and outlines, version 2.31. TpsSeries. Stony Brook: SUNY, Department of Ecology and Evolution. 2017.
21. Rohlf FJ, Slice D. Extensions of the Procrustes method for the optimal superimposition of landmarks. Syst Biol. 1990;39: 40-59.
22. Bookstein FL. Landmark methods for forms without landmarks: morphometrics of group differences in outline shape. Med Image Anal. 1997;1: 225-243.
23. Adams DC, Collyer M, Kaliontzopoulou A, Sherratt E. Geomorph: software for geometric morphometric analyses. 2016. https://CRAN.R-project.org/package=geomorph
24. Collyer ML, Adams DC. RRPP: an R package for fitting linear models to high-dimensional data using residual randomization. Methods Ecol Evol. 2018;9: 1772-1779.

25. Villalobos-Segura E, Marramà G, Carnevale G, Claeson KM, Underwood CJ, Naylor GJ, Kriwet J. The phylogeny of rays and skates (Chondrichthyes: Elasmobranchii) based on morphological characters revisited. Diversity. 2022;14: 456.

26. Goloboff PA, Farris JS, Nixon KC. TNT, a free program for phylogenetic analysis. Cladistics. 2008;24: 774-786. https://doi.org/10.1111/j.1096-0031.2008.00217.x

27. Goloboff PA, Morales ME. TNT version 1.6, with a graphical interface for MACOS and Linux, including new routines in parallel. Cladistics. 2023;39: 144-153. https://doi.org/10.1111/cla.12524

28. Jambura PL, Villalobos-Segura E, Türtscher J, Begat A, Staggl MA, Stumpf S, et al. Systematics and phylogenetic interrelationships of the enigmatic Late Jurassic shark *Protospinax annectans* Woodward, 1918 with comments on the shark–ray sister group relationship. Diversity. 2023;15: 311. https://doi.org/10.3390/d15030311

29. Maddison WP, Maddison DR. Mesquite: a modular system for evolutionary analysis. Version 3.81. 2023. http://www.mesquiteproject.org

30. Trifinopoulos J, Nguyen L-T, von Haeseler A, Minh BQ. W-IQ-TREE: A fast online phylogenetic tool for maximum likelihood analysis. Nucleic Acids Res. 2016;44: W232-W235. https://doi.org/10.1093/nar/gkw256

31. Nguyen L-T, Schmidt HA, von Haeseler A, Minh BQ. IQ-TREE: A Fast and Effective Stochastic Algorithm for Estimating Maximum-Likelihood Phylogenies. Mol Biol Evol. 2015;32: 268-274. https://doi.org/10.1093/molbev/msu300

32. Kalyaanamoorthy S, Minh BQ, Wong TKF, von Haeseler A, Jermiin LS. ModelFinder: Fast model selection for accurate phylogenetic estimates. Nat Methods. 2017;14: Article 6. https://doi.org/10.1038/nmeth.4285

33. Hoang DT, Chernomor O, von Haeseler A, Minh BQ, Vinh LS. UFBoot2: improving the ultrafast bootstrap approximation. Mol Biol Evol. 2018;35: 518-522.

34. Huxley TH. On the application of the laws of evolution to the arrangement of the vertebrate and more particularly of the Mammalia. Proc Zool Soc Lond. 1880;1880: 649-662.

35. Bonaparte CL. Selachorum tabula analytica. Nuovi Ann Sci Nat Bologna. 1838;2: 195-214.

36. Hay OP. Bibliography and catalogue of the fossil Vertebrata of North America. Bull US Geol Geograph Surv Terr. 1902;179: 1-868.

37. Compagno LJV. Phyletic relationships of living sharks and rays. Am Zool. 1977;17: 303-322.

38. Cappetta H. Les Sélaciens du Crétacé supérieur du Liban. II. Batoïdes. Palaeontogr Abt A. 1980;168: 149-229.

39. Zittel KA von. Handbuch der Paläontologie. I. Abtheilung Paläozoologie. III Band. Vertebrata (Pisces, Amphibia, Reptilia, Aves). München, Leipzig: R Oldenbourg. 1887-1890.

40. Woodward AS. Catalogue of the fossil fishes in the British Museum (Natural History). Part I. Containing the Elasmobranchii. British Museum (Natural History) Department of Geology. 1889a.

41. Woodward AS. IV, – Note on *Rhinobatus bugesiacus* – a Selachian Fish from the Lithographic Stone. Geol Mag. 1889b;6: 393-396.

42. Thies D. Placoid scales (Chondrichthyes: Elasmobranchii) from the Late Jurassic (Kimmeridgian) of northern Germany. J Vertebr Paleontol. 1995;15: 463-481.

43. Underwood CJ, Ward DJ. Neoselachian sharks and rays from the British Bathonian (Middle Jurassic). Palaeontology. 2004;47: 447-501.

44. Kriwet J, Nunn EV, Klug S. Neoselachians (Chondrichthyes, Elasmobranchii) from the Lower and lower Upper Cretaceous of north-eastern Spain. Zool J Linn Soc. 2009;155: 316-347.

45. Thies D, Leidner A. Sharks and guitarfishes (Elasmobranchii) from the Late Jurassic of Europe. Palaeodiversity. 2011;4: 63-184.

46. Klug S, Kriwet J. An offshore fish assemblage (Elasmobranchii, Actinopterygii) from the Late Jurassic of NE Spain. Paläontol Z. 2013;87: 235-257.

47. Dames W. K. A. Zittel: Handbuch der Palaeontologie. 1. Abtheil. Palaeozoologie. III. Bd. 1 Lief. München 1887. N Jb Min Geol Paläont. 1888;1888: 128-132. https://biodiversitylibrary.org/page/44314187

48. Villalobos-Segura E, Underwood CJ, Ward DJ, Claeson KM. The first three-dimensional fossils of Cretaceous sclerorhynchid sawfish: *Asflapristis cristadentis* gen. et sp. nov., and implications for the phylogenetic relations of the Sclerorhynchoidei (Chondrichthyes). J Syst Palaeontol. 2019;17: 1847-1870.

49. Villalobos-Segura E, Underwood CJ. Radiation and divergence times of Batoidea. J Vertebr Paleontol. 2020;40: e1777147.

50. de Carvalho MR. A Late Cretaceous Thornback Ray from Southern Italy, with a Phylogenetic Reappraisal of the Platyrhinidae (Chondrichthyes:

Batoidea). In: Arratia G, Tintori A, editors. Mesozoic Fishes 3—Systematics, Paleoenvironments and Biodiversity. München: Verlag Dr. Friedrich Pfeil; 2004. pp. 75-100.

51. Underwood CJ. Sharks, rays and a chimaeroid from the Kimmeridgian (Late Jurassic) of Ringstead, southern England. Palaeontology. 2002;45: 297-325.

52. Leuzinger L, Cuny G, Popov E, Billon- Bruyat JP. A new chondrichthyan fauna from the Late Jurassic of the Swiss Jura (Kimmeridgian) dominated by hybodonts, chimaeroids and guitarfishes. Pap. Palaeontol. 2017;3: 471-511.

53. Underwood CJ, Claeson KM. The Late Jurassic ray *Kimmerobatis etchesi* gen. et sp. nov. and the Jurassic radiation of the Batoidea. Proc Geol Assoc. 2019;130: 345-354.

54. da Silva JPCB, Silva Loboda T, de Souza Rosa R. A new synapomorphy in the pelvic girdle reinforces a close relationship of *Zanobatus* and Myliobatiformes (Chondrichthyes: Batoidea). J Anat. 2021;238: 874-885.

55. Nishida K. Phylogeny of the suborder Myliobatidoidei. Mem Fac Fish Sci Hokkaido Univ. 1990;37: 1-108.

56. McEachran JD, Dunn KA, Miyake T. Interrelationships of the batoid fishes (Chondrichthyes: Batoidea). In: Stiassny MLJ, Parenti LR, Johnson GD, editors. Interrelationships of fishes. San Diego, London: Academic Press; 1996. pp. 63-84.

57. McEachran JD, Aschliman N. Phylogeny of Batoidea. In: Carrier JC, Musick JA, Heithaus MR, editors. Biology of Sharks and their Relatives. Boca Raton, Florida: CRC Press; 2004. pp. 79-113.

58. Aschliman NC, Claeson KM, McEachran JD. Phylogeny of Batoidea. In: Carrier JC, Musick JA, Heithaus MR. Biology of Sharks and Their Relatives. Boca Raton, Florida: CRC Press; 2012. pp. 57-95.

59. Stein RW, Mull CG, Kuhn TS, Aschliman NC, Davidson LN, Joy JB, Smith GJ, Dulvy NK, Mooers AO. Global priorities for conserving the evolutionary history of sharks, rays and chimaeras. Nat Ecol Evol. 2018;2: 288-298.

60. Brito PM, Dutheil DB. A preliminary systematic analysis of Cretaceous guitarfishes from Lebanon. In: Arratia G, Tintori A, editors. Mesozoic Fishes 3—Systematics, Paleoenvironments and Biodiversity. München: Verlag Dr. Friedrich Pfeil; 2004. pp. 101-109.

61. Kachacha G, Cuny G, Azar D, Abi Saad P. Revision of the fossil batomorphs from the Cretaceous of Lebanon, and their impact on our understanding of the early step of the evolution of the clade. Research & Knowledge. 2017;3: 33-37.

Supporting information

00_Supplemental material content.pdf: Overview of Supplemental material

01_Supplemental tables.xlsx: Tables S1-S18

02_Supplemental figures.pdf: Figures S1-S3

03_Supplemental material 1.pdf: Traditional morphometrics [% DL] Results, Figures S4-S5, Tables A-G

04_Supplemental material 2 - Character list.docx: Character list [including explanations and three figures]

05_Supplemental material 2 - Matrix without rogues.nex: Data matrix without rogue taxa

06_Supplemental material 2 - Script TNT unconstrained.tnt: TNT Script

07_Supplemental material 2 - Log file.log: Log file

08_Supplemental material 2 - Figure S6.pdf: Figure S6.

Figure 1

Click here to access/download;Figure;01_Figure.tif

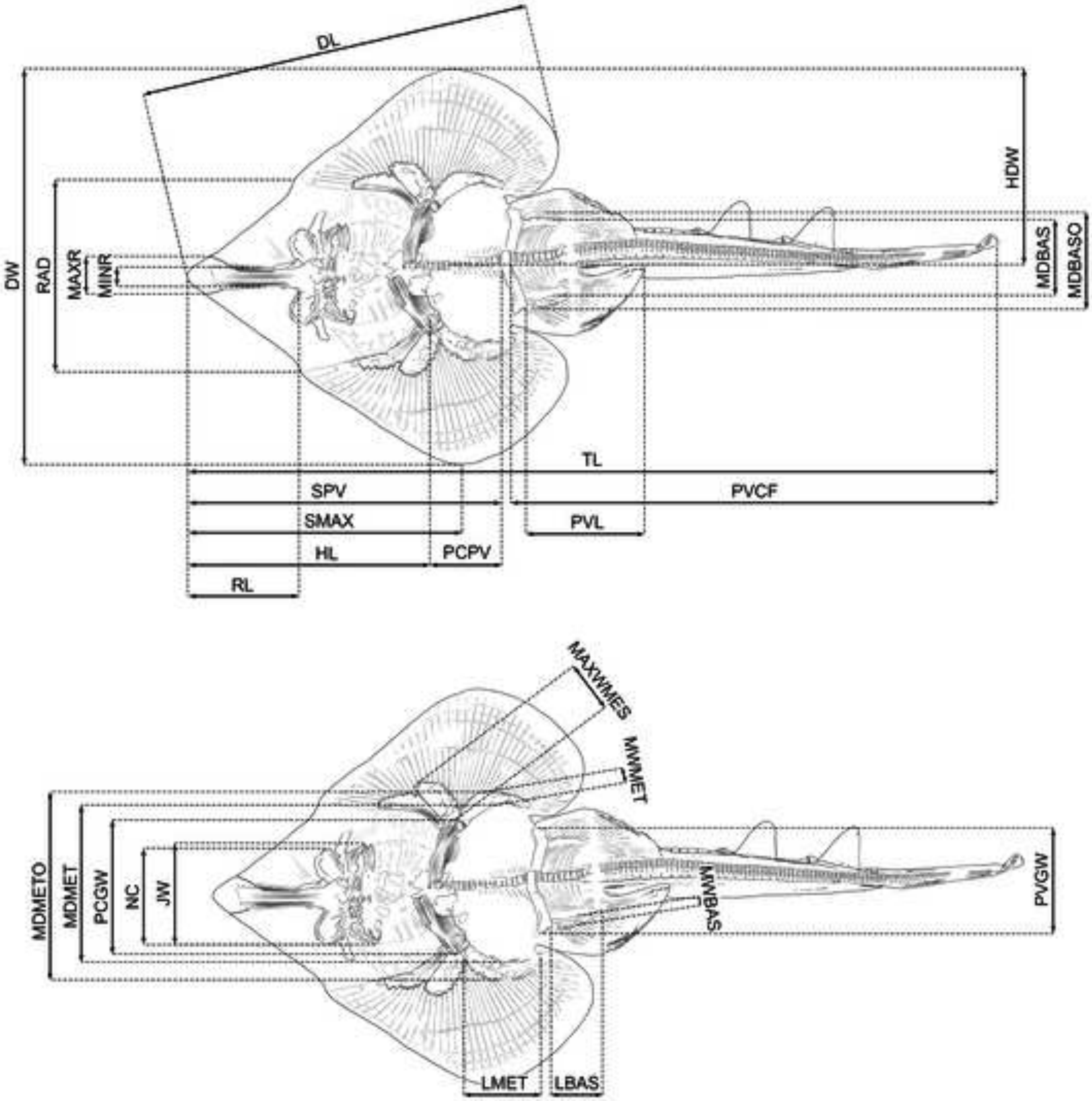

Figure 2

Click here to access/download;Figure;02_Figure.tif

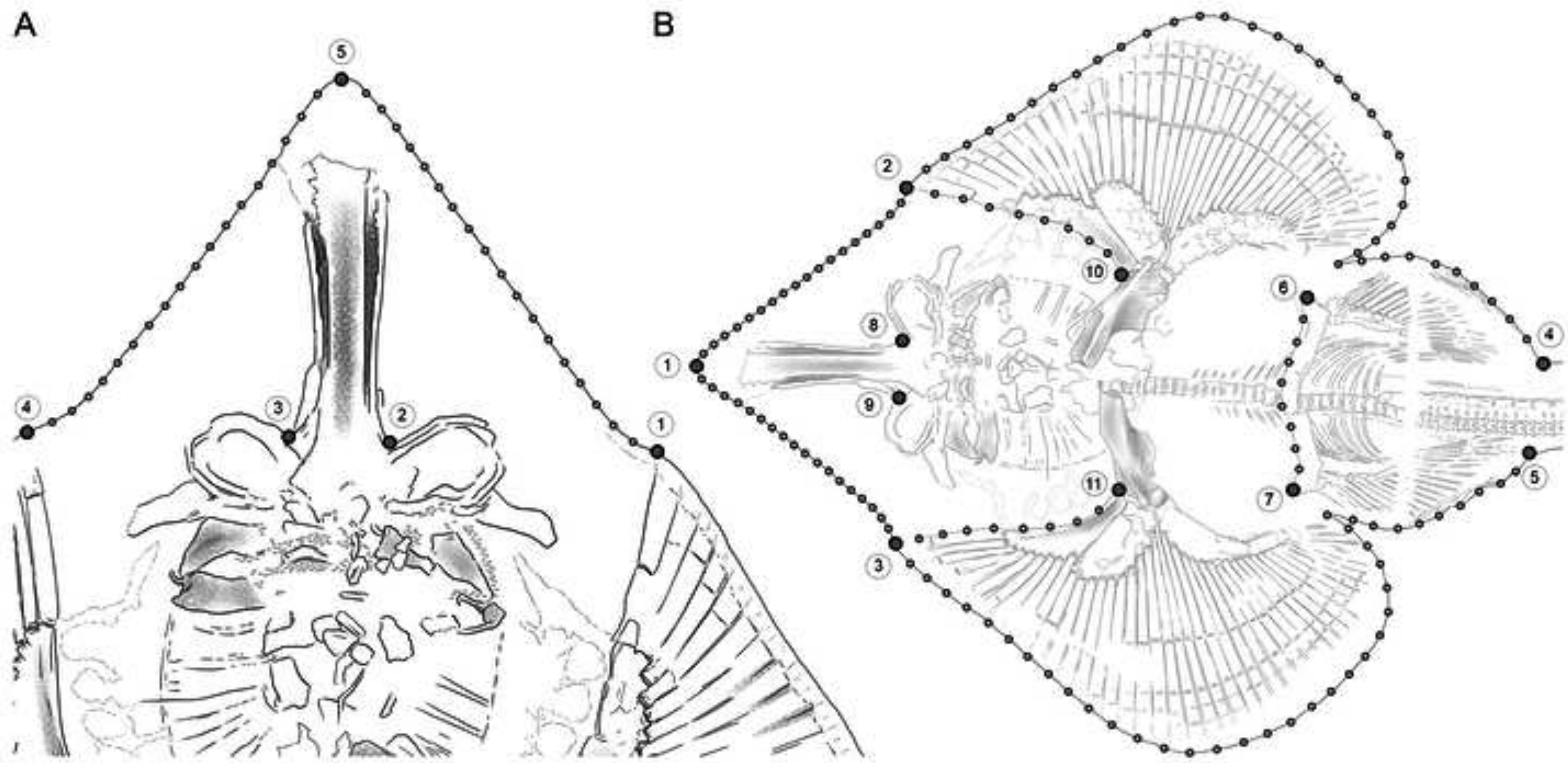

Figure 3

Click here to access/download;Figure;03_Figure.tif

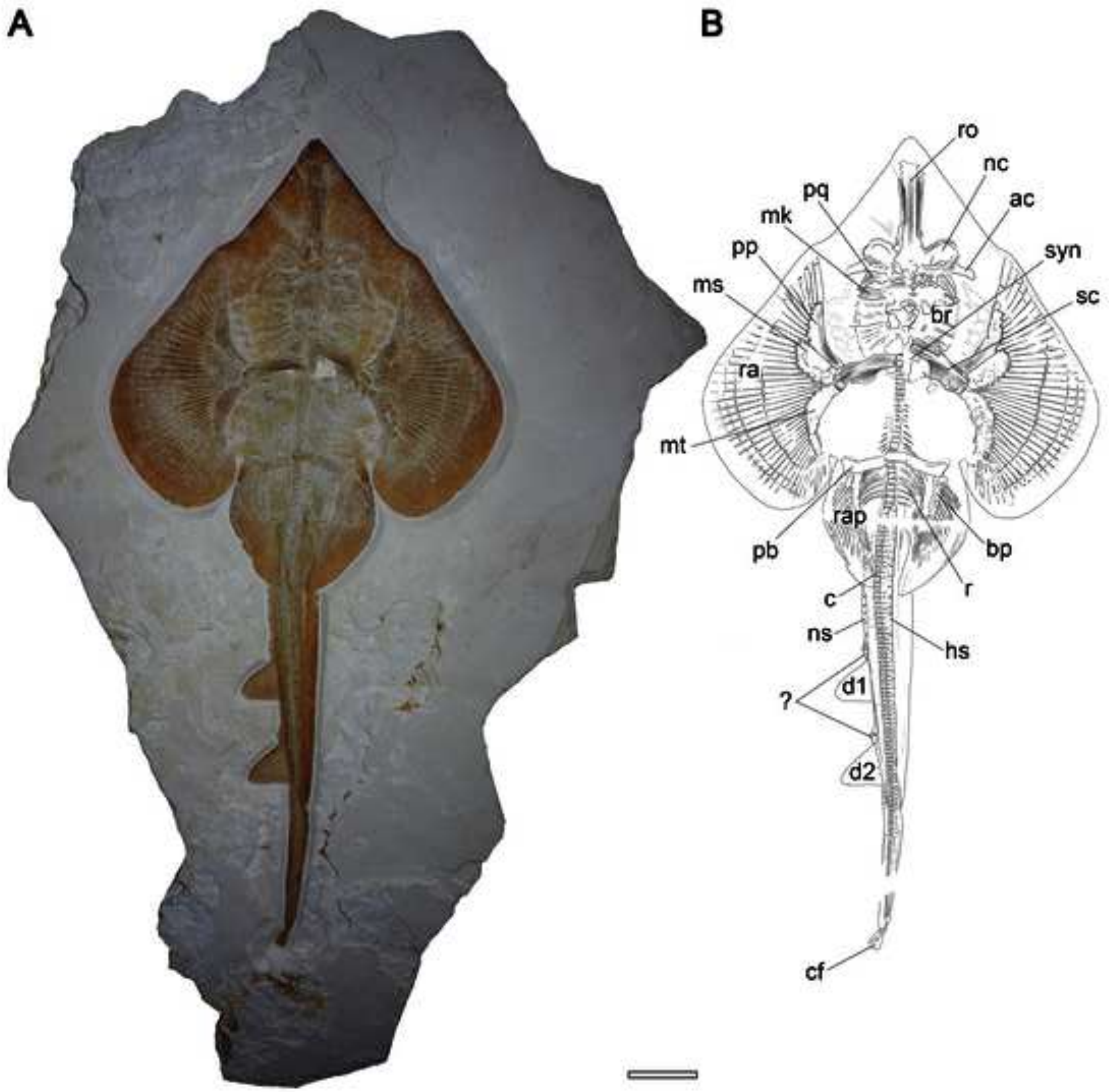

Figure 4

Click here to access/download;Figure;04_Figure_head.tif

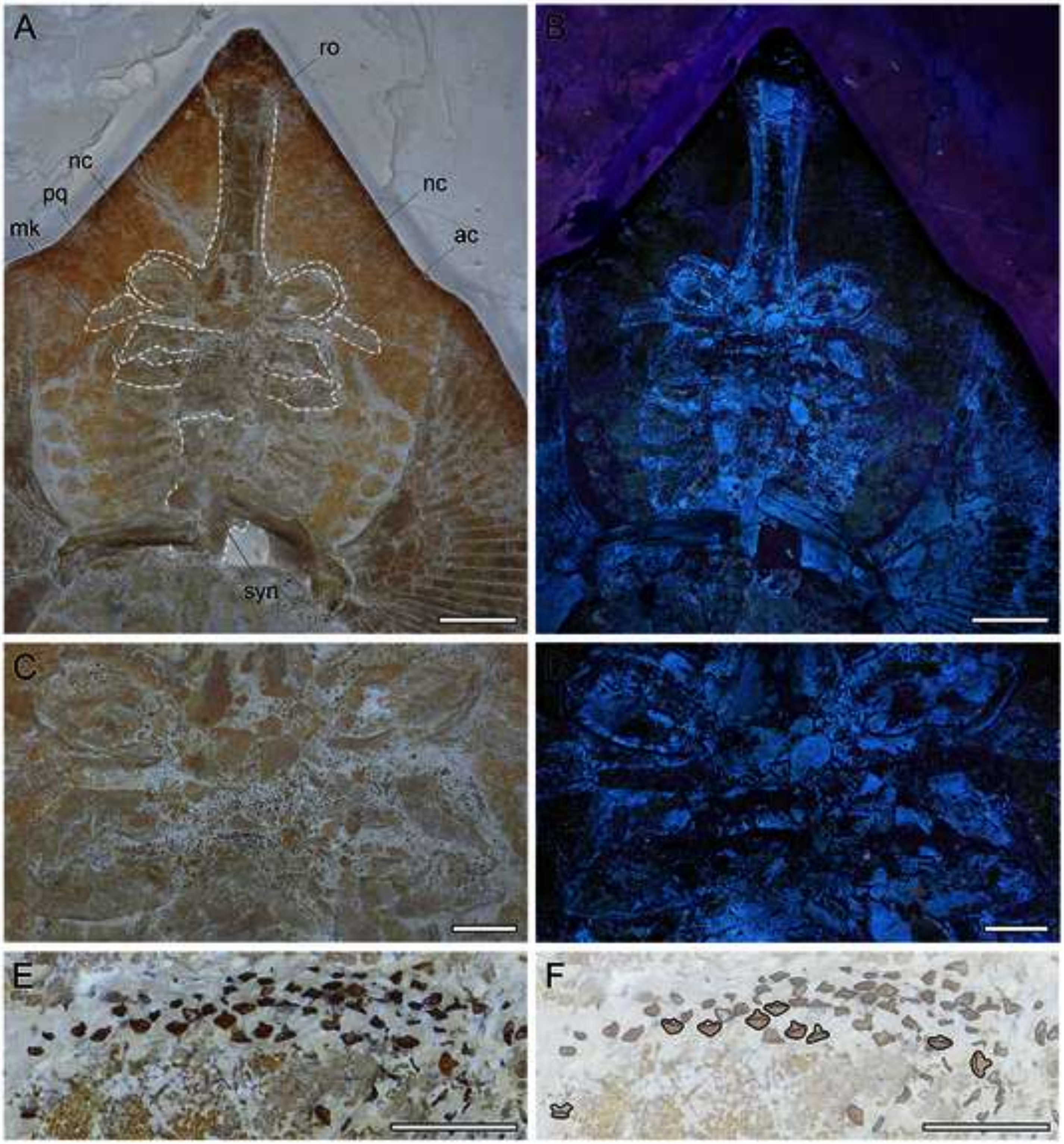

Figure 5

Click here to access/download;Figure;05_Figure_pectoral_fin.tif

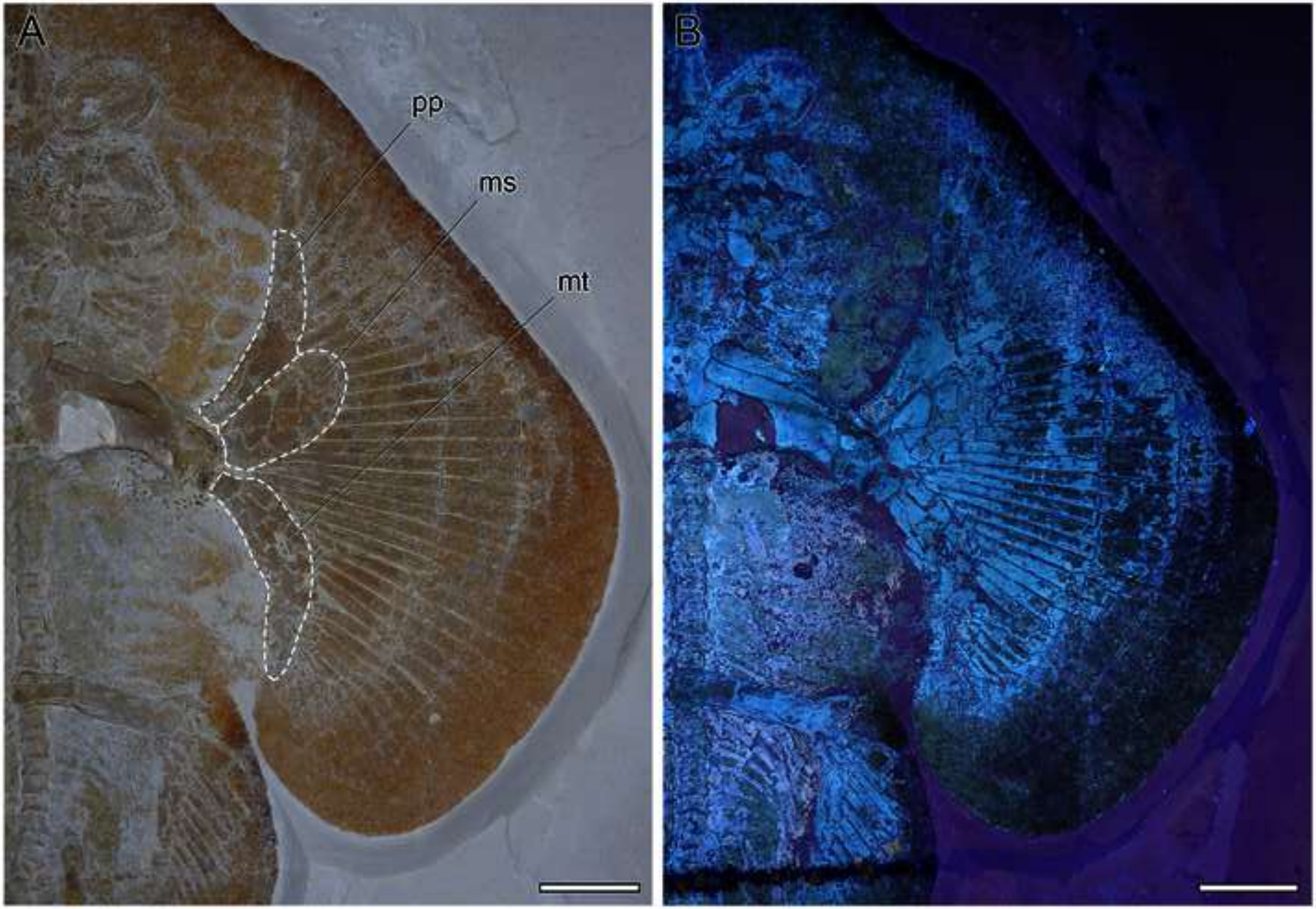

Figure 6

Click here to access/download;Figure;06_Figure_trunk_tail.tif

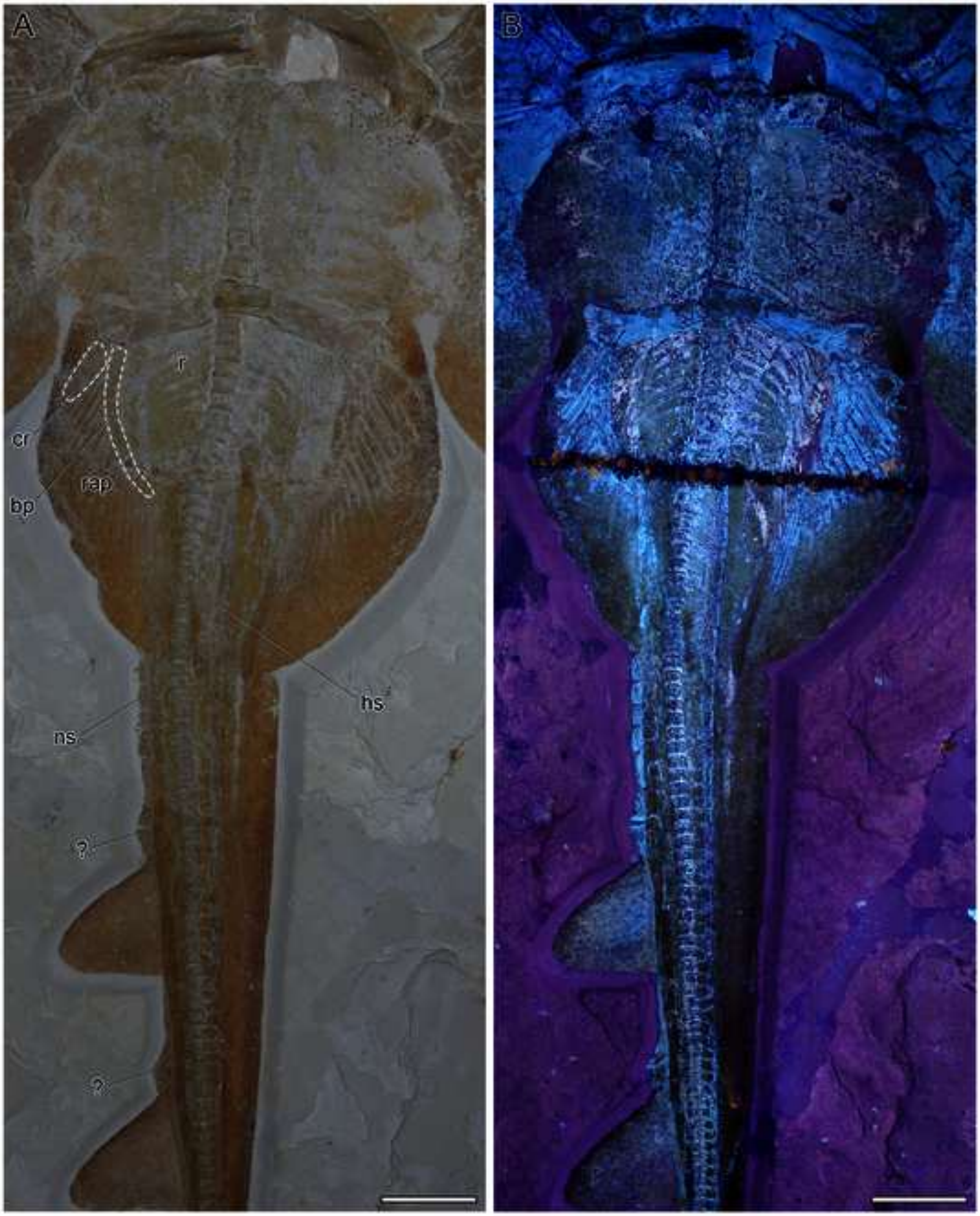

Figure 7

Click here to access/download;Figure;07_Figure_TM.tif

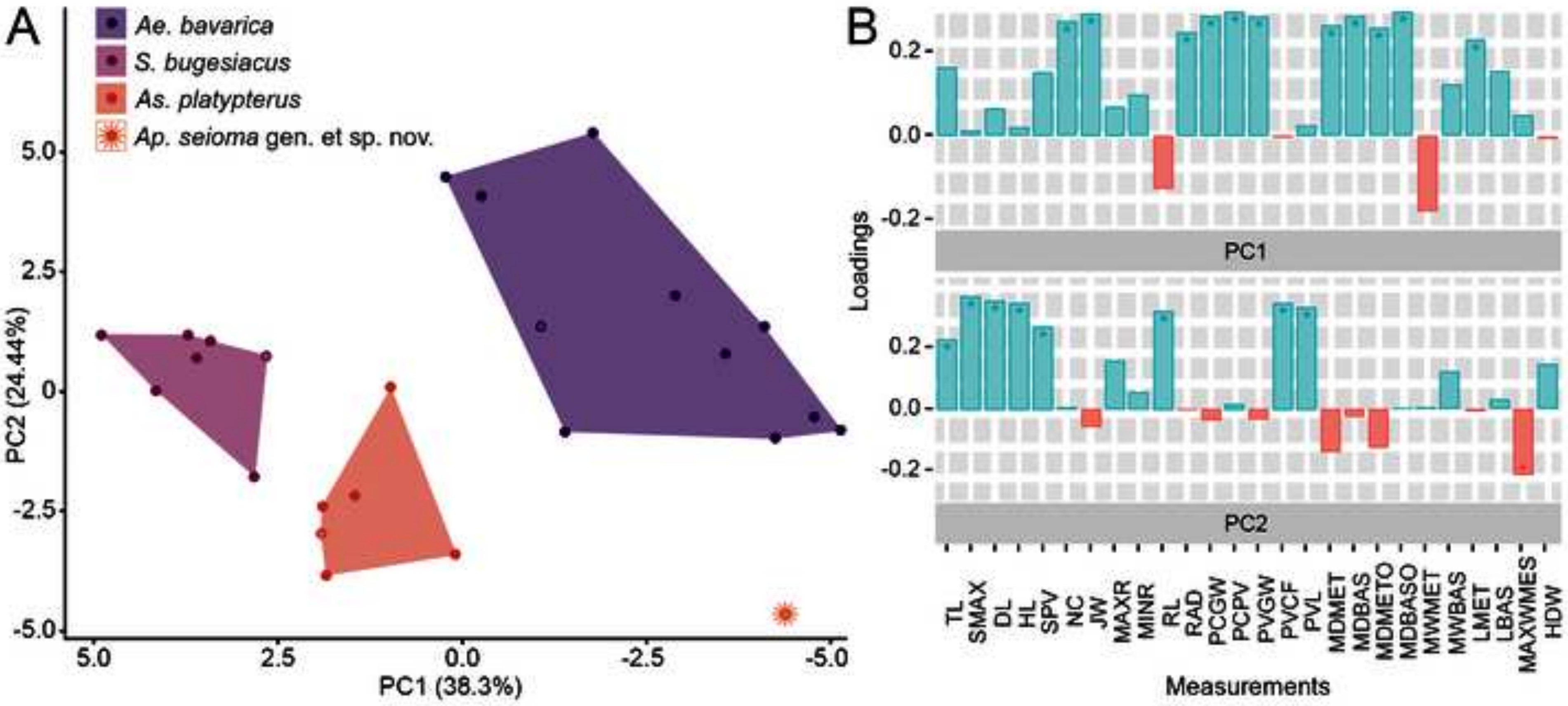

Figure 8

Click here to access/download;Figure;08_Figure_TM_loadings_PC1-PC2.tif

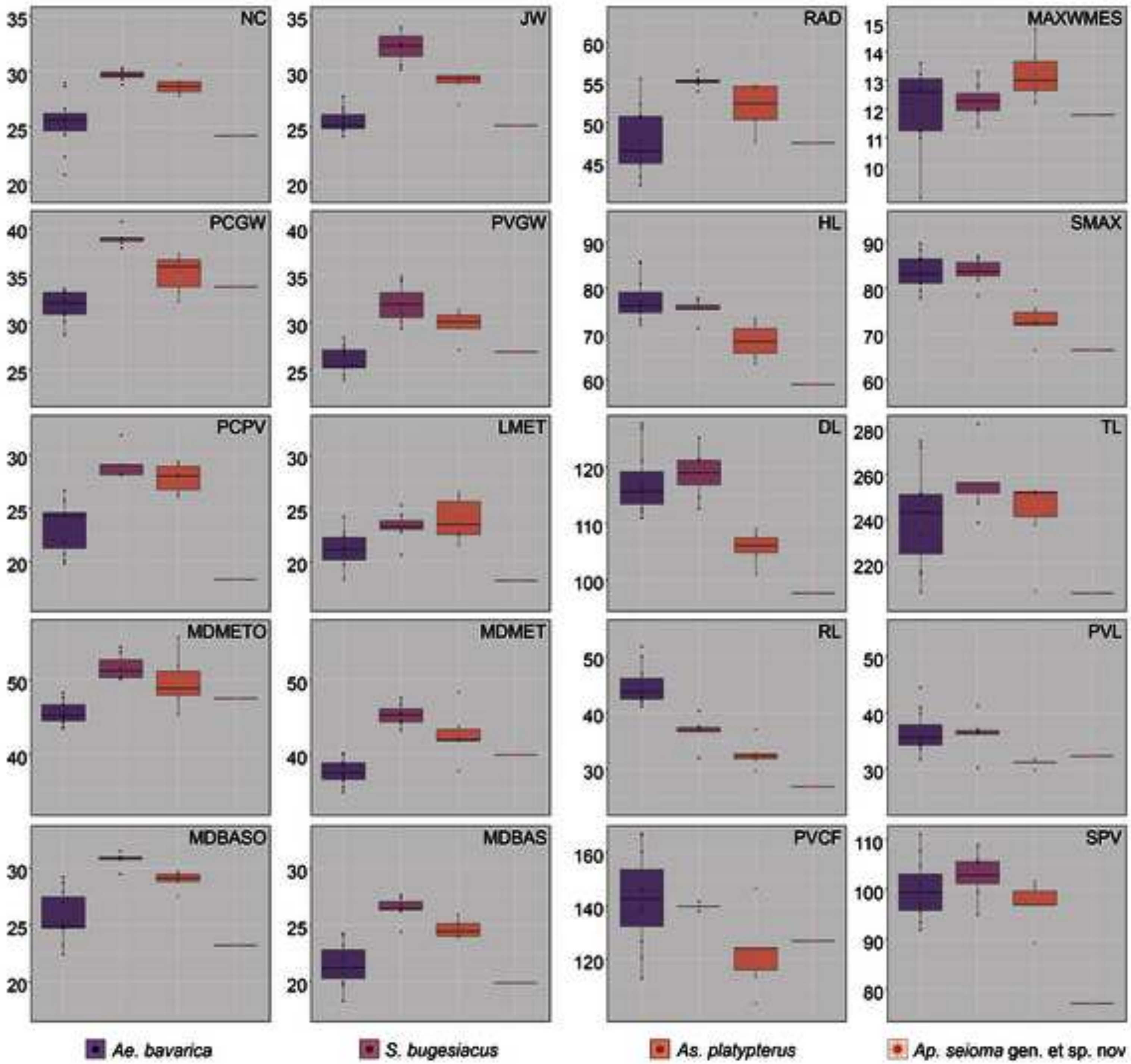

Figure 9

Click here to access/download;Figure;09_Figure_GM_head-outline.tif

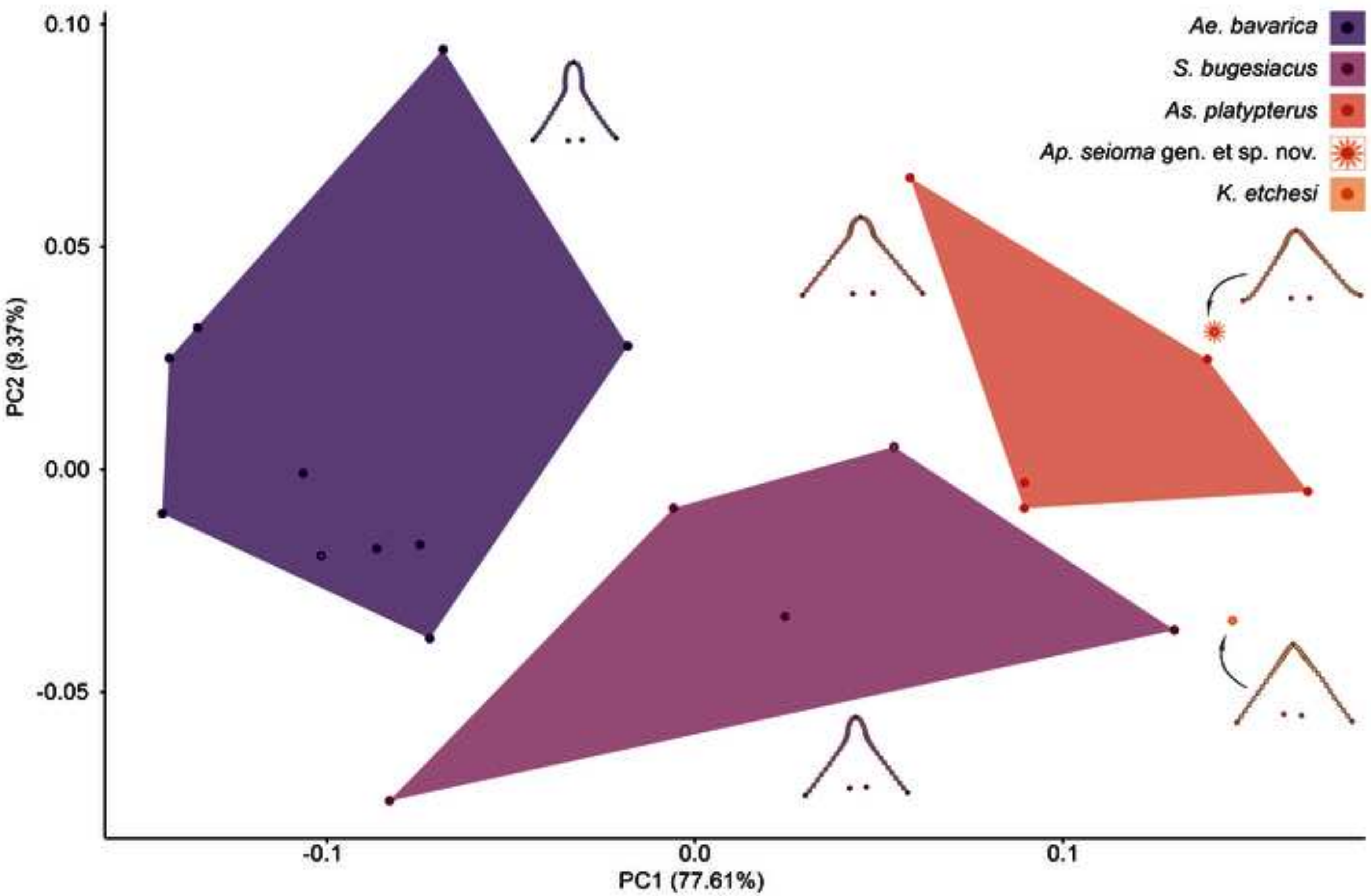

Figure 10

Click here to access/download;Figure;10_Figure_GM_complete-body.tif

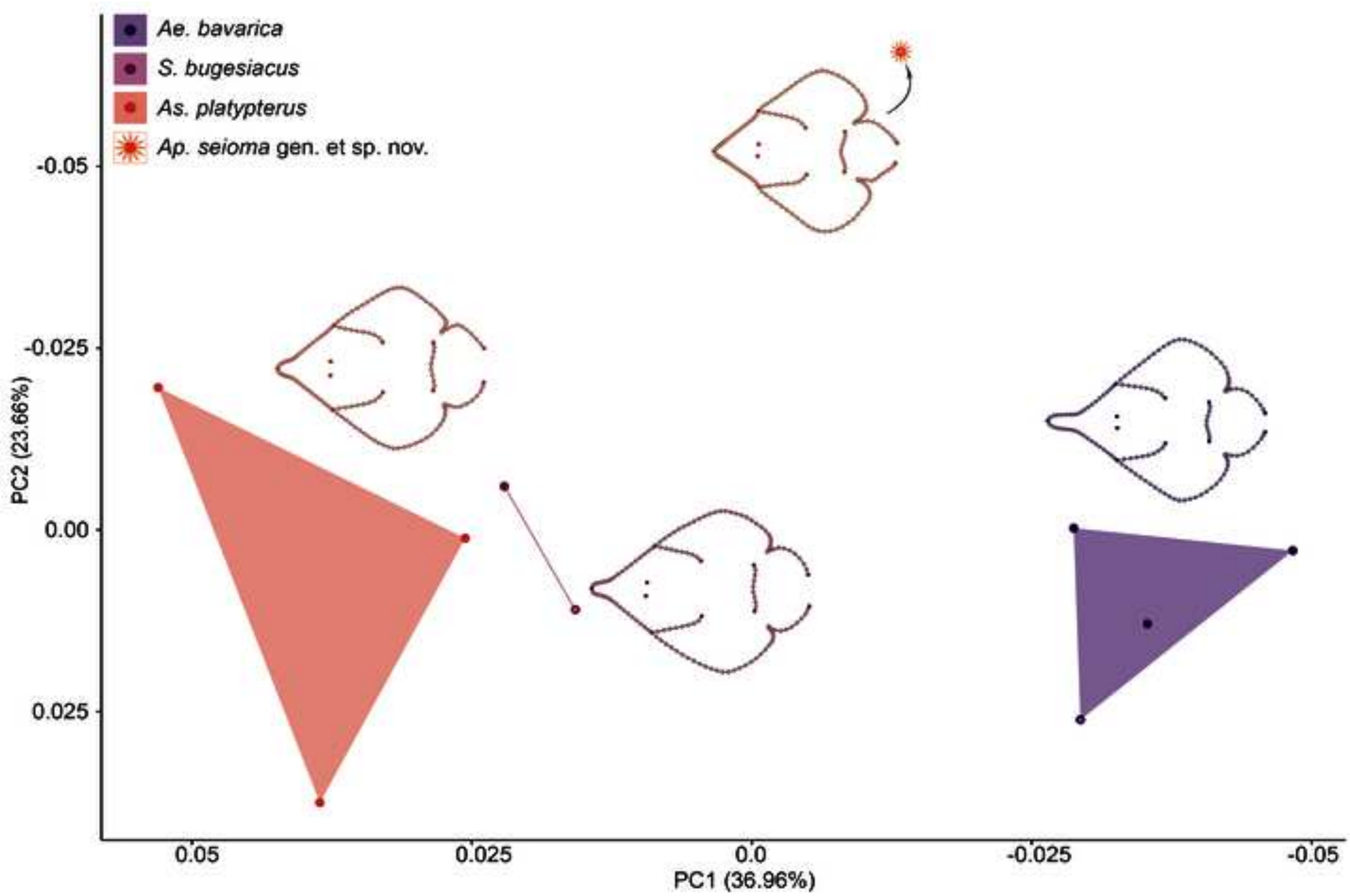

Figure 11

Click here to access/download;Figure;11_Figure_Phylogeny.tif

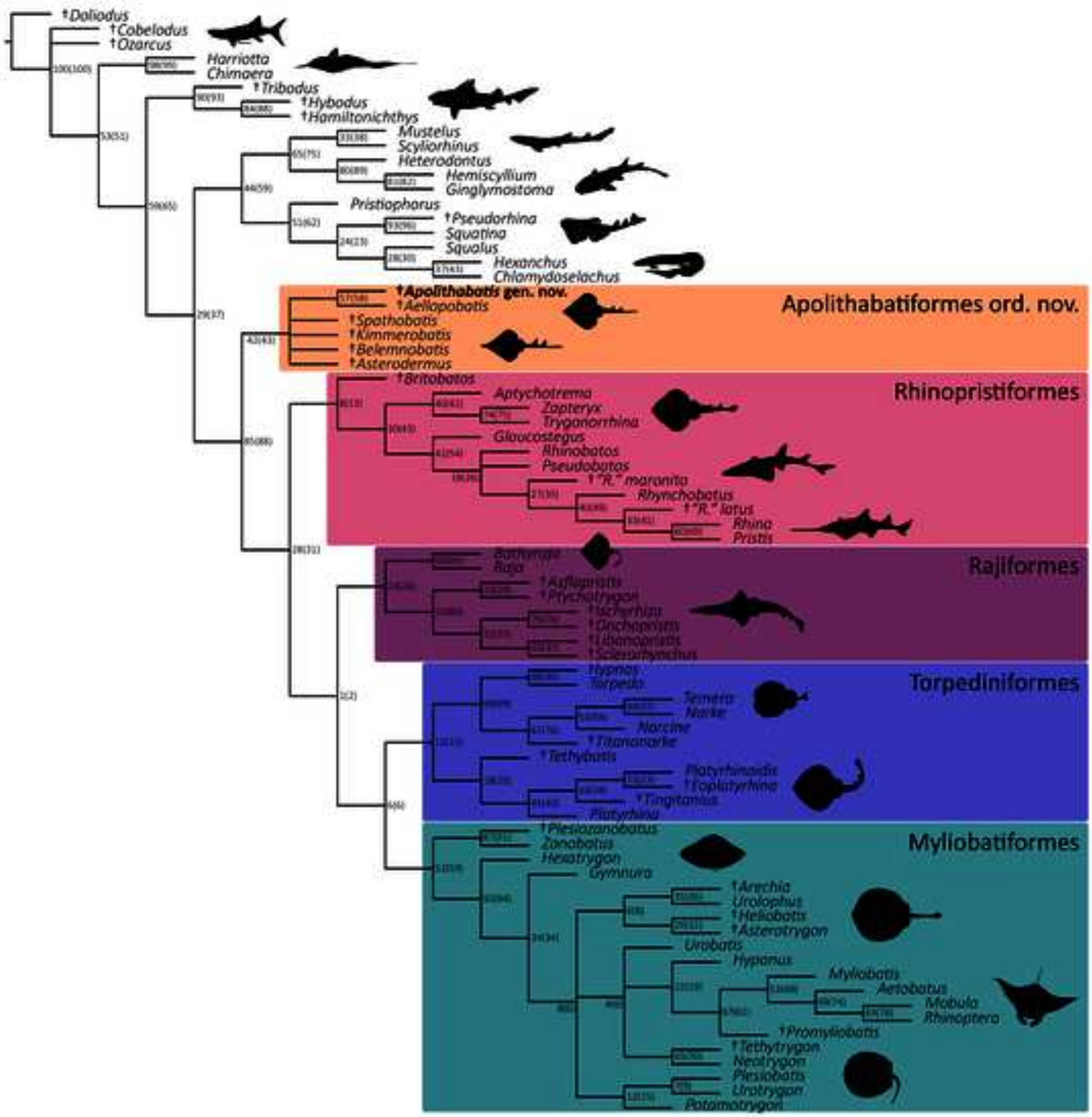

Figure 12

Click here to access/download;Figure;12_Figure_Apolithabatis_Acrosaurus.jpg

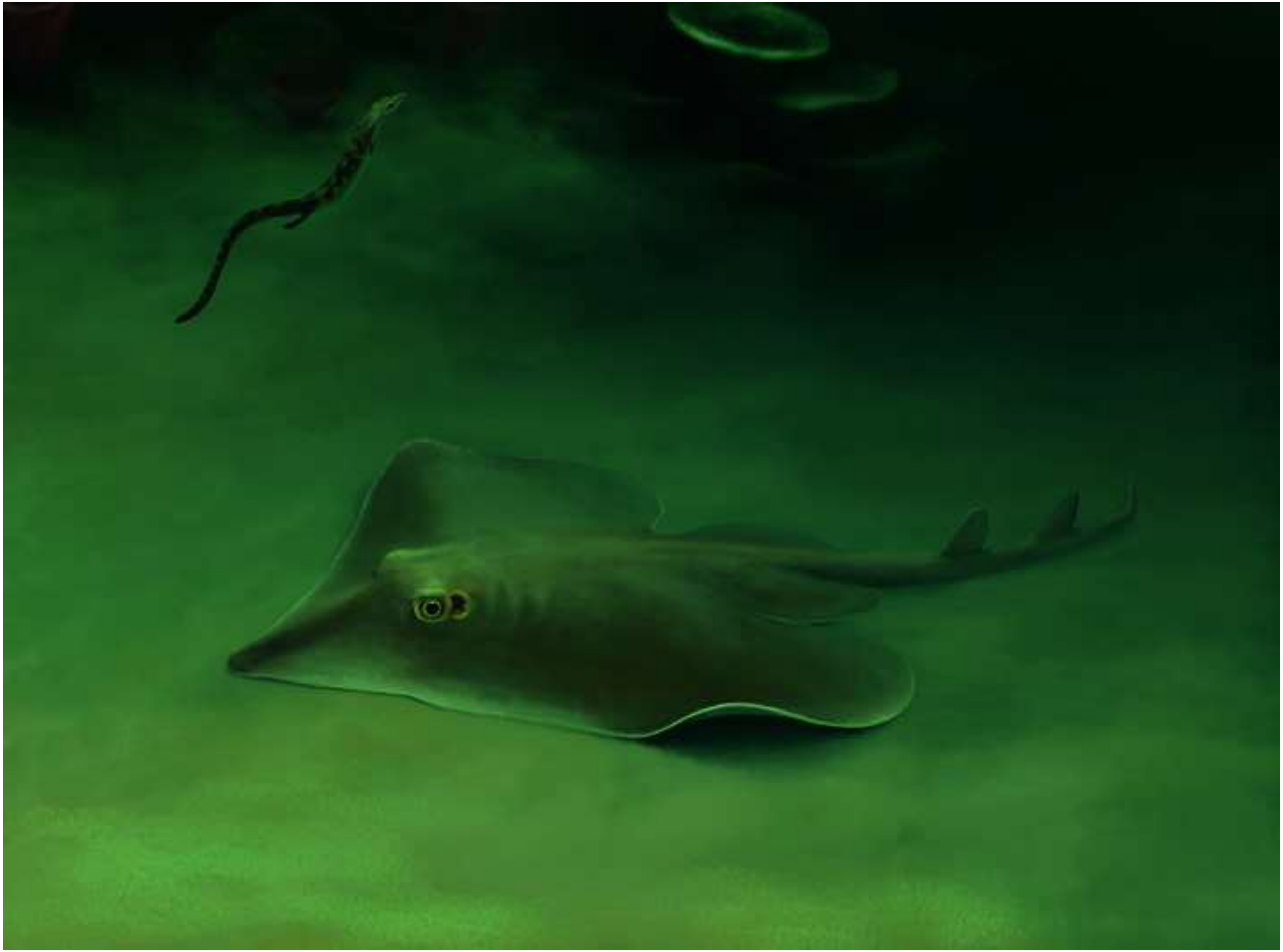

Click here to access/download
Supporting Information
00_Supporting material content.pdf

Click here to access/download
Supporting Information
01_Supporting tables.xlsx

Click here to access/download
Supporting Information
02_Supporting figures.pdf

Click here to access/download
Supporting Information
03_Supporting material 1.pdf

Click here to access/download
Supporting Information
04_Supporting material 2 - Character list.docx

Click here to access/download
Supporting Information
05_Supporting material 2 - Matrix without rogues.nex

Click here to access/download
Supporting Information
06_Supporting material 2 - Script TNT unconstrained.tnt

Click here to access/download
Supporting Information
07_Supporting material 2 - Log file.log

Click here to access/download
Supporting Information
08_Supporting material 2 - Figure S6.pdf
