# Supplementary material for: Insights into stem Batomorphii: A new holomorphic ray (Chondrichthyes, Elasmobranchii) from the upper Jurassic of Germany
Source: PLoS One. 2025 Jan 23;20(1):e0310174. doi: 10.1371/journal.pone.0310174 (PMC11756912; doi:10.1371/journal.pone.0310174)
Supplement: Attachment — Submitted filename: review comments_JA.docx [file pone.0310174.s012.docx]

Dear Editor and Authors,

The study reports of a new batomorph species from the Painten locality (Solnhofen Arcipelago, Upper Jurassic). Data are presented in an appropriate fashion and statistics are more than adequate. The inferences are well-supported and the language is fluent. All in all, this study is of international and broad interest and provides novel and vey significant information.

There are only some minor points I would like to raise:

- I would recommend inserting a figure for the geological/paleogeographic/geographic setting to better display all the localities of the Solnhofen Arcipelago and the relationships with Painten. It would be also reasonable to include a stratigraphic scheme of the quarry with indication of the level of provenance of the new taxon.

- There are minor typos in the manuscript, please check all the issues throughout the whole manuscript, I might have missed some of them (indications in the annotated reviewer attachment)

- Landmarks are described in a caption, I would recommend describing them in a paragraph in the method section or in supplementaries and shortening the caption.

- Please consider inserting a paragraph with salient diagnostic characters of the new order as indicated in the annotated attachment, just as a brief summary of the character discussed.

- line 578-582: please consider diving the sentences, it is quite complex.

- The discussion section does not include any hint of the stratigraphic distribution of the new taxon in comparison with the other identified taxa, while in the conclusion paragraph it is said that the new taxon is the oldest Jurassic batomorph. I would recommend emphasizing and discussing extensively this in the discussion paragraph, I think it could be significant. Is there any pattern in the stratigraphic distribution of the mentioned taxa? Also a figure could be useful for the comparison.

- The phylogenetic hypothesis is very interesting and the authors also stressed it to be treated with caution, but I think that the result is significant. I would recommend emphasizing also in the conclusion paragraph that the new order should be treated as a working hypothesis, unresolved for still undescribed material from other Lagerstatten; it might be redundant but I think it is always good.

I would be pleased to answer any question from the authors and be open to any discussion.

Best regards,

Jacopo Amalfitano, PhD
